# Single‐Cell Profiling Reveals RAB13 ^+^ Endothelial Cells and Profibrotic Mesenchymal Cells in Aged Human Bone Marrow

**DOI:** 10.1111/acel.70475

**Published:** 2026-04-09

**Authors:** Itziar Cenzano, Miguel Cócera, Ana Rosa Lopez‐Perez, Lorea Campos‐Dopazo, Javier Ruiz, Ignacio Sancho, Patxi San Martin‐Uriz, Paula Aguirre‐Ruiz, Sarai Sarvide, Amaia Vilas‐Zornoza, Purificacion Ripalda‐Cemborain, Diego Alignani, Aitziber Lopez, Marta Miñana Barrios, Delia Quilez Agreda, Jin Ye, Robert Lehmann, Laura Sudupe, Marta Abengozar‐Muela, Álvaro Lopez‐Janeiro, Luis‐Esteban Tamariz‐Amador, Emma Muiños‐López, Borja Saez, Jesper Tegner, Isabel A. Calvo, David Gomez‐Cabrero, Felipe Prosper

**Affiliations:** ^1^ Hematology and Oncology Program, Centre for Applied Medical Research (CIMA), Cancer Center Clinica Universidad de Navarra (CCUN) Pamplona Navarra Spain; ^2^ Bioscience Program, Biological and Environmental Sciences and Engineering Division (BESE), King Abdullah University of Science and Technology (KAUST) Thuwal Saudi Arabia; ^3^ Instituto de Investigaciones Sanitarias de Navarra (IdiSNA) Pamplona Spain; ^4^ Centro de Investigacion Biomedica en Red de Cancer (CIBERONC). Madrid Madrid Spain; ^5^ Translational Bioinformatics Unit, Navarrabiomed, Universidad Pública de Navarra (UPNA) Pamplona Spain; ^6^ Hospital Universitario de Navarra Pamplona Spain; ^7^ Hospital Reina Sofía de Tudela Navarra Spain; ^8^ Departament of Pathology, Clinica Universidad de Navarra Pamplona Spain; ^9^ Hematology and Cell Therapy Department. Clínica Universidad de Navarra Pamplona Spain; ^10^ Computer Science Program, Electrical and Mathematical Sciences and Engineering Division King Abdullah University of Science and Technology (KAUST) Thuwal Saudi Arabia; ^11^ Unit of Computational Medicine, Department of Medicine, Center for Molecular Medicine Karolinska Institutet, Karolinska University Hospital Stockholm Sweden; ^12^ Science for Life Laboratory Solna Sweden

**Keywords:** aging, endothelial cells, human bone marrow microenvironment, profibrotic, single‐cell RNA sequencing, stromal cells

## Abstract

The bone marrow (BM) microenvironment plays a crucial role in regulating hematopoiesis, yet the molecular changes associated with aging in humans remain poorly understood. Using single‐cell RNA sequencing, we uncovered transcriptional shifts in BM endothelial cells (EC) and mesenchymal stromal cells (MSC) during aging. Aged sinusoidal EC exhibited a prothrombotic phenotype with compromised mitochondrial and vascular function. Additionally, we identified a novel arterial EC subset, emerging in aged individuals, characterized by RAB13 expression and associated with transcriptional regulatory processes. MSC from aged subjects displayed impaired matrix remodeling and epithelial‐mesenchymal transition, driven partly by a subpopulation of THY1^+^ profibrotic cells absent in younger individuals. Finally, immunofluorescent imaging and spatial transcriptomics confirmed the presence of these aging‐associated cells in BM samples from aged individuals. In summary, this work provides a comprehensive view of the transcriptional landscape, cellular interactions, and spatial organization of aged EC and MSC, offering novel insights and potential targets that could be exploited for preventing age‐associated changes in humans.

## Graphical abstract

Integrated single‐cell, spatial transcriptomic, and immunofluorescence analyses reveal profound aging‐associated remodeling of the non‐immune human bone marrow niche. Aging is marked by endothelial dysfunction, emergence of RAB13^+^ arterial endothelial cells, and expansion of profibrotic THY1^+^ mesenchymal cells, identifying potential cellular targets for therapeutic intervention in age‐related bone marrow decline.

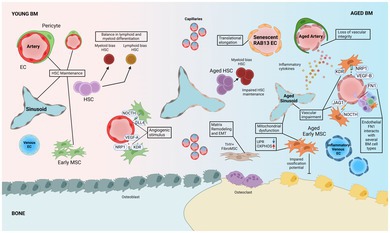

## 1 Introduction

Hematopoietic stem cells (HSC) are the fundamental pillar of the hematopoietic system, sustaining blood production for the entire lifespan of an organism (Eaves 2015). The HSC reside within a specialized bone marrow (BM) microenvironment called niche (Schofield 1978). The HSC niche consists of a complex and dynamic molecular network of interactions among hematopoietic and non‐hematopoietic cells, extracellular matrix (ECM) components, and soluble factors, all working together to ensure proper hematopoiesis (Morrison and Scadden 2014; Pinho and Frenette 2019; Fröbel et al. 2021).

Aging induces a progressive decline of the anatomical and physiological function of all organ systems (Khan et al. 2017) and is the major risk factor across different diseases. In the hematopoietic system, aging is characterized by an increased number of HSC accompanied by reduced self‐renewal capacity and regenerative potential (Su et al. 2024). Over the past decades, several molecular mechanisms that control the HSC aging process have been revealed, yet our understanding remains incomplete (Guo et al. 2022). What has been established is that the senescent phenotype results from the combination and interplay of the intrinsic aging of the HSC and the detrimental effects of an aged microenvironment (Guo et al. 2022; Ramalingam et al. 2023). The study of the microenvironment has demonstrated that aging causes a reduction in the number of endothelial cells (EC), significant disorganization of vascular structures, decreased angiogenic potential, and increased vascular leakiness and reactive oxygen species (ROS) (Poulos et al. 2017; Ho and Méndez‐Ferrer 2020; Dobner et al. 2024). Additionally, aged mesenchymal stromal cells (MSC) exhibit impaired differentiation into osteoblasts and adipocytes and secretion of pro‐inflammatory cytokines influencing the skewed myeloid differentiation of aged HSC (Al‐Azab et al. 2022; Fujino et al. 2022). Therefore, to fully comprehend and possibly mitigate the effects of aging in the hematopoietic system, it is necessary to dissect the complex interactions between aged HSC and their aged non‐hematopoietic microenvironment.

In that direction, recent studies have shed light on remodeling the non‐hematopoietic BM microenvironment in mice during aging (Helbling et al. 2019; Chen et al. 2024). However, translating these findings to humans has been challenging, primarily due to difficulties in adapting methodologies from mouse models and obtaining high‐quality human samples with adequate EC and MSC populations (Wang et al. 2021; Ye et al. 2022; Li et al. 2023; Fiévet et al. 2023; Bandyopadhyay et al. 2024). Consequently, these limitations have restricted the scope of single‐cell analyses in the non‐hematopoietic BM compartment.

In this article, we profiled EC and MSC at both single‐cell and spatial resolution from human BM samples of young and elderly healthy donors. Our analysis reveals that aged sinusoidal EC display a prothrombotic profile and impaired mitochondrial and vascular functions. We identify an aged arterial‐like EC subset marked by RAB13 expression, which is linked to dysregulated transcriptional elongation activity. In the stromal compartment, aging drives matrix remodeling mediated partly by THY1^+^ profibrotic stromal cells, increases oxidative phosphorylation (OXPHOS), and shows a reduced unfolded protein response. Furthermore, we explore the spatial context of these alterations using immunofluorescent imaging and spatial transcriptomics. These spatial approaches confirm the presence of RAB13^+^ EC in aged samples and uncover significant age‐related disruptions in cell–cell communication within the BM niche. This comprehensive analysis offers new insights into the transcriptomic alterations that occur during aging in the human BM.

## 2 Results

### 2.1 Transcriptional Profiling of Human Young and Aged BM Endothelial and Stromal Cells

**FIGURE 1:**
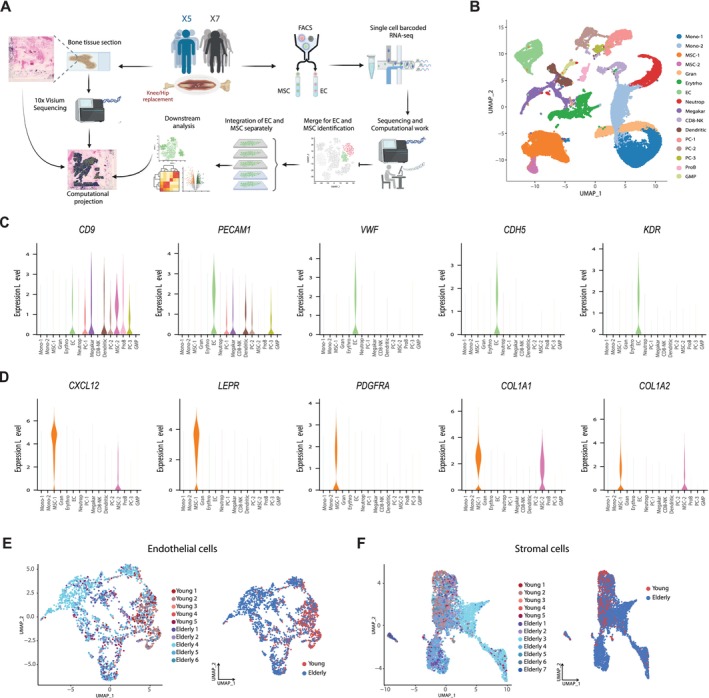
Transcriptional profiling of human young and aged BM endothelial and stromal cells. (A) Overview of the experimental design for isolation, single‐cell RNA sequencing (scRNA‐seq), and spatial transcriptomic analysis of EC and MSC from human BM samples. (B) UMAP representation of human BM microenvironment cells from young and elderly donors, colored by cluster. Right panel: Original UMAP split into young and aged BM datasets. (C,D) Violin plots showing the expression for EC (C) and MSC (D) per cluster. (E,F) Left panel: UMAP projection of the distribution of samples for EC (E) and MSC (F). Right panel: UMAP projection of cells colored by age for EC (E) and MSC (F).

To investigate age‐related changes in the human non‐hematopoietic BM microenvironment, we performed single‐cell transcriptomics on prospectively fluorescence‐activated cell‐sorted EC and MSC from BM tissue of healthy young (< 50 years of age; mean 37.8) and aged (> 58 years of age; mean 68.5) individuals undergoing orthopedic surgery (Figure 1A, Figure S1 and Table S1). After initial quality control and pre‐processing, we obtained a total of 35,368 cells across all samples (14,283 from young and 21,085 from aged individuals), which, despite our sorting strategy, included contaminant hematopoietic cells.

Therefore, we first identified and manually annotated the distinct clusters based on the expression of well‐known marker genes (Figure 1B, Figure S2A,B). EC (cluster EC, *n* = 2159 cells) were identified based on the expression of pan‐EC markers such as *CD9*, *PECAM1*, *VWF, CDH5*, and *KDR*, and MSC were identified (clusters MSC‐1 and MSC‐2, *n* = 5317 cells) based on the expression of MSC and osteolineage (OLN)‐primed markers including *CXCL12*, *LEPR*, *PDGFRA*, *COL1A1*, and *COL1A2* (Figure 1C,D). The remaining clusters were assigned to well‐known hematopoietic BM populations, such as monocytes, granulocyte precursors, erythroblasts, neutrophils, plasma cells (PC), platelets/megakaryocytes, CD8‐natural killer‐like T cells, dendritic cells (DC), proB cells and granulocyte‐monocyte progenitor (GMP), based on the expression of their respective markers (Figure S2C). Notably, the post‐annotation resulting cell‐type‐specific signatures summarized in Table S2 can serve as a valuable resource for identifying or isolating cell types within BM resident populations, thereby enabling the development of more precise sorting strategies.

To focus on the cells of interest, we separately analyzed EC and MSC from both age groups. Additional filtering and validation were performed to confirm the identity of EC and MSC based on canonical marker expression (Figure S2D,E). UMAPs reveal heterogeneous expression of CD9, CD31, and VWF, providing additional confirmation that the isolated cells originated from BM tissue and were not contaminant EC from muscle or periosteal tissue (Boueya et al. 2024). In total, our dataset comprised 2163 EC (549 young, 1614 aged cells) and 5470 stromal cells (808 young, 4662 aged cells) (Figure 1E,F, Figure S2F,G). Overall, these results demonstrate the feasibility of using BM tissue derived from orthopedic surgeries to successfully isolate and analyze the transcriptional signatures of low‐frequency BM EC and MSC in humans.

### 2.2 Aging Is Associated With a Pro‐Inflammatory Program in EC and the Emergence of RAB13 ^+^ Cells

**FIGURE 2:**
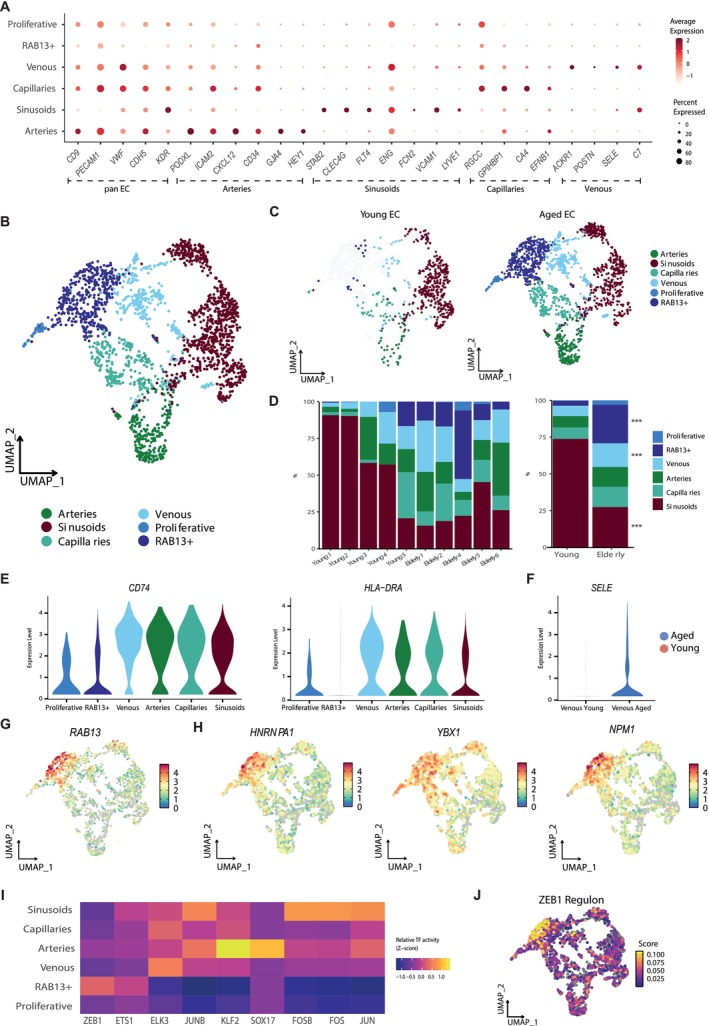
Aging is associated with a pro‐inflammatory program in EC and the emergence of RAB13^+^ cells. (A) Dot plot of markers for EC, arteries, sinusoids, capillaries, and venous used to define EC vascular beds (B) UMAP depicting vascular subtypes identified within the human BM endothelial compartment. (C) UMAP visualization of the vascular beds distribution in young (left) and aged (right) EC. (D) Stacked bar plots showing the proportions of the different vascular states in young and aged EC per sample (left) and age group (right). Asterisks indicate significant differences in the cell proportions between the two groups (FDR < 0.05). (E) Violin plots showing the expression of genes upregulated in the venous cluster. (F) Violin plot displaying the expression of *SELE* in young and aged venous EC. (G) UMAP plot revealing the gene expression of *RAB13* within EC. (H) UMAP plots showing the expression of genes highly expressed in RAB13^+^ cells. (I) Heatmap representing z‐scaled mean regulon activity score of inferred top‐specific regulons for each EC vascular state. (J) UMAP plot showing the activity score of ZEB1 regulon in EC.

To uncover transcriptional differences between EC from young and aged individuals, we first distinguished the major endothelial vascular beds based on the expression of known molecular markers and gene signatures described previously (Ye et al. 2022; Bandyopadhyay et al. 2024; Kalucka et al. 2020; Barnett et al. 2024) (Figure 2A, Figure S3A). Arterial cells were characterized by the expression of *PODXL1*, *ICAM2*, *CXCL12, CD34*, *GJA4*, and *HEY1*, whereas sinusoids overexpressed *STAB2*, *CLEC4G*, *FLT4*, *ENG*, *FNC2*, *VCAM1*, and *LYVE1* (Figure 2A). Beyond characterizing arteries and sinusoids, we identified previously undescribed vascular beds in the human BM, including capillaries and venous vessels (Figure 2B and Table S3). Capillaries were defined by the significant upregulation of known markers, including *RGCC*, *GPIHBP1*, *CA4*, and *EFNB1* (Figure 2A). The venous cluster showed markers of activated post‐capillary venules such as *ACKR1*, *POSTN*, *SELE*, and *C7*, alongside higher expression of other genes like *VWF* involved in coagulation and hemostasis (Thiriot et al. 2017) (Figure 2A). Additionally, we observed a group of EC in a proliferative state (Figure S3B), along with an arterial‐like EC subset marked by the expression of *RAB13*, which we designated as RAB13^+^ cells (Figure S3C). We further confirmed through multiple bioinformatic robustness analyses that this subset was not driven by cell number imbalance or donor‐specific effects. The functional specialization of each EC cluster was further characterized using *Gene Ontology* (GO) *biological process*‐based overrepresentation analysis (ORA) (Ashburner et al. 2000) (Figure S3D).

Given the diversity of vascular beds, we first examined how aging affects the composition of EC subtypes in human BM. To estimate robustly the statistical significance, we conducted a permutation‐based analysis, and to account for inter‐individual variability, we employed bootstrapping to robustly estimate confidence intervals. Our analysis revealed age‐dependent shifts in EC cluster distribution (Figure 2C, Figure S4A). Specifically, we observed a significant reduction in sinusoidal EC (odds ratio (OR) = 0.93, 95% confidence interval (CI): 0.90–0.96, *p* = 0.00014) and an increase in the venous (OR = 1.04, 95% CI: 1.01–1.07, *p* = 0.012) and the RAB13^+^ EC subset (OR = 1.11, 95% CI: 1.04–1.19, *p* = 0.004) in aged individuals (Figure 2D, Figure S4B). The venous cluster was defined by an increased expression of genes related to “antigen processing and presentation” and “leukocyte activation involved in immune response” (*CD74*, *HLA–DRA, HLA–DQB1, TFF3*), suggesting an imbalanced immune function and elevated pro‐inflammatory response during aging (Figure 2E, Figure S4C). Besides being a marker of the venous cluster, *SELE*, one of the major drivers of prothrombotic endothelial activation and leukocyte extravasation during inflammation (Hopkin et al. 2021), was highly expressed in aged venous EC and may serve as a potential biomarker for EC aging (Figure 2F, Figure S4D).

Interestingly, our study identified the emergence of a transcriptionally distinct subset of EC in aged individuals, defined by high expression of *RAB13*, a GTPase associated with cellular senescence (Wechter et al. 2023) (Figure 2G, Figure S4E). These RAB13^+^ EC exhibited transcriptional similarities to arterial cells but could be distinguished by elevated expression of genes associated with mRNA stability and translation, including *HNRNPA1*, *YBX1*, and *NPM1* (Figure 2H and Table S4). Functional enrichment analysis of RAB13^+^ cells revealed an upregulation of ribosomal biogenesis, characterized by increased expression of ribosomal binding proteins (RBP) and translation‐related processes (Table S4 and Figure S4F,G). These findings align with recent studies implicating ribosomes in the aging process (Varesi et al. 2023). In addition, we observed gene sets associated with Myc targets (*NPM1, HNRNPA1, NAP1L1*, and *EEF1B2*) and cellular senescence (*RAB13, VIM*, *SPARC*, *CCND1*, and *NME2*) in RAB13^+^ cells (Figure S4H).

To better understand the molecular programs underlying this age‐related subset, we inferred the active regulons in the RAB13^+^ arterial‐like EC population using SCENIC (Aibar et al. 2017) and further assessed these predictions using SimiC (Peng et al. 2022). Interestingly, our computational analysis identified the ZEB1 regulon as a potential candidate regulator activated in RAB13^+^ EC (Figure 2I,J, Figure S4I). The ZEB1 transcription factor (TF) is a known inducer of epithelial‐mesenchymal transition (EMT) and has been identified as a critical regulator of HSC functionality and differentiation (Almotiri et al. 2024). Moreover, recent evidence suggests that Zeb1 induces quiescence of endothelial progenitors during the establishment of vascular homeostasis (Yu et al. 2022).

To further confirm the age‐associated enrichment of the RAB13^+^ arterial‐like EC subset, we performed immunofluorescence (IF) staining for RAB13 in newly formalin‐fixed, paraffin‐embedded (FFPE) BM biopsies from young and elderly donors (Table S5). Notably, RAB13 expression was predominantly localized to periarteriolar regions, as evidenced by the proximity of RAB13^+^ CD31^+^ EC to α‐SMA1^+^ cells, consistent with our transcriptomic findings (Figure S5A). In addition, IF staining for CD31 and RAB13 together with the ribosomal marker pS6 and the TF ZEB1, as well as ZEB1 with α‐SMA1^+^ cells, revealed increased expression of pS6 and ZEB1 in aged EC, consistent with age‐associated changes in ribosomal and translation‐related processes, as well as EMT‐related programs (Figure S5B,C).

Altogether, our findings suggest that aging in human BM is associated with increased inflammation, prothrombotic activity, and the emergence of a distinct EC subset linked to protein synthesis, indicating vascular remodeling with age.

### 2.3 Aged Sinusoids Exhibited Mitochondrial Dysfunction and Impaired Vascular Functions

**FIGURE 3:**
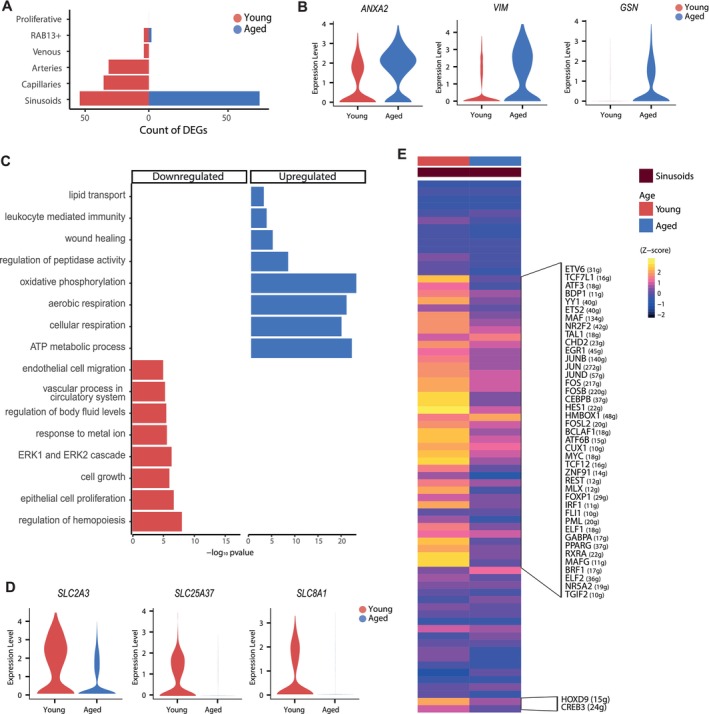
Aged sinusoids exhibited mitochondrial dysfunction and impaired vascular functions. (A) Plot showing the number of age‐related DEGs detected across vascular cell states. (B) Violin plots of expression of *ANXA2*, *VIM*, and *GSN* in young and aged sinusoids. (C) Bar charts of enriched GO terms from ORA (*p* < 0.05) comparing DEGs within young and aged sinusoids. The horizontal axis represents the −log10 of *p*‐values. (D) Violin plots of expression of downregulated solute carriers in aged sinusoids. (E) Heatmap of mean regulon activity score of significantly enriched regulons in each age group within sinusoids. The color scale represents the z‐score of the activity scaled by row.

In addition to examining changes in the composition of EC subtypes with aging, we aimed to investigate the transcriptional alterations in the human BM endothelium associated with aging (Table S6). Notably, sinusoidal EC not only decreased in abundance with age but also exhibited the highest number of differentially expressed genes (DEGs), suggesting a pronounced age‐related dysfunction in this vascular subtype (Figure 3A, Figure S6A). Differential expression analysis identified 88 upregulated and 55 downregulated genes in aged sinusoids (Figure S6B). EC exhibited age‐related changes like those reported in other tissues. Some of these alterations were linked to a procoagulant state, which may be associated with an increased risk of thrombosis in older individuals (Akrivou et al. 2022). The upregulation of genes such as *ANXA2*, *VWF*, and *PLAT*, among others, in aged sinusoids further supported the hypothesis that age‐related endothelial dysfunction may contribute to the heightened incidence of thrombosis (Figure 3B, Figure S6C). In addition to prothrombotic genes, we also observed elevated expression of *VIM, GSN*, and *ANXA5* (Figure 3B, Figure S6C). These genes are crucial in ECM rearrangement by modulating cytoskeletal dynamics and cell‐ECM interactions, especially in pathologies like cancer invasion, fibrosis, and tissue remodeling. Our findings align with previous studies that identify these genes as potential biomarkers of aging across various cell types and tissues (Chen et al. 2020; Zhang et al. 2021). Additionally, aged sinusoids showed upregulation of several genes involved in mitochondrial function and cellular detoxification, such as *ATP5MC2*, *ATP5F1E*, *UQCR11*, *COX4I1*, *COX6A1*, *COX7C*, *NDUFB11*, and *NDUFA11*, reflecting enhanced aging‐related dysfunction in cellular respiration (Figure 3C, Figure S6D and Table S7). Pathway enrichment analysis further revealed that many of these upregulated genes were associated with key hallmarks of aging, including oxidative stress and adipogenesis (Figure S6E).

In contrast, genes involved in cell proliferation and the regulation of EC migration were downregulated in aged sinusoids, which may be related to the increased vascular permeability and leakiness observed in the aged BM (Stucker et al. 2020) (Figure 3C, Figure S6F). Interestingly, we also observed downregulation of some solute carriers (*SLC2A3*, *SLC25A37*, *SLC8A1*), which may reflect a diminished cellular response to metal ions (Figure 3D). This decrease also suggested a diminished capacity of EC to process iron and calcium, potentially contributing to the accumulation of non‐organic compounds observed during aging, ultimately leading to cellular damage (Tian et al. 2022).

Regulon‐based Gene Regulatory Network (GRN) analysis—a TF centered network linking regulators to targets—revealed a global decline in regulon activity in sinusoids along the aging process (Figure 3E). This decline involves TF such as *CEBPB*, *HES1*, *HMBOX1*, *TGIF2*, and *NR5A2*, which are implicated in various biological processes, including cellular proliferation, vascular development, and ion regulation, among others. Collectively, these results point to aging‐associated molecular changes in human BM EC, including signatures related to thrombosis, mitochondrial dysfunction, altered vascular function, and impaired solute transport, which may reflect features of aged sinusoidal endothelium.

### 2.4 THY1 ^+^‐Fibro MSC and Matrix Remodeling Are Key Factors During BM Stromal Aging

**FIGURE 4:**
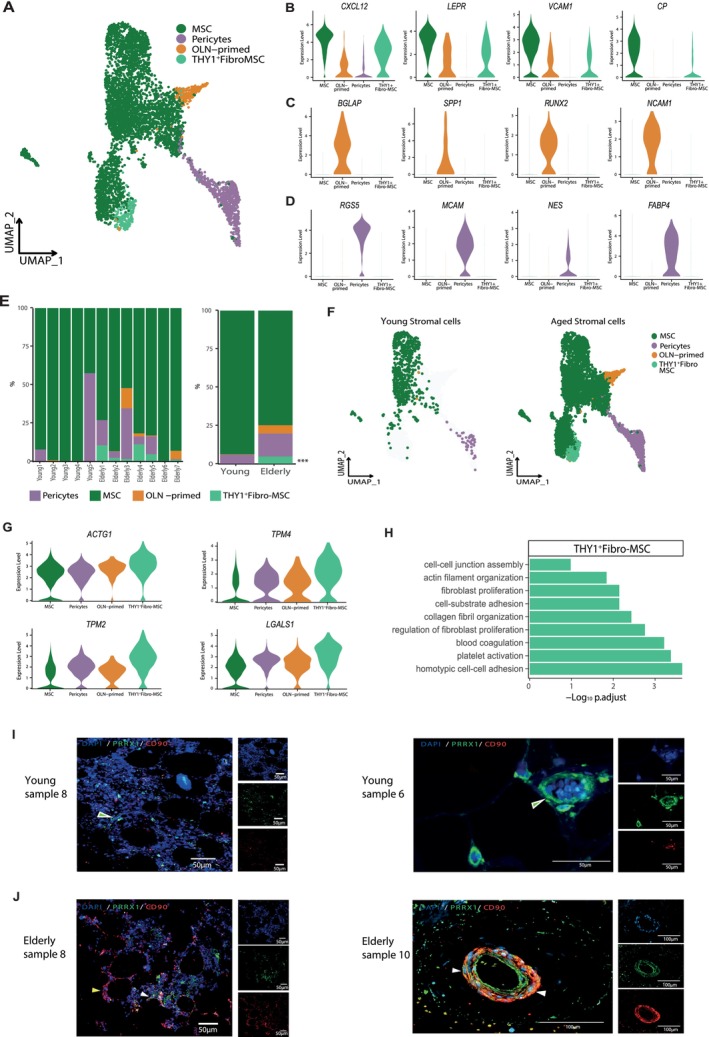
THY1^+^‐Fibro MSC and matrix remodeling are key factors during BM stromal aging. (A) UMAP projection of the stromal cell subtypes within the human BM mesenchymal compartment. (B–D) Violin plots showing the expression of well‐known markers for MSC (B), OLN‐primed (C), and pericytes (D). (E) Stacked bar plots showing the proportions of the different stromal subtypes states in young and aged MSC per sample (left) and age group (right). Asterisks indicate significant differences in the cell proportions between the two groups. (F) UMAP visualization of the stromal subtypes in young (left) and aged (right) MSC. (G) Violin plot showing the expression of matrix‐related genes upregulated in the THY1^+^ Fibro‐MSC cluster. (H) Bar chart of enriched GO terms from ORA (*p* < 0.05) defining the THY1^+^ Fibro‐MSC cluster. The horizontal axis represents the −log10 of adjusted *p*‐values. (I, J) IF staining of THY1^+^ Fibro stromal cells (CD90^+^) (red), MSC (PRRX1^+^) (green), and nucleus (DAPI) (blue) in FFPE biopsies (Table S5) from two young (I) and two elderly (J) individuals. Scale bars are indicated on each panel. Green arrows indicate MSC PRRX1^+^, white arrows indicate MSC THY1^+^ (PRRX1^+^ THY1^+^), and yellow arrows indicate THY1^+^ cells.

To explore the age‐related transcriptional remodeling of the human BM stroma, we first sought to robustly identify well‐established MSC populations in our data based on the expression of established stromal markers (Ye et al. 2022; Bandyopadhyay et al. 2024) (Figure 4A and Table S8). Undifferentiated MSC were defined by the expression of *CXCL12*, *LEPR*, *VCAM1*, and *CP* (Figure 4B). MSC already committed to OLN exhibited elevated expression of osteogenic markers, including *BGLAP*, *SPP1*, *RUNX2*, and *NCAM1*, while the expression of *RGS5*, *MCAM*, *NES*, and *FABP4* distinguished pericytes (Figure 4C,D). We also identified a population of stromal cells highly expressing *LGALS1*, *THY1*, *TIMP1*, and *SPARC* that resembles the Fibro and THY1^+^ MSC subsets recently found in a human BM (Bandyopadhyay et al. 2024) (Figure S7A,B). Consequently, we termed this population THY1^+^ Fibro‐MSC. Despite not capturing a distinctive subset of APOD^+^GSN^high^ MSC as described by Bandyopadhyay et al. (2024), we did observe rare APOD‐expressing cells (Figure S7C). In addition, within the undifferentiated MSC, we also identified two clusters expressing *APOB, PPARG*, *CES1, CEBPA*, and *LPL* that may resemble the previously defined adipo‐lineage subset (Bandyopadhyay et al. 2024) (Figure S7D,E).

To assess the impact of aging on the stromal compartment at the cellular level, we investigated potential changes in the abundance of each cell type between young and aged MSC. Our analysis revealed robust and significant variation in cellular composition among individuals (Figure 4E,F, Figure S7F). Notably, the presence of pericytes and OLN‐primed cells was observed only in specific samples, which may not accurately reflect age‐related changes in proportions, possibly due to variations in sample dissociation. Nevertheless, we consistently found THY1^+^ Fibro‐MSC in all samples from elderly individuals, suggesting that these cells emerge in the aged BM (OR = 1.11, 95% CI: 1.03–1.19, *p* = 0.012) (Figure 4E, left panel). The expansion of these THY1^+^ Fibro‐MSC during aging aligns with the identification of this mesenchymal subset by Bandyopadhyay et al. (2024), whose cohort comprises elderly individuals, thereby approximating a relatively “aged” BM microenvironment.

To delve into the potential role of THY1^+^ Fibro‐MSC as regulators of BM decline during the aging process, we analyzed their transcriptome (Table S9). We observed increased expression of *FN1*, *MIF*, *S100A6*, and *ANXA2*, suggesting the profibrotic nature of aged MSC (Figure S7G). Besides the expression of multiple genes implicated in ECM organization and cell‐matrix interactions, such as *ACTG1*, *TPM2*, *TPM4*, and *LGALS1* (Figure 4G), THY1^+^ Fibro‐MSC showed elevated expression of *COL1A1*, *COL3A1*, and *COL1A2*, which are involved in the TGF‐β signaling pathway (Figure S7H). Interestingly, TGF‐β signaling plays a pivotal role in myelofibrosis by promoting BM fibrosis and collagen deposition (Ghosh et al. 2023). Moreover, these cells exhibited high expression of key genes implicated in myelofibrosis and other fibrotic disorders, such as *IGFBP6* and *IGFBP7* (Figure S7I) (Longhitano et al. 2021). In line with these results, pathway analysis revealed enrichment in fibroblast proliferation, ECM organization, and EMT pathways, suggesting the implication of this profibrotic state in aged MSC (Figure 4H, Figure S7J and Table S9). THY1^+^ Fibro‐MSC also presented enrichment in homophilic cell adhesion, suggesting an altered interaction with the marrow environment (Figure 4H).

To further validate the changes in MSC between young and elderly BM samples, including the identification of THY1^+^ Fibro stromal cells in aged individuals, we performed IF staining of this population in new FFPE young and elderly BM biopsies (Table S5). An increase in THY1^+^ cells among aged MSC, identified by expression of CD90^+^ in PRRX1^+^ cells, was observed (Figure 4I,J). In addition, we confirmed the involvement of this THY1^+^ fibro‐MSC population in ECM remodeling, fibrosis, and TGF‐β signaling by IF staining for fibronectin, type I collagen, and phosphorylated SMAD2 (pSMAD2), respectively (Figure S8A–C). In brief, these results indicate that the aged BM microenvironment may exhibit increased fibrotic features and reduced functional capacity, which could be associated with diminished support for hematopoiesis.

Building on these findings, we hypothesize that THY1^+^ MSC may contribute to the age‐dependent development of fibrosis and may have important implications for BM fibrotic diseases such as myelofibrosis. Using IF staining, we observed an increase in THY1^+^ MSC, marked by an increase in the number of cells co‐expressing CD90 and PRRX1 in the BM of myelofibrosis patients, particularly in scar tissue areas (Figure S8D–F and Table S5). Taken together, the elevated presence of these profibrotic cells in myelofibrosis patients suggests a possible role in the replacement of hematopoietic cells with fibrotic tissue. While further functional validation is needed, these findings imply that such cells may contribute to age‐associated changes in the BM microenvironment and hematopoiesis.

### 2.5 Impaired Differentiation and Oxidative Metabolism in Aged MSC

**FIGURE 5:**
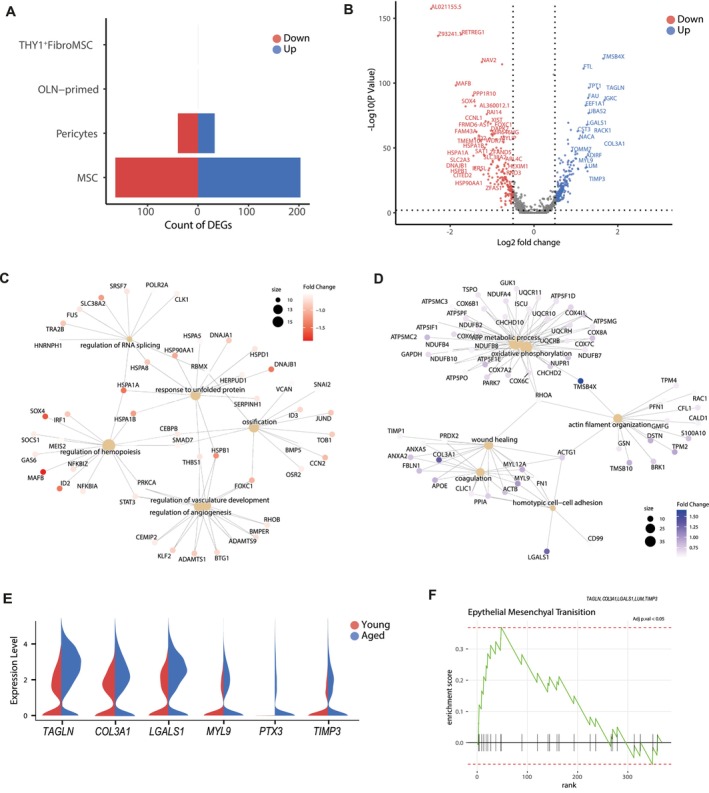
Impaired differentiation and oxidative metabolism in aged MSC. (A) Plot showing the number of age‐related DEGs detected across the BM stromal subpopulation. (B) Volcano plot of the DEGs between young and aged MSC. The y‐axis represents the −log10 (*p*‐value), and the x‐axis represents the log_2_ Fold change. The color of the dot denotes the age group for which DEG was detected, with gray dots representing non‐significant genes. (C) Cnetplot showing the links between genes and biological processes downregulated in aged MSC. Node size reflects the number of significantly enriched genes in the node and colors the log_2_ Fold change expression of each gene. (D) Cnetplot showing the relationship among individual GO terms and genes upregulated in aged MSC. Node size indicates the number of significantly enriched genes in the node and colors the log_2_ Fold change expression of each gene. (E) Split violin plots showing the expression of aging‐related matrix remodeling‐associated genes upregulated in aged MSC. (F) GSEA plot of “Epithelial Mesenchymal Transition” term significantly enriched in aged MSC.

To further delve into the different molecular processes underlying stromal aging, we investigated the transcriptional changes between young and aged stromal cells (Table S10). Undifferentiated MSC showed the largest number of DEGs, demonstrating age‐related defects in these mesenchymal progenitors (Figure 5A, Figure S9A). Specifically, differential expression analysis detected 367 DEGs, including 203 upregulated genes and 164 downregulated genes in aged MSC (Figure 5B). Enrichment analysis revealed that many downregulated genes were linked to ossification‐related processes, suggesting that age‐related bone loss may stem from impairments in the early stages of differentiation (Figure 5C and Table S11). We also found a downregulation of genes that regulate hematopoiesis and cell differentiation, such as *MEIS2*, *GAS6*, *SMAD7*, and *ID2*, as well as heat‐shock proteins and other essential genes mediating the response to protein folding and external stimulus (*HSPA1A*, *HSPA1B*, *NEAT1* and *THBS1* among others) (Figure S9B,C). Moreover, consistent with previous findings in other tissues, the downregulation of angiogenesis‐related terms in aged BM MSC suggests a compromised ability of the mesenchymal progenitors to support BM vascular networks (Duscher et al. 2014).

Additionally, aged MSC showed increased expression of genes associated with OXPHOS and ATP metabolism, including *GSTP1*, *ATP5F1E*, *COX7C*, *NUPR1*, *NDUFA4*, and *CHCHD2*, suggesting an abnormal mitochondrial metabolism in aged MSC (Figure 5D, Figure S9D,E). This could be attributed to mitochondrial dysfunction and elevated ROS levels, in line with the reported impairment of mitochondrial complexes in aged MSC (Zhang et al. 2020). The expression of the adipogenic genes *APOE*, *SPARCL1*, and *CAVIN1*, and several genes involved in ECM organization and remodeling, such as *TAGLN, COL3A1, LGALS1*, *MYL9*, *PTX3*, and *TIMP3*, was increased with age (Table S10 and Figure 5E). Accordingly, Gene Set Enrichment Analysis (GSEA) revealed enrichment in EMT, OXPHOS, and adipogenesis (Figure 5F, Figure S9F). Along with the enhanced EMT process during aging, aged MSC showed decreased expression of metalloproteinases *ADAMTS9* and *ADAMTSL3*, further indicating the role of ECM remodeling during BM stromal aging (Figure S9G). This is aligned with the fact that MSC senescence induces changes in ECM, affecting their repair tissue ability (Ghosh et al. 2020). In summary, our results suggest the contribution of MSC to the age‐related alterations in hematopoiesis. The observation that MSC from aged subjects show an increased oxidative metabolism, EMT, impaired hematopoietic differentiation, and protein folding capacity, along with the presence of THY1^+^ Fibro‐MSC, supports this hypothesis and highlights the BM niche as a target for anti‐aging interventions.

### 2.6 Age‐Related Remodeling of EC‐MSC Communication

**FIGURE 6:**
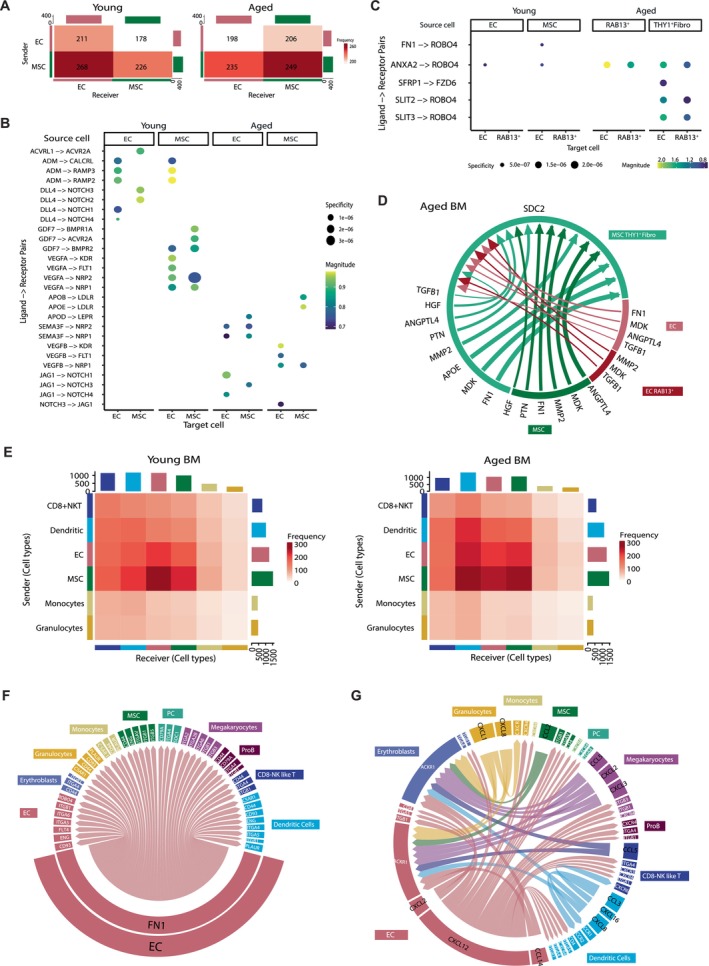
Age‐related remodeling of EC‐MSC communication and the BM interactome. (A) Heatmap illustrates predicted cellular communication between EC and MSC in young and aged BM. (B) Dot plots representing specific L‐R interactions between EC and MSC in young and elderly donors. Dot size and color indicate the specificity and the strength of the interaction, respectively. (C) Dot plots representing L‐R communication pairs involving the RAB13^+^ EC state and THY1^+^ Fibro‐MSC population in young and aged BM. Dot size and color indicate the specificity and the strength of the interaction, respectively. (D) Chord diagram depicting age‐specific communication of THY1^+^ Fibro‐MSC population through *SDC2* receptor. Color represents the signal sender, and width is the strength of interactions. (E) Heatmap illustrates predicted cellular interactions between BM microenvironment cells (EC, MSC, CD8^+^ NKT cells, dendritic cells, monocytes, and granulocytes) in the young (left) and aged (right) BM. Block sizes and red coloration are proportional to the number of L‐R pairs. (F) Chord diagram depicting age‐specific interactions between *FN1* from EC and several proteins from different cell types. Color represents the signal sender, and width is the strength of interactions. (G) Chord diagram showing the significant age‐related signaling network between CXCL and CCL family members and other proteins from various cell types. Colors and widths represent the signal senders and the strength of interactions, respectively.

After characterizing the aging‐associated changes in the non‐hematopoietic BM microenvironment, we aimed to dissect how these alterations may affect cell–cell communication mechanisms. To this end, we conducted a single‐cell‐based ligand‐receptor (L‐R) analysis using LIANA (Dimitrov et al. 2022), focusing initially on biologically informed age‐related changes in the interactions between EC and MSC (Figure 6A and Table S12).

The remodeling of EC and MSC interactions supported the vascular weakening observed in the BM with aging. Specifically, *DLL4*, a Notch ligand crucial for angiogenesis (Akil et al. 2021), interacted with *NOTCH1‐4* in young EC and MSC. In contrast, in aged EC, *DLL4* interactions were replaced by *JAG1*, suggesting an impairment in blood vessel formation (Figure 6B). This observation was further supported by age‐related vascular endothelial growth factor (VEGF) family alterations. In young BM, *VEGFA* mediated interactions with endothelial receptors *KDR*, *FLT1, NRP1*, and *NRP2*, whereas *VEGFB* was predominant in aged individuals (Figure 6B). Additionally, interactions mediated by *GDF7* and bone morphogenetic protein (BMP) elements, presented in the young stroma, were notably absent in aged cells (Figure 6B). In contrast, the aged BM microenvironment showed a more adipogenic phenotype as evidenced by the interactions involving apolipoproteins (*APOB* and *APOE* with *LDLR* in MSC and *APOD* from EC with *LEPR* in MSC) (Figure 6B). These changes in EC‐MSC communication in the aged BM are consistent with the observed shift in MSC differentiation towards adipocytes at the expense of osteoblast differentiation.

A more detailed analysis of the interactions between the RAB13^+^ arterial‐like EC and the THY1^+^ Fibro‐MSC populations identified specific L–R pairs that became more prominent in the aged BM (Figure 6C). Interactions like *SLIT2*‐*ROBO4*, *SLIT3*‐*ROBO4*, and *SFRP1*‐*FZD6* were only identified in aged individuals. The expression of *SFRP1* and *FZD6* has been described as increasing in aged senescent cells, suggesting their potential as markers of Wnt signaling and senescence‐related aging in the BM (Brunt et al. 2012; Donega et al. 2022). Additionally, aged THY1^+^ Fibro‐MSC exhibited extensive crosstalk with both EC and MSC subpopulations through *SDC2* receptor interactions, implicating cell adhesion and ECM processes in the aging BM microenvironment (Figure 6D). Overall, our findings highlight key L‐R interactions that may contribute to the rewiring of aged EC and MSC, uncovering key molecular pathways involved in BM aging.

### 2.7 Impact of Aging on the BM Microenvironment Interactome

Next, we inferred the cell–cell interactions between EC, MSC, and the broader BM microenvironment during aging (Figure S10A,B). Interestingly, interactions with DC increased with age, possibly influenced by the chronic inflammation commonly observed in elderly individuals (Agrawal et al. 2017) (Figure 6E). By examining the age‐related changes on specific ligands and receptors, *FN1* appeared as a putative interactor of the aged BM microenvironment (Figure 6F). *FN1* ligand expressed by MSC, DC, and mainly EC interacts with different members of the integrins family further implicating ECM remodeling in BM aging. Furthermore, predicted *TGFB1*‐mediated interactions increased in aged EC and MSC compared to the young BM (Figure S10C). The interactions of *TGFB1* with its receptors and several integrins, such as *ITGAV* on MSC, which were specific to the aged BM microenvironment, suggest a possible contribution to age‐related fibrosis.

The elevated inflammatory cytokine signaling observed is consistent with the pro‐inflammatory state previously described in aged BM (Figure 6G). *CXCL12* from aged EC interacted with EC, MSC, and DC through *CD4*. Additionally, *ACKR1* from EC and erythroblasts interacted with various hematopoietic cell types via CXCL family members (*CXCL8*, *CXCL2*, *CXCL1*) and CCL chemokines (*CCL14*). This shift towards a pro‐inflammatory microenvironment in aging may be further influenced by increased L–R interactions involving *MIF*, *CD74*, and *CD99*, as observed in the aged BM (Figure S10D).

In conclusion, the remodeling of the BM interactome during aging suggests that non‐hematopoietic components, particularly through increased communication with DC, may contribute to the pro‐inflammatory microenvironment observed in aged individuals. These results are consistent with previous observations in aged mouse stroma, where an inflammatory microenvironment has been shown to alter BM hematopoiesis potentially (Helbling et al. 2019; Chen et al. 2024). Additionally, the *FN1* and *TGFB1* pathways warrant further investigation as potential targets for mitigating fibrotic processes in the aging BM.

### 2.8 Spatial Transcriptomics Supports Age‐Associated Vascular Changes

We have characterized the age‐related shifts in BM non‐hematopoietic cell proportions, molecular profiles, and cell‐to‐cell communication. To gain additional evidence at spatial resolution of these changes, we employed spatial transcriptomics using FFPE BM from two new samples, one young (34 years of age) and one elderly (76 years of age), using the Visium Gene Expression platform (10× Genomics) (Ståhl et al. 2016) (Table S5). After pre‐processing and quality control analysis, we detected 757 and 810 spots in young and elderly samples, respectively, with an average of 612 features and 1417 counts per spot in the young and 561 features and 1415 counts in the elderly (data not shown).

**FIGURE 7:**
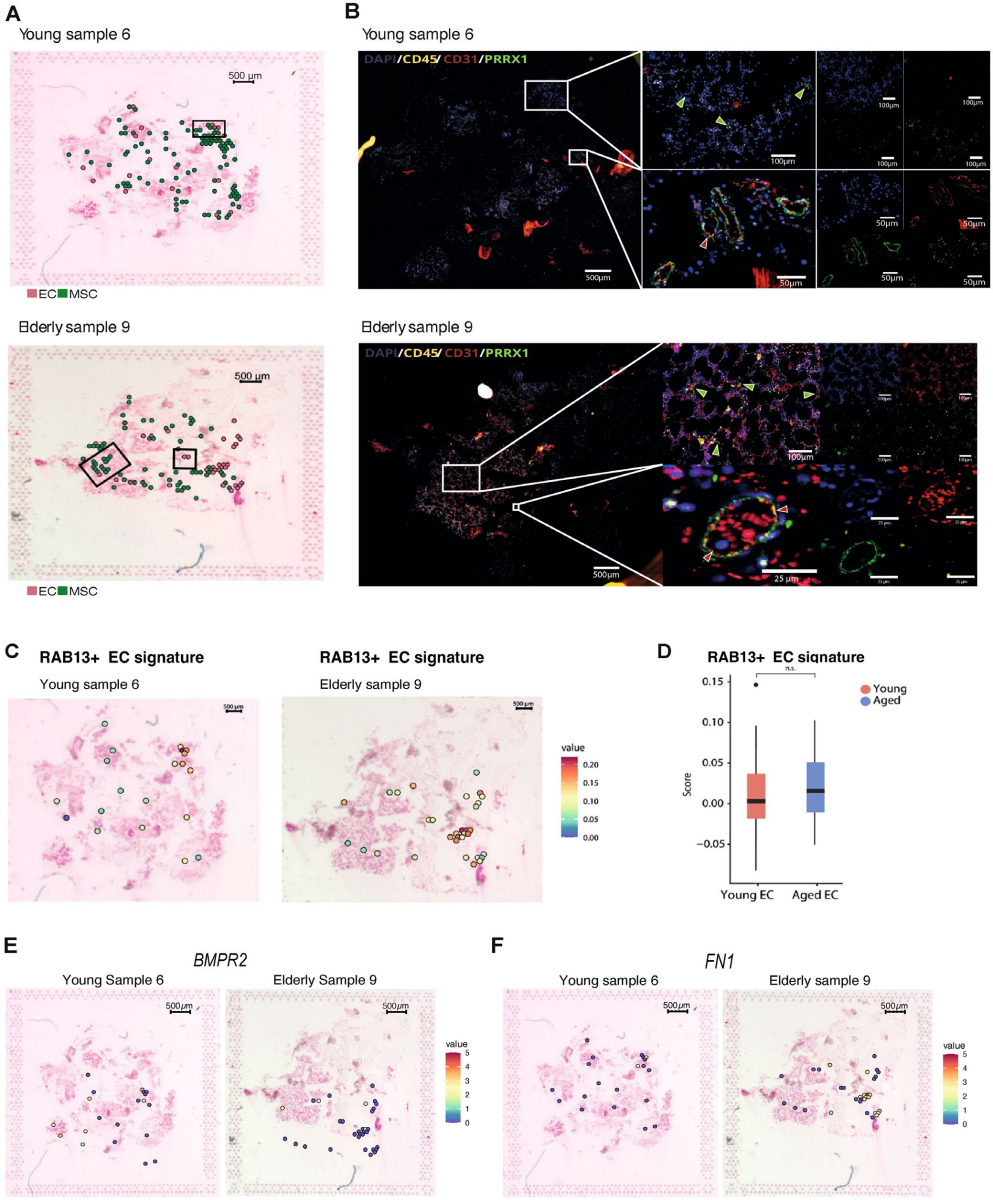
Spatial transcriptomics supports age‐associated vascular changes. (A) Pie charts on H&E staining illustrate the proportion of each cell type highly contributing to the transcriptomic signature of each spot in young sample 6 (upper) and elderly sample 9 (bottom) new BM samples (Table S5). (B) IF staining of hematopoietic cells (CD45^+^) (yellow), EC (CD31^+^) (red), MSC (PRRX1^+^) (green), and nucleus (DAPI) (blue), in young (upper panels) and elderly (bottom panels) BM samples. Scale bars are indicated on each panel. Red arrows indicate the presence of EC (CD31^+^), and green arrows indicate the presence of MSC (PRRX1^+^). (C) Spatial expression patterns of the signature from RAB13^+^ EC identified in scRNA‐seq data in EC‐labeled spots of young (left panel) and elderly (right panel) BM samples. (D) Box plots displaying the RAB13^+^ EC signature in young (red) and elderly (blue) EC. (E) Spatial expression patterns of the BMPR2 gene in EC golden spots of young (left panels) and elderly (right panels) BM samples. (F) Spatial expression patterns of the FN1 ligand in EC golden spots of young (left panels) and elderly (right panels) BM samples.

Given our interest in characterizing aging‐related shifts in EC and MSC, we first aimed to identify the spots with a higher likelihood of containing these specific cell types. To label the spots, we leveraged a scRNA‐seq atlas from Bandyopadhyay et al. (2024), which also includes HSC, as a reference for the annotation analysis. First, we estimated both cell proportion and a signature score per cell type per spot (Figure S11A–C). Then, for each cell type, spots highly ranked for both measures were considered “high‐confidence spots” for such cell type as detailed in Supporting Information methods (Figure S11D–F). All analyses were conducted separately for both samples, resulting in a subset of spots that mapped EC, MSC, as well as hematopoietic cells, including HSC, B cells, DC, erythroblasts, monocytes, neutrophils, PC, and T cells in the human BM (Figure 7A and data not shown). The spatial spots of endothelial and stromal cell populations were confirmed using IF and Hematoxylin–Eosin (H&E) staining (Figure 7B,S,1,1G). Specifically, non‐hematopoietic niche cells were localized around blood vessels and identified by IF using the colocalization of specific markers for EC (CD31^+^ CD45^−^) and MSC (PRRX1^+^ CD45^−^).

Next, we examined the spatial expression patterns of age‐related transcriptional changes identified in our scRNA‐seq analysis, with a focus on EC. Given the limited number of spatial samples (*n* = 1), we lack sufficient statistical power; however, our findings offer an illustrative, spatially resolved support for cellular subtypes and molecular programs identified in the single‐cell dataset. We observed higher values of the RAB13‐related signature in aged EC spots (Figure 7C,D). Moreover, RAB13^+^ EC colocalized with the spatial expression pattern of *YBX1*, a TF involved in RNA splicing and translation control, which was shown to be upregulated in the RAB13^+^ arterial subset identified in our scRNAseq‐data (Figure S12A).

Consistent with the significant age‐related increase in the venous cluster and supporting the “inflammation and immune” hypothesis from the transcriptomic data, we detected a tendency for an increment in spatial expression of *CLU* and *CD74* in aged EC, though the increase was not statistically significant (Figure S12B). Additionally, aged EC‐labeled spots showed a tendency towards oxidative metabolism, which aligns with the mitochondrial dysfunction identified in aged sinusoids through our scRNA‐seq analysis (Figure S12C). *VIM* and *GSN*, two genes implicated in ECM rearrangement and previously found upregulated in aged sinusoids, were highly expressed in aged EC (Figure S12D).

Finally, spatial resolution analysis of L‐R interactions supported findings from our previous BM microenvironment cell‐to‐cell communication analysis. Specifically, we observed reduced *BMPR2* expression in spots enriched in aged EC, indicating diminished support for osteogenic differentiation through EC‐MSC interactions in the aged BM (Figure 7E, Figure S12E). Conversely, *JAG1‐NOTCH3* signaling was detected in spots enriched in aged EC (Figure S12F). In line with the increased interactions between niche cells and DC identified by our scRNA‐seq analysis, EC‐labeled spots in aged BM showed a positive correlation with the DC score, suggesting a role for EC‐DC communication in the pro‐inflammatory aged BM (Figure S12G). Interestingly, we observed a higher spatial expression of the *FN1* ligand in spots enriched in aged EC, supporting our previous transcriptomic findings pointing FN1 as a key interactor in the aged BM (Figure 7F, Figure S12H).

Overall, our findings showed spatially distinct, age‐related changes within the human non‐hematopoietic BM microenvironment, with an impact on EC. Supported by our scRNA‐seq analysis, these results suggest alterations in cell state distribution, signaling pathways, and intercellular communication within the aged BM.

## 3 Discussion

Over the past decades, significant progress has been made in elucidating the mechanisms of human BM aging. Advances in scRNA‐seq have provided unprecedented resolution into the heterogeneity and age‐related trajectories of HSC (Su et al. 2024; Ainciburu et al. 2023; López‐Otín et al. 2023). Parallel studies of the aged HSC niche have uncovered widespread alterations in the BM microenvironment that actively contribute to the functional decline of HSC over time (López‐Otín et al. 2023; Guidi et al. 2021, 2017; Kwon et al. 2024; Feng et al. 2024; Gustafsson and Scadden 2017). These findings have underscored the BM niche as a promising target for therapeutic rejuvenation strategies. Nonetheless, due to the inherent challenges of studying aging in humans, much of our current knowledge stems from murine models (Ramalingam et al. 2023; Ho et al. 2019; Yuan et al. 2023).

Here, we provide a comprehensive transcriptional characterization of the changes that occur during physiological aging of the BM non‐hematopoietic microenvironment with a special focus on the endothelial and mesenchymal compartments. Additionally, using IF staining and spatial transcriptomic profiling, we provide valuable preliminary insights into the spatial organization of these cells. Most significantly, we provide support for some of the findings derived from the scRNA‐seq analysis of BM aging.

Our findings expand on the well‐characterized arteries and sinusoids, uncovering previously undescribed vascular subtypes within the human BM endothelium, including capillaries and veins. We revealed significant age‐dependent shifts in EC composition, with an increased prevalence of the venous cluster in elderly samples, marked by genes related to immune activation and coagulation. This indicates a shift towards enhanced inflammation and elevated thrombotic activity with aging in EC. Interestingly, we uncovered an arterial‐related cluster in aged EC, characterized by the high expression of *RAB13* associated with dysregulation of translational elongation activity and ribosomal processes. These results are consistent with the previously reported upregulation of gene sets related to translation, post‐transcriptional regulation, and RNA and protein metabolism in EC from aged mice (Helbling et al. 2019; Chen et al. 2024). The spatial enrichment of this arterial‐associated EC subset during aging was further demonstrated by IF staining, showing the colocalization of RAB13^+^ cells with CD31^+^ EC and α‐SMA1^+^ cells. This newly identified aging‐related subset, revealed through scRNA‐seq and spatial transcriptomics, is potentially regulated by the TF ZEB1 (Yu et al. 2022) and demonstrates elevated expression of RBP, positioning these cells as candidate regulators of aging and promoters of cellular senescence (Varesi et al. 2023; Han and Kim 2022). The role of RAB13 in cancer progression is well‐documented, making the identification of RAB13^+^ cells as a novel subset of aged EC a potential therapeutic target (Wang et al. 2022). Sinusoids were identified as the most distinct vascular bed during aging, exhibiting a prothrombotic profile, mitochondrial dysfunction, and a decline in transcriptional activity. Aged sinusoids also showed a signature associated with an impaired ability to maintain vascular integrity and angiogenic potential, possibly promoting regression in vascular structure and disrupting inflammatory homeostasis, consistent with previous studies conducted in mice (Ho and Méndez‐Ferrer 2020; Ho et al. 2019; Winter et al. 2024).

Even with considerable advancements in characterizing the human BM stroma, age‐related transcriptional changes in MSC remain unresolved (Li et al. 2023; Bandyopadhyay et al. 2024; de Jong et al. 2021; Gao et al. 2023). Notably, our results revealed the emergence of THY1^+^ Fibro‐MSC in the aged stroma, which exhibited high similarity to the recently identified THY1^+^ Fibro‐MSC subpopulations (Bandyopadhyay et al. 2024). These cells showed an enrichment of genes associated with the TGF‐β signaling pathway, ECM, and EMT, suggesting the implication of these profibrotic processes in aged MSC. Moreover, the transcriptional profile of THY1^+^ fibro‐like MSC shows strong convergence with stromal programs described in murine models of myelofibrosis (Li et al. 2024). Both populations display elevated expression of *LGALS1* (galectin‐1) and *S100A6*, two genes identified as dysregulated mediators of inflammatory–fibrotic crosstalk. We also confirmed histologically that CD90^+^ BM MSC coexist with fibrosis (Dudli et al. 2022). Furthermore, analyzing the transcriptional changes in MSC during aging showed an increase of ATP metabolism and OXPHOS in aged individuals, reduced unfolded protein response, and impaired support for hematopoietic and osteoblast differentiation. Overall, our findings reveal extensive transcriptional remodeling within the endothelial and mesenchymal compartments of the BM, characterized by increased inflammation, impaired ribosomal and mitochondrial function, and elevated ROS production. These age‐specific changes align with established hallmarks of aging (Yang et al. 2023; Maldonado et al. 2023) and previously aged mice results (Helbling et al. 2019; Chen et al. 2024), positioning EC and MSC as promising targets for strategies aimed at rejuvenating the aged BM microenvironment (Barilani et al. 2022; Mistry et al. 2024).

The differences in the BM microenvironment significantly influence the behavior and regenerative capacity of aged HSC (Li et al. 2025). Therefore, studying this crosstalk is crucial for developing therapeutic strategies to mitigate age‐related decline in HSC function and enhance health outcomes in older individuals. The shift from *VEGFA* to *VEGFB* interactions and the altered NOTCH signaling observed in aged EC may contribute to impaired blood vessel formation during aging (Rastogi et al. 2021). This disruption of NOTCH signaling supports previous studies highlighting its role in maintaining HSC stemness (Ho and Méndez‐Ferrer 2020; Kusumbe et al. 2016). Moreover, the aged BM niche showed increased apolipoprotein interactions, consistent with the accumulation of adipocytes during BM aging and the compromised differentiation of aged MSC (Al‐Azab et al. 2022). Remarkably, we identified *SFRP1‐FZD6* and *SDC2* receptor interactions as potential key regulators of senescence and ECM processes related to the emergence of RAB13^+^ EC state and THY1^+^ Fibro‐MSC population during aging. *FN1* and *TGFB1* emerged as central signaling hubs in the aged BM microenvironment, reflecting impaired matrix remodeling and potentially driving cell activation and inflammatory processes (Al‐Yafeai et al. 2018). This shift towards a pro‐inflammatory state during the aging process was reinforced by the enhanced communication with DC and the increased interactions mediated by cytokine signaling, *MIF*, *CD74*, and *CD99*.

Despite the technical limitations associated with applying spatial transcriptomics to mineralized tissues and BM in particular (Xiao et al. 2023; Muiños‐Lopez et al. 2025), we were able to perform Visium Gene Expression technology from 10× Genomics on human FFPE BM biopsies from young and elderly individuals. We mapped the spatial distribution of hematopoietic and non‐hematopoietic components within the human BM niche, which was supported by IF staining for CD45, CD31, and PRRX1. Our data provided initial spatial context for the EC changes previously observed in our scRNA‐seq analysis. Notably, we observed an increased spatial localization of RAB13^+^ senescent cells near periarteriolar regions, alongside distal EC showing elevated expression of *GSN* in the aged BM. Additionally, our spatial transcriptomic data supported the enhanced communication between aged EC and DC, and the FN1 signaling pathway as a potential interaction hub in the aged BM.

In summary, our study provides novel insights into the age‐related remodeling of the EC and MSC transcriptome, interactome, and spatial architecture, offering potential explanations for the deterioration of the hematopoietic system observed during aging. We find that aging compromises the ability of EC to maintain vascular integrity and reduces the protein folding capacity and support for hematopoietic differentiation of MSC. Both cell types exhibit compromised mitochondrial function and heightened inflammatory and prothrombotic properties with age. We also identified an age‐related EC subset responsible for translation and ribosomal processes. Moreover, we observe a more fibrotic and less functional aged BM stroma influenced by THY1^+^ Fibro‐MSC, which may contribute to its diminished capacity to support hematopoiesis in aging‐related pathologies. Our spatial transcriptomics analysis supports our previous scRNA‐seq results, highlighting the presence of RAB13^+^ senescent cells in aged EC and age‐associated interactions. Collectively, our results highlight the crucial role of non‐hematopoietic components in shaping the functional trajectory and longevity of HSC. These age‐related alterations position the non‐hematopoietic BM niche as a promising therapeutic target for mitigating hematopoietic decline and enhancing tissue regeneration in aging populations.

## 4 Materials and Methods

### 4.1 Human Samples

Human BM samples were obtained from BM tissue of healthy young (28–48 years of age) and elderly (58–79 years of age) individuals of both sexes undergoing orthopedic surgery (hip or knee replacement). Samples were collected at Hospital Universitario de Navarra and Hospital Reina Sofía de Tudela. Written informed consent was obtained according to the Declaration of Helsinki and the Institutional Review Board of the University of Navarra. The characteristics of all individuals are described in Table S1.

### 4.2 Human BM Biopsies

Human BM biopsies from young (34–48 years of age) and aged (72–78 years of age) individuals were obtained from Hospital Universitario de Navarra after obtaining written informed consent for IF and spatial analysis (Table S5). Archived paraffined human BM biopsies of four MF patients (65–73 years of age) were obtained from the Pathology Department of Clínica Universidad de Navarra after written informed consent was achieved (Table S5). The Research Ethics Committee of the University of Navarra approved the human sample collection and research conducted in this study. Personal data was kept confidential following the Organic Law 3/2018 on personal data protection and Spanish Law 14/2007 on Biomedical research. All collection samples are codified; only authorized personnel can correlate the patient's identity with the codes.

### 4.3 Isolation and Fluorescence‐Activated Cell Sorting of Endothelial and Mesenchymal BM Cells

Endothelial and mesenchymal human non‐hematopoietic BM cells were isolated and sorted for subsequent scRNA‐seq analysis (Figure S1). All sample processing steps were performed on ice to preserve cell viability and RNA integrity, except red blood cell lysis, which was conducted at room temperature (RT). To isolate endothelial and mesenchymal BM cells, red blood cells were lysed using a 45 mL:5 mL ratio of ACK lysis buffer per human sample, filtered through a 70 μm filter, and then collected into a tube. Bone fragments were manually cut, crushed, and digested with 0.3% collagenase I and dispase (5 U/mL) for 15 min at 37°C and shaken at 200 rpm. After digestion, the collagenized bone fraction was filtered through a 70 μm filter into a collection tube, pooled with BM fraction, and centrifuged at 1500 rpm and 4°C for 5 min. To determine live cell concentration and viability, 10 μL of each sample was stained with acridine orange and propidium iodide (AO/PI) solution (Nexcelom) and analyzed with Cellometer K2 Image Cytometer (Nexcelom Bioscience). Cells were subsequently stained for 30 min on ice in PBS 1× containing 2% FBS and 2 mM EDTA (modPBS) with the following combination of conjugated antibodies: BV510 labeled anti‐Lin (including CD3, CD19, CD45 and CD64), BV421 labeled anti‐CD235, BV421 labeled anti‐CD45, FITC labeled anti‐CD16, PE‐Cy7 labeled anti‐CD56, PE labeled anti‐CD31, APC‐Cy7 labeled anti‐CD9, and PerCP‐Cy5.5 labeled anti‐CD271. All antibodies were added at a concentration of 1/100 except anti‐Lin, where we added 3 μL/test—test 25 × 106 cells, anti‐CD16, 10 μL/test—test 25 × 106 cells and 1/50 anti‐CD31 and anti‐CD56. Samples were resuspended in modPBS with 6 μL of TO‐PRO‐3 for cell sorting. Sorting gates were set according to the corresponding fluorescence‐minus‐one (FMO) controls, and cells were sorted using BD FACSAria II. Dead cells were excluded by TO‐PRO‐3 staining, and doublets and debris were excluded by gating on FSC and SSC. EC and MSC were prospectively isolated based on the following immunophenotype (Figure S1B): TO‐PRO‐3^−^/Lin^−^/CD45^−^/CD235^−^/CD16^−^/CD56^−^/CD9^+^/CD31^+^ for EC and TO‐PRO‐3^−^/Lin^−^/CD45^−^/CD235^−^/CD16^−^/CD56^−^/CD31^−^/CD271^+^ for MSC. EC and MSC were directly sorted into PBS 1× with 0.05% UltraPure for subsequent scRNA‐seq protocol. Cell viability of sorted cells was assessed using Nexcelom Cellometer as described above.

### 4.4 Single‐Cell RNA Sequencing

scRNA‐seq was performed using the Single Cell 3′ Reagent Kits v3.1 (10× Genomics) according to the manufacturer's instructions. Approximately 10,000 cells were loaded at a concentration of 1000 cells/μL on a Chromium Controller instrument (10× Genomics) to generate single‐cell gel bead‐in‐emulsions (GEMs). Each cell was encapsulated with primers containing a fixed Illumina Read one sequence, followed by a cell‐identifying 16 bp 10× barcode, a 10 bp Unique Molecular Identifier (UMI), and a poly‐dT sequence. A subsequent reverse transcription yielded full‐length, barcoded cDNA. This cDNA was then released from the GEMs, PCR‐amplified, and purified with magnetic beads (SPRIselect, Beckman Coulter). Enzymatic Fragmentation and Size Selection optimized cDNA size before library construction. Illumina adaptor sequences were added, and the resulting library was amplified via end repair, A‐tailing, adaptor ligation, and PCR. Libraries' quality control and quantification were performed using Qubit 3.0 Fluorometer (Life Technologies) and Agilent's 4200 TapeStation System (Agilent), respectively. Sequencing was performed in a NextSeq2000 (Illumina) (Read 1: 28 cycles, i7 Index: 10 cycles, i5 Index:10 Read 2: 90 cycles) at an average depth of 45,000 reads/cell followed by computational alignment using CellRanger (version (v) 6.1.1, 10× Genomics) against the human GRCh38 reference genome.

### 4.5 Single‐Cell RNA Sequencing Analysis

The single‐cell analysis of the BM samples was performed using R version (v) 4.1.3 and Seurat v 4.3.0 (Stuart et al. 2019). For data pre‐processing, doublet scores were calculated by scDblFinder v.1.8.0 (Germain et al. 2021). Datasets were filtered individually based on a library complexity of more than 200 features, features detected in more than 3 cells, doublets, and high percentages of mitochondrial genes (> 10%). After individual sample pre‐processing, single‐cell datasets from young and old individuals were merged, normalized using the SCTransform method (Hafemeister and Satija 2019), and analyzed by principal‐component analysis (PCA) (*k* = 30) on the most variable genes (*k* = 3000) across all cells. A batch correction was performed using the RunHarmony function from the Harmony R package (Korsunsky et al. 2019), setting the donor identity as the batch factor. The batch‐corrected coordinate space was used for linear dimensional reduction with Seurat. Unsupervised clustering was performed by computing the K‐nearest neighbors, applying the Louvain algorithm at resolution 0.4, and cells were projected in two dimensions using Uniform Manifold Approximation and Projection (UMAP).

Significantly upregulated genes in each cluster compared to all other clusters (Bonferroni‐adjusted *p* < 0.05) were identified using the Seurat Function FindMarkers following MAST (Model‐based Analysis of Single‐cell Transcriptomics) methodology including donor identity as a covariate (Finak et al. 2015). Cell types were identified by manual cell type annotations according to published canonical marker genes. Despite the inclusion of multiple hematopoietic markers in the exclusion panel, residual hematopoietic cells were captured in our scRNA‐seq dataset. To ensure analytical robustness, all downstream niche analyses were restricted to stringently annotated endothelial and mesenchymal populations based on established canonical markers.

### 4.6 Characterization of Sub‐Cell Types and Functional States in Human BM EC and MSC

To characterize endothelial and mesenchymal BM compartments, clusters identified as EC and MSC were subsetted into separate objects, and unsupervised cell clustering was implemented. The stability of the clusters was evaluated following a bootstrapping strategy, and cells from non‐robust clusters were assigned to the neighboring clusters, repeating a Random‐Forest‐based strategy as described in our previous study (Ye et al. 2022). Additionally, two clusters identified within EC and some outlier cells within MSC, lacking canonical marker genes for their respective population, were excluded from downstream analysis. To identify the markers for each cluster, genes that were differentially expressed (min. pct > 0.25, log fold change > 1, and Bonferroni adjusted *p* < 0.05) in a cluster compared to all the others were identified using the FindAllMarkers function with MAST including donor identity as a covariate. GO ORA was conducted using clusterProfiler to detect enriched canonical pathways (adjusted *p* < 0.05) among the identified cluster markers (Ashburner et al. 2000; Yu et al. 2012). Cell clustering identities were then annotated by cross‐referencing these cluster‐specific genes and functional gene sets with published data.

To further assess the robustness of identified EC and MSC subpopulations, we implemented donor‐balanced downsampling and clustering stability analyses. Specifically, an equal number of cells per donor was randomly sampled and the full dimensionality reduction and clustering workflow was recomputed across 100 independent bootstrap iterations. Clustering concordance across iterations was quantified using Jaccard similarity indices and the Adjusted Rand Index (ARI), comparing recomputed clusters with the original annotations (Figures S13 and S14). Additionally, we performed donor‐level pseudobulk differential expression analyses. For each cell subtype of interest, gene counts were aggregated per donor, and differential expression was assessed using edgeR, treating donors as biological replicates. Concordance between single‐cell and pseudobulk analyses was evaluated by comparing effect sizes (Figure S15).

### 4.7 Age‐Related Differential Expression and Functional Enrichment Analysis

DEGs between young and aged cells were determined with the Seurat FindMarkers function with MAST including donor identity as a covariate (min. pct > 0.25, absolute log fold change > 0.5; and Bonferroni adjusted *p* < 0.05). Significant DEGs for each group were used as input for a GO ORA using clusterProfiler to investigate the biological differences between age groups. GO terms with corrected *p*‐values less than 0.05 were considered significantly enriched. GSEA was also calculated using the GO and Hallmark gene sets from the Molecular Signatures Database v.7.5.1 in the list of DEGs ranked according to log2FC.

To confirm bioinformatically age‐associated transcriptional changes at the donor level, we additionally performed donor‐level pseudobulk differential expression analyses. For each cell subtype of interest, gene counts were aggregated per donor, and differential expression was assessed using edgeR, treating donors as biological replicates. Concordance between single‐cell and pseudobulk analyses was evaluated by comparing effect sizes (Figure S15).

### 4.8 Age‐Related Changes in Cell Proportion

To robustly determine the statistical significance of differences in cell‐type proportions between young and aged conditions we conducted a permutation‐based analysis using the R‐package scProportionTest (Miller et al. 2021). Briefly, this method runs a permutation test on the two conditions for each cell cluster. It returns the relative change in cell type abundance between the two groups with a confidence interval for each comparison. To account for inter‐individual variability in cellular composition and to improve the robustness of confidence interval estimation, we additionally applied a donor‐balanced bootstrapping strategy. In each of 100 iterations, an equal number of cells from each age group was randomly selected. For each cell type in each iteration, the number of cells was determined and divided by the total cell number of the iteration to calculate the cell‐type proportions. Bootstrap sampling was used to generate 95% confidence intervals for plotting. Changes in population sizes with FDR < 0.05 and log2FoldChange > 1 were denoted as statistically significant.

In addition, sensitivity analyses excluding dominant donors were performed for EC and MSC compartments. After removal of individual donors contributing disproportionately to specific clusters, dimensionality reduction, clustering, and cell proportion analyses were recomputed to confirm robustness of age‐associated changes (Figures S16 and S17).

### 4.9 Correlation of Cell Subpopulations With Chronological Age

To further validate age‐related changes in cell‐type proportions, we assess their correlation with chronological age using generalized linear mixed‐effects models (GLMMs) (Table S13). For each cell subpopulation, we modeled the probability of belonging to that subpopulation as a function of age in years, with sample identity included as a random effect (glmer (cell type ~ age + (1|sample ID), family = binomial())).

### 4.10 Gene Regulatory Network Analysis

GRN activity was interrogated following the workflow implemented by SCENIC (Aibar et al. 2017). An equal number of cells per condition and cell type was randomly selected for the analysis. Briefly, all the genes were trained in the GENIE3 package and used to develop a random forest model for selecting co‐expression modules between TFs and target genes (regulons). Regions for TFs searching were restricted to a 10‐k distance centered on the transcriptional start site (TSS) or 500 bp upstream of the TSSs. Then, RcisTarget was used to refine the regulons by inferring direct targets of the TFs. Finally, regulon activity scores were calculated for each cell, using the AUCell package, to determine whether the regulons were in an active or inactive state.

SCENIC GRN results were further validated using SimiC (Peng et al. 2022). For each comparison, 100 TFs and 1000 target genes were selected based on their variability, determined by calculating the maximum absolute deviation. To determine the optimal parameters, each analysis involved a cross‐validation run. After parameter tuning with cross‐validation, we set lambda1 = 0.01, lambda2 = 0.1. We extracted regulons by calculating association weights for each TF and target and filtering out small weights. The resulting GRNs were visualized using the GRN incidence matrices provided by SimiC. Histograms for different regulons were computed using the “regulon activity score” provided by SimiC. We then computed the weighted area under the curve (wAUC) to measure regulon activity per cell. Additionally, this score was utilized to calculate the regulatory dissimilarity score between functional cell states. Lastly, we tested for differences in regulon activity between young and old EC and MSC, performing a Kolmogorov–Smirnov test for the wAUC distributions.

### 4.11 Cell‐To‐Cell Communication Analysis

Cell–cell communication analysis was performed using liana R package v.0.1.13 (Dimitrov et al. 2022), which integrates multiple L–R inference methods and curated interaction resources. EC, MSC, and additional annotated hematopoietic BM populations were used as sender and receiver cell types. L–R interactions were inferred using the OmniPath interaction resource.

To reduce biases arising from unequal cell‐type abundances and to ensure robustness, a bootstrapping strategy was applied separately to young and aged datasets. For each age group, an equal number of cells per cell type was randomly sampled (equal to the minimum cell number across cell types), and this procedure was repeated for 100 bootstrap iterations. (Dimitrov et al. 2022). For each iteration, the subsampled cells were subjected to LIANA analysis using five inference methods (cellphonedb, connectome, NATMI, logFC, and SingleCellSignalR) and results were retained for downstream comparison.

For each bootstrap iteration, inferred L–R interactions were aggregated using LIANA's specificity‐based aggregation framework (liana_aggregate function) generating an aggregate rank score for each interaction, reflecting the specificity of a given ligand–receptor pair for a defined sender–receiver cell‐type pair while integrating evidence across inference methods.

Prioritization of candidate interactions followed a multi‐step filtering strategy:

1. Specificity filtering: interactions were retained if aggregate_rank < 0.01.
2. Bootstrap robustness filtering: for each unique interaction (sender, receiver, ligand complex, receptor complex), the frequency of recovery across bootstrap iterations was quantified; only interactions above the 95th percentile of the bootstrap frequency distribution were retained as robust.
3. Age‐group stratification: prioritized interactions were identified independently in young and aged datasets, enabling classification of interactions as young‐specific, aged‐specific, or shared.
4. Curation‐based confidence filtering: prioritized interactions were annotated using OmniPath metadata (e.g., curation effort, number of references, number of resources), and only curated interactions (curation_effort ≥ 1) were retained as high‐confidence.
  To illustrate the strength of specific interactions between different groups, several L‐R pairs with high mean values were selected for visualization. The complete list of ligand‐receptor interaction pairs is shown in Table S12.

### 4.12 Immunofluorescence and Masson's Trichrome Staining

Bone tissue sections were fixed in formol 4% (PanReac) for 24 h at RT and then decalcified using EDTA 0.25 M pH 6.95 (Invitrogen) for 10 days at RT with agitation. Following decalcification, the samples were washed in distilled water for 5 min and sequentially dehydrated in ethanol at increasing concentrations: 70% for 1 h, 80% for 1 h, 96% for 1 h, and 100% overnight. The samples were then cleared in xylol (PanReac) for 4 h. Fixed bone samples were subsequently embedded in paraffin and incubated at 60°C overnight. Subsequently, 4 μm tissue sections were mounted on microscopy slides and dried in a desiccator at 37°C overnight. The preparations were deparaffinized and rehydrated through a series of graded alcohols. Antigen retrieval was performed by heating the slides in 10 mM Tris‐1 mM EDTA buffer (pH 9) for 30 min at 95°C, except samples for RAB13, EMCN and rabbit monoclonal Phospho‐S6 Ribosomal Protein (pS6) staining that were heated in 10 μM Citrate (pH 6).

For Masson's Trichrome staining, the samples were performed according to standard protocols to determine fibrosis in the human BM niche (Van De Vlekkert et al. 2020). Tissue imaging was performed using an Aperio CS2 Scanner (Leica Biosystems) at 20× magnification. Image analysis was achieved using QuPath (version 0.5.1) to evaluate cell circularity and ImageJ (version 2.14.0) to quantify fiber percentage based on color intensity.

For IF staining, the tissue sections were blocked with BSA 5% for 30 min at RT and the following primary antibodies were used: rabbit anti‐CD45 Alexa Fluor 647 conjugate (Cell Signaling Technology, 1:50 dilution), mouse anti‐CD31 antibody (DakoCytomation, 1:40 dilution), rabbit anti‐PRRX1 antibody (Sigma‐Aldrich, 1:50 dilution), mouse anti‐Alpha smooth muscle actin antibody (Sigma Aldrich 1:1000 dilution), rat anti‐Endomucin antibody (V.7C7) (Santa Cruz Technology 1: 40 dilution), mouse PE anti‐human CD144 (VE‐Cadherin) Antibody (BioLegend 1:50 dilution), mouse Anti‐human RAB13 Antibody (MA5‐31879) (ThermoFisher Scientific 1:100 dilution), rabbit monoclonal Phospho‐S6 Ribosomal Protein (pS6) (Ser235/236) (D57.2.2E) (4858) (Cell Signaling Technology), (1:200 dilution), rabbit polyclonal ZEB1 (21544–1‐AP) (Proteintech), (1:100 dilution), mouse anti‐human CD90 Antibody (MA5‐16671) (ThermoFisher Scientific 1:100 dilution), mouse monoclonal antibody CD271 (NGF Receptor) (ME20.4) (14‐9400‐82) (ThermoFisher 1:50), rabbit anti‐human anti‐Fibronectin antibody (F‐3648) (Sigma, 1:100), rabbit anti‐human Type I collagen (2150‐0020) (AbD Serotec, 1:20), rabbit anti‐mouse anti‐Smad2 antibody, phospho‐specific (Ser465/467) (80427‐2‐RR) (Labclinics, 1:100). The slides were subsequently incubated with the corresponding secondary antibodies in a 1:200 dilution: goat anti‐Rabbit IgG Alexa Fluor 647 (Invitrogen), goat anti‐Mouse Alexa Fluor 568 (Invitrogen), goat anti‐Mouse Alexa Fluor 488 (Invitrogen), and goat anti‐Rat Alexa Fluor 647 (Invitrogen). Nuclei were stained with DAPI (Vectashield 1:50 dilution). Fluorescence images were acquired using the Vectra Polaris Multispectral Imaging System (Perkin Elmer). Image analysis was made using QuPath (0.5.1) to assess the co‐expression of CD90 and PRRX1 in the BM of young, elderly, and MF patients.

### 4.13 Preparation and Sequencing of Spatial Transcriptomics Samples and Libraries

Human bone samples were processed as previously described (Muiños‐Lopez et al. 2025). Bone tissue sections were fixed in formol 4% (PanReac) for 24 h at RT and then decalcified with EDTA 0.25 M pH 6.95 (Invitrogen) for 10 days at RT with agitation. After decalcification, samples were washed in distilled water for 5 min and sequentially dehydrated in ethanol 70% (1 h), 80% (1 h), 96% (1 h), and 100% (overnight), followed by 4 h in xylol (Panreac). Fixed bone samples were subsequently embedded in paraffin and incubated at 60°C overnight.

Prior to spatial transcriptomics experiments, RNA integrity of the FFPE tissue, a critical factor for spatial transcriptomics success, was examined on 10 μm sections. RNA was extracted using RNeasy FFPE kit (Qiagen) and examined with RNA ScreenTape Assay to determine the samples DV200. Next, 5 μm tissue sections were mounted on microscopy slides, baked at 42°C for 3 h, and dried in a desiccator at 37°C overnight. For spatial transcriptomics assays, preparations were deparaffined, rehydrated, and H&E stained following 10× Genomics recommendations. Tissue imaging was performed using an Aperio CS2 Scanner (Leica Biosystems) at 20× magnification. Sections were destained and decrosslinked before library construction using Visium CytAssist Spatial Gene Expression for FFPE Human Transcriptome (10× Genomics). Briefly, a whole transcriptome panel consisting of 3 probe pairs per gene was hybridized with their target RNAs in the tissue sections. Neighboring probe pairs that had hybridized to RNA were then ligated. Tissue slides and Visium CytAssist Spatial Gene Expression v2 Slides were loaded into a Visium CytAssist instrument (10× Genomics). Through RNase treatment and tissue permeabilization, ligated probes were released and readily diffused onto Visium v2 slides containing spatially barcoded oligonucleotides. Probes were spatially labeled through extension, released from the slide, and pooled. Finally, samples were indexed via PCR amplification. The resulting libraries were quantified with Qubit dsDNA HS Assay Kit, and their profile was examined using Agilent's HS D1000 ScreenTape Assay. Sequencing was carried out in an Illumina NextSeq2000 using paired‐end, dual‐index sequencing (Rd1: 28 cycles; i7: 10 cycles; i5: 10 cycles; Rd2: 50 cycles) at a minimum depth of 25,000 reads per spot.

### 4.14 Pre‐Processing and QC Spatial Transcriptomics Data

Spatial transcriptomic data were demultiplexed and mapped using the SpaceRanger software (v2.0.1). The Ensembl 105 genomes were used as reference (GRCh38). Filtered feature‐barcode expression matrices obtained from SpaceRanger were used as initial input for the spatial transcriptomics analysis using Seurat (v4.3.0.1) and Stutility (v1.1.1). Spots overlapping the bone tissue were manually removed based on the H&E staining images using the Cloupe (v6.0.0). Spots with less than 200 UMI and 150 features were filtered out. Immunoglobulin genes and those genes that were not present in both samples were filtered out.

### 4.15 Deconvolution Analysis

We downloaded a pre‐processed human single‐cell dataset published by Bandyopadhyay et al. (2024) for deconvolution analysis. We removed or relabeled their cell type annotation to obtain our interest's 10 major cell types (B‐cells, T‐cells, Neutrophils, Erythroblasts, Monocytes, DC, PC, MSC, EC, and HSC). Deconvolution analysis used the relabeled single‐cell reference with CARD (v1.0.0) with default parameters.

### 4.16 Spot Labeling

The estimated cell‐type percentage values derived from the deconvolution were used to define the percentage of each cell type in each sample. We used the deconvolution estimation and the area under the curve (AUC) calculated for each cell type per spot to label the spots. We first obtained the top marker genes for each cell type by comparing the transcriptome versus the rest of the cell types included in our reference using the default Seurat pipeline. Cell type AUC scores were calculated using AUCell (v1.18.1), which computes the area under the curve for a given gene set (a unitless measure) per spot. This score, also called signature, reflects the activity of a gene set. For each cell type, we ranked the spots based on the AUC score and the estimated percentage value. Following these criteria, each spot was labeled as golden, high, low, or rest for the specific cell type.

- First, we defined the average percentage of each cell type based on the deconvolution analysis: for example, 32.2% for MSC and 16.0% for EC.
- Spots among the top 32.2% for MSC (16.0% for EC) in both AUC and deconvolution rankings were labeled as Golden.
- Spots among the top 64.4% for MSC (32.0% for EC) in both rankings, which are not golden, were labeled High.
- Spots with an AUC = 0, independently of the deconvolution percentage, were labeled as rest.
- The remaining spots were labeled as Low.
  MSC and EC golden spots were then used for deconvolution and AUC analysis of the specific subtypes using the single‐cell data generated in the study.

### 4.17 Hierarchical Clustering

The estimated cell‐type percentage values derived from the deconvolution were further used for hierarchical clustering with ISCHIA. Samples were merged, and the optimal number of clusters was 7. The cell‐type prevalence within each cluster was then calculated. The prevalence of each cluster in each sample was determined.

### 4.18 Cell Type Correlation

To determine whether two cell types co‐localize within the same spots, we conducted a correlation analysis of their AUC scores across the spots of interest (“golden” spots for the cell type of study). A Spearman correlation test was performed to assess the significance of this correlation. Additionally, to evaluate whether the correlation between the two cell types changes with distance from the spots of interest, we calculated the average AUC score in the six nearest spots surrounding each spot of interest. The AUC score of the target cell type within its respective “golden” spot was then correlated with the average AUC score of the other cell types in the surrounding spots. A Spearman correlation test was again used to assess the significance of this correlation.

### 4.19 Statistical Analyses

Statistical analysis was carried out in R (v4.1.3). Tests used to evaluate statistical significance are detailed in each method section. Multiple‐testing correction was applied as specified for each analysis.

## Author Contributions

I.A.C., I.C., and M.C. processed human BM samples. I.C. analyzed the scRNA‐seq data and performed the cell‐to‐cell interactome. A.R.L.‐P. analyzed the spatial transcriptomic data. J.R., I.S.G., M.M.B., and D.Q.A. provided the BM samples and biopsies. P.S.M.‐U., P.A.‐R., and A.V.‐Z. performed the scRNA‐seq experiments. D.A. and A.L. performed the fluorescence‐activated cell sorting. I.A.C., I.C., and M.C. interpreted the data and wrote the paper. S.S., P.R.‐C., P.S.M.‐U., and P.A.‐R. performed the spatial transcriptomic experiments. M.C. and L.C.‐D. performed the histologic analysis. D.G.‐C., with help from J.Y., L.S., R.L., and J.T., supervised the computational work. M.A.‐M. and E.M. contributed by providing the myelofibrosis samples. Á.L.‐J. provided elderly BM biopsies. I.A.C. and B.S. conceptualized the study. F.P., D.G.‐C., and I.A.C. conceived and directed the research project. All authors actively participated in the discussions underlying this manuscript. F.P., D.G.‐C., I.A.C., I.C., and M.C. discussed the results and wrote the final manuscript. All authors contributed to, read, and approved the final manuscript.

## Funding

Instituto de Salud Carlos III and co‐financed by ERDF A way of making Europe (PI20/01308, PI23/00516). CIBERONC (CB16/12/00489), RICORS TERAV and TERAV Plus (RD21/0017/0009) (RD24/0014/0010). Departamento de Industria Gobierno de Navarra (AGATA 0011‐1411‐2020‐000010/0011‐1411‐2020‐000011). Departamento de Salud Gobierno de Navarra. All of them to F.P. Spanish Government through project PID2019‐111192GA‐I00 (MICINN) to D.G.‐C. Marie Curie grant from the European Commission (H2020‐MSCA‐IF‐837491) to I.A.C. AECC Predoctoral Fellowship (PRDNA19006CENZ) to I.C. FPU Fellowship from Ministerio de Ciencia, Innovación y Universidades (FPU22/03283) to M.C.

## Conflicts of Interest

The authors declare no conflicts of interest.

## Supporting information


**Figure S1:** Isolation of human BM EC and MSC. (A) Representative image of processed human BM sample obtained from orthopedic hip replacement surgery. It contains a BM liquid fraction together with bone pieces. (B) Sorting gating strategy for isolation of human BM EC (TO‐PRO‐3^−^, CD16^−^, CD56^−^, CD45^−^, CD235^−^, Lin^−^, CD31^+^, CD9^+^) and MSC (TO‐PRO‐3^−^, CD16^−^, CD56^−^, CD45^−^, CD235^−^, CD31^−^, Lin^−^, CD271^+^).

**Figure S2:** scRNA‐seq analysis of young and aged BM endothelial and stromal cells. (A) Bar plots showing the number of cells per cluster and dataset. (B) UMAP representation of human BM microenvironment cells split into young and elderly datasets, colored by cluster. (C) Dot plot of canonical markers used to define EC, MSC, and hematopoietic populations. The dot size represents the percentage of cells within the cluster that express each gene, and the color indicates the average expression level (D, E) UMAP visualization of well‐known markers for EC (D) and MSC (E). (F, G) Bar plot depicting the total number of EC (F) and MSC (G) per age group (young and elderly).

**Figure S3:** Signatures and markers defining the vascular beds in the human BM endothelial compartment. (A) UMAP visualization of artery, sinusoid, capillary, and vein signature scores. (B) Violin plot displaying the expression of cell cycle‐related genes. (C) Violin plot showing the expression of *RAB13* ^+^‐arterial‐like EC in all EC. (D) Significant gene sets derived from the GO ORA conducted with the markers defining each vascular state.

**Figure S4:** Characterization of the aged endothelial compartment. (A) Stacked bar plots representing the proportion of cells per individual (left panel) and age group (right panel) in each cluster. (B) Relative differences in cell proportions for each vascular state, comparing young and aged EC. Red and blue represent clusters statistically significant (FDR < 0.05 and absolute log2 fold change > 1) in young and aged EC, respectively. Larger log2 fold changes indicate a higher proportion of cells. (C) Violin plot showing the expression of genes upregulated in the venous cluster. (D) Violin plot displaying the expression of *SELE* among the vascular states. (E) UMAP visualization of the distribution of RAB13^+^ cells in young (left) and aged (right) EC. (F) Violin plot displaying the percentage of RBP per EC cluster. (G) Split violin plots showing the percentage of RBP in EC clusters split by age group. (H) GSEA plot showing the enrichment of “Myc targets” and “Senescence” terms in RAB13^+^ EC. (I) Network of ZEB1 regulon enriched in RAB13^+^ EC. (right) EC.

**Figure S5:** Validation of age‐associated enrichment of RAB13^+^ EC and related pathways. (A) IF staining in the FFPE BM biopsies (Table S5) of the young sample 7 (upper panel) and the elderly sample 8 (lower panel). Scale bars: 50 μm. Left panel: CD31 (green), EMCN (red), and DAPI (blue). White arrows indicate EC (CD31^+^ EMCN^+^). Middle panel: RAB13 (green), EMCN (red), and DAPI (blue). White arrows indicate RAB13 EC (RAB13^+^ EMCN^+^). Right panel: Alpha Smooth Muscle Actin (aSMA) (green), VE‐Cadherin (VECAD) (red), and DAPI (blue). White arrows indicate EC (VCAD^+^), and yellow arrow indicates vascular smooth muscle cells (aSMA^+^). (B) IF staining in the FFPE BM biopsies (Table S5) of the young sample 11 (left) and the elderly sample 13 (right). Scale bars: 50 and 100μm. Upper panel: Ribosomal marker pS6 (green), CD31 (red), and DAPI (blue). Green and red arrows indicate colocalization of pS6 EC (pS6^+^ CD31^+^). Lower panel: Ribosomal marker pS6 (green), RAB13 (red), and DAPI (blue). Green and red arrows indicate colocalization of pS6 EC‐RAB13^+^ (pS6^+^ RAB13^+^). (C) IF staining in the FFPE BM biopsies (Table S5) of the young sample 11 (left) and elderly sample 13 (right). Scale bars: 50 and 100μm. Upper panel: TF ZEB1 (green), CD31 (red), and DAPI (blue). Green and red arrows indicate colocalization of ZEB1 EC (ZEB1^+^ CD31^+^). Middle panel: TF ZEB1 (green), RAB13 (red), and DAPI (blue). Green and red arrows indicate colocalization of ZEB1 EC‐RAB13^+^ (ZEB1^+^ RAB13^+^). Lower panel: TF ZEB1 (green), aSMA (red), and DAPI (blue). Green and red arrows indicate colocalization of ZEB1 arterial EC (ZEB1^+^ aSMA^+^).

**Figure S6:** Transcriptional remodeling of sinusoids in the aged BM EC. (A) Summary heatmap of the number and effect size of all age‐DEGs (FDR < 0.05; abs(log2FC) > 0.5) identified within each vascular state. Color represents the *z*‐score of the log2FC. (B) Volcano plot of the DEGs between young and aged sinusoids. The y‐axis represents the −log10 (*p*‐value), and the x‐axis represents the log2FC. The color of the dot denotes the age group for which DEGs were detected, with gray dots representing non‐significant genes. (C) Violin plots showing the expression of aging‐related prothrombotic and matrix‐associated genes upregulated in aged sinusoids. (D) Cnetplot showing the links between genes and biological processes upregulated in aged sinusoids. Node size reflects the number of significantly enriched genes in the node and colors the log_2_ Fold change expression of each gene. (E) GSEA plot of “Oxidative phosphorylation” and “Adipogenesis” terms significantly enriched in aged sinusoids. (F) Cnetplot showing the relationship among individual GO terms and genes downregulated in aged sinusoids. Node size indicates the number of significantly enriched genes in the node and colors the log_2_ Fold change expression of each gene.

**Figure S7:** Further transcriptional profiling of THY1^+^ Fibro‐MSC. (A) Violin plots showing the marker expression of THY1^+^ Fibro‐MSC cluster. (B) Left: UMAP visualization of the Bandyopadhyay et al. (2024) dataset. Colors denote the identified stromal subtypes. Right: UMAP projection showing the distribution of stromal cell type annotations from our study in the Bandyopadhyay et al. (2024) dataset. (C) UMAP visualization of APOD and GSN genes defining APOD^+^GSN^high^ MSC described in Bandyopadhyay et al. (2024) dataset. (D) UMAP plot illustrating the distribution of stromal cell subtypes, including adipo‐lineage clusters. (E) UMAP visualization of the expression of adipogenic‐related genes upregulated in adipo‐lineage cells. (F) Stacked bar plots representing the proportion of cells per individual in each stromal subpopulation. (G) Split violin plots showing the expression of aging‐related regulators upregulated in the THY1^+^ Fibro‐MSC cluster split by age. (H) Violin plot showing the expression of collagen‐associated genes related to TGF‐β signaling pathway upregulated in the THY1^+^ Fibro‐MSC cluster. (I) Aged‐split violin plots showing the expression of *IGFBP6* and *IGFBP7* in MSC clusters. (J) GSEA plot of “Epithelial‐Mesenchymal (Epi‐Mes) Transition” term significantly enriched in THY1^+^ Fibro‐MSC cluster.

**Figure S8:** Validation of age‐associated enrichment of THY1^+^ Fibro‐MSC and related pathways. (A) IF staining in the FFPE BM biopsies (Table S5) of the young sample 11 (left) and the elderly sample 14 (right). Scale bars: 50 μm. Fibronectin (green), CD271 (red), and DAPI (blue). Green and red arrows indicate colocalization of fibronectin MSC (fibronectin^+^ CD271^+^). (B) IF staining in the FFPE BM biopsies (Table S5) of the young sample 11 (left) and the elderly sample 11 (right). Scale bars: 50 μm. Type I collagen (green), CD271 (red), and DAPI (blue). Green and red arrows indicate colocalization of type I collagen MSC (type I collagen^+^ CD271^+^). (C) IF staining in the FFPE BM biopsies (Table S5) of the young sample 10 (left) and the elderly samples 8 (right). Scale bars: 50 μm. Phosphorylated SMAD2 (pSMAD2) (green), CD271 (red), and DAPI (blue). Green and red arrows indicate colocalization of pSMAD2 MSC (pSMAD2^+^ CD271^+^). (D) Quantification of THY1^+^ cells (CD90^+^) (red), MSC (PRRX1^+^) (green), and THY1^+^ MSC (coexpression CD90^+^ and PRRX1^+^) in young, elderly, and myelofibrosis (MF) samples. Bars represent the mean ± SEM. ns: not significant. (E) Quantification of fibrotic tissue area in young, elderly, and MF samples. The fibrotic tissue area was measured in Masson's trichrome‐stained images by two approximations. The percent of fibrotic tissue area/whole tissue area and the circularity of the cells. Bars represent the mean ± SEM. ns: not significant. (F) Left panel: IF staining of THY1^+^ Fibro stromal cells (CD90^+^) (red), (PRRX1^+^) (green), and nucleus (DAPI) (blue) in FFPE biopsy samples from MF patients. Scale bars: 100 μm. Right panel: Masson‐Trichrome staining for fibrotic tissue of FFPE biopsies from the same tissue area. White arrows indicate THY1^+^ Fibro MSC (PRRX1^+^ CD90^+^).

**Figure S9:** Age‐dependent transcriptional changes in MSC. (A) UMAP visualization of MSC in young (top) and aged (bottom). (B) Split violin plots showing the expression of down‐regulated genes in aged MSC split by age group. (C, D) Bar charts of enriched GO terms from ORA (*p* < 0.05) comparing DEGs within young and aged MSC. The horizontal axis represents the −log10 of *p*‐values. (C) represents downregulated terms and (D) upregulated terms in aged MSC. (E) Split violin plots showing the expression of oxidative metabolism‐related genes upregulated in aged MSC. (F) GSEA plot of “Oxidative phosphorylation” and “Adipogenesis” terms significantly enriched in aged MSC. (G) Split violin plots showing the downregulation of *ADAMTS9* and *ADAMTSL3* in aged MSC.

**Figure S10:** Additional information about the remodeling of the BM interactome during aging. (A, B) Chord diagrams displaying unique interactions specific to young (A) and aged (B) BM microenvironment cells. (C) Chord diagrams showing interactions involving *TGFB1* signaling in the aged BM. Colors and widths represent the signal senders and the strength of interactions, respectively. (D) Chord diagrams showing interactions through *MIF*, *CD74*, and *CD99* ligands in the aged BM. Colors and widths represent the signal senders and the strength of interactions, respectively.

**Figure S11:** Additional information about spatial transcriptomics analysis of the human BM. (A) Pie charts illustrating the proportion of each cell type contributing to the transcriptomic signature of each spot in young (left) and elderly (right) BM samples from deconvolution analysis. (B) Pie charts illustrate the proportion of each cell type that contributes significantly to the transcriptomic signature of each spot in young (left) and elderly (right) BM samples. (C) Cell type proportions per spot derived from deconvolution analysis using the Bandyopadhyay et al. (2024) dataset as a reference. (D) Spatial distribution pattern of the signature score for EC (top panels) and MSC (bottom panels) in young (left side) and elderly (right side) BM samples. (E) Correlation between cell‐type proportions obtained through deconvolution (percentage) and the signature scores of EC (top) and MSC (bottom) in young (left side) and elderly (right side) BM samples. (F) Spatial distribution of “golden”, “high”, “low”, and “rest” spots based on the top‐ranking overlap between deconvolution and spot signature analyses (detailed in the methods section) in young (left side) and elderly (right side) BM samples using EC (top panels) and MSC (bottom panels) as examples. (G) H&E staining of young (upper panel) and elderly (bottom panel) BM samples.

**Figure S12:** Additional information about the spatial expression patterns of aging‐related gene expression changes in EC. (A) Spatial expression patterns of the transcription factor *YBX1* in young (left side) and elderly (right side) BM samples. (B) Violin plots illustrating the spatial expression levels of CLU and CD74 genes in young (red) and aged (blue) EC. (C) Box plots showing the scores for oxidative metabolism genes set in young (red) and aged (blue) EC. (D) Violin plots illustrating the spatial expression levels of VIM and GSN genes in young (red) and aged (blue) EC (E) Violin plots showing *BMPR2* spatial gene expression levels in young (red) and aged (blue) EC. (F) Spatial expression patterns of the *JAG1‐NOTCH3* L‐R pair in EC golden spots of young (left panels) and elderly (right panels) BM samples. (G) Plots showing the correlation between EC signature and DC signature of EC‐labeled spots in young (left‐red) and elderly (right‐blue) samples. (H). Violin plots of *FN1* spatial expression levels in young (red) and aged (blue) EC.

**Figure S13:** Donor‐balanced bootstrap analysis of EC subtypes showing one of the iterations as an example. (A) Number of EC sampled during balanced downsampling (*n* = 50 cells per donor) grouped per donor (left) and age group (right). (B) Number of RAB13^+^ EC per individual after downsampling. (C) UMAP embedding of downsampled EC colored by donor identity. (D). UMAP representation of downsampled EC split into young and aged group, colored by subtype. (E) Stacked bar plots representing the proportion of each EC subtype per individual (left panel) and age group (right panel). (F) Relative differences in cell proportions for each vascular state, comparing young and aged EC. Red and blue represent clusters statistically significant (FDR < 0.05 and absolute log2 fold change > 1) in young and aged EC, respectively. (G,H) Stacked bar plots representing the proportion of cells in each EC subtype per individual (G) and age group (H). (I) UMAP plot illustrating the recomputed dimensional reduction and clustering of downsampled EC colored by subtype (left) and cluster (right). (J) Heatmap showing Jaccard similarity index between the recomputed clusters (X axis) and EC subtypes (Y axis). (K) Clustering robustness metrics across iterations, including Jaccard similarity (left) and Adjusted Rand Index (ARI) (K), demonstrating stability of the RAB13^+^ EC cluster.

**Figure S14:** Donor‐balanced bootstrap analysis of MSC subtypes showing one of the iterations as an example. (A) Number of MSC sampled during balanced downsampling (*n* = 50 cells per donor) grouped per donor (left) and age group (right). (B) Number of THY1^+^ Fibro‐MSC per individual after downsampling. (C) UMAP plot illustrating the distribution downsampled MSC colored by individual. (D) UMAP representation of downsampled MSC split into young and aged group, colored by subtype. (E) Stacked bar plots representing the proportion of each MSC subtype per individual (left panel) and age group (right panel). (F) Relative differences in cell proportions for each MSC population, comparing young and aged MSC. Red and blue represent clusters statistically significant (FDR < 0.05 and absolute log2 fold change > 1) in young and aged MSC, respectively. (G, H) Stacked bar plots representing the proportion of cells in each MSC subtype per individual (G) and age group (H). (I) UMAP plot illustrating the recomputed dimensional reduction and clustering of downsampled MSC colored by subtype (left) and cluster (right). (J) Heatmap showing Jaccard similarity index between the recomputed clusters (X axis) and MSC subtypes (Y axis). (K) Clustering robustness metrics across iterations, including Jaccard similarity (left) and Adjusted Rand Index (ARI) (K), demonstrating stability of the THY1^+^ fibro‐MSC cluster.

**Figure S15:** Concordance between donor‐level and single‐cell differential expression analysis. (A) Correlation of log2 fold‐change estimates for common RAB13^+^ EC marker genes between single‐cell and pseudobulk analyses. (B) Correlation of log2 fold‐change estimates for common THY1^+^ fibro‐MSC marker genes between single‐cell and pseudobulk analyses. (C) Correlation of log2 fold‐change estimates for common age‐associated DEGs between single‐cell and pseudobulk analyses (Spearman *ρ* = 0.89). (D) Correlation of log2 fold‐change estimates for common age‐associated DEGs between single‐cell and pseudobulk analyses (Spearman *ρ* = 0.89).

**Figure S16:** Sensitivity analysis of endothelial cell populations after exclusion of dominant donor Elderly 4. (A) UMAP projection of EC after exclusion of donor Elderly 4, colored by donor identity (left) and endothelial subtypes (right). (B) Differential abundance analysis showing fold changes in endothelial subtypes between aged and young donors following donor exclusion, with FDR‐adjusted *p*‐values indicated. (C) Stacked bar plots representing the proportion of each EC subtype per age group. (D) Stacked bar plots representing the proportion of cells in each EC subtype per individual (left) and age group (right). (E) Recomputed UMAP embeddings after donor exclusion colored by donor identity (left), endothelial subtype (middle), and cluster assignment (right). (F) Heatmap of cluster correspondence between the endothelial subtypes from the original dataset and the re‐assigned clusters. (G) Venn diagram showing overlap of RAB13^+^ EC marker genes identified in the original dataset versus after exclusion of Elderly 4.

**FIGURE S17:** Sensitivity analysis of endothelial cell populations after exclusion of dominant donor Elderly 3. (A) UMAP projection of MSC after exclusion of donor Elderly 3, colored by donor identity (left) and mesenchymal subtypes (right). (B) Differential abundance analysis showing fold changes in mesenchymal subtypes between aged and young donors following donor exclusion, with FDR‐adjusted *p*‐values indicated. (C) Stacked bar plots representing the proportion of each mesenchymal subtype per age group. (D) Stacked bar plots representing the proportion of cells in each mesenchymal subtype per individual (left) and age group (right). (E) Recomputed UMAP embeddings after donor exclusion colored by donor identity (left), mesenchymal subtype (middle), and cluster assignment (right). (F) Heatmap of cluster correspondence between the mesenchymal subtypes from the original dataset and the re‐assigned clusters. (G) Venn diagram showing overlap of THY1^+^ fibro‐MSC marker genes identified in the original dataset versus after exclusion of Elderly 3.

**Table S1:** Summary of human BM samples from young and aged donors used for scRNA‐seq analysis.

**Table S2:** Cluster‐specific marker genes. Annotated BM cell populations by canonical markers.

**Table S3:** Established EC markers (arteries, sinusoids, capillaries, and venous).

**Table S4:** RAB13^+^‐EC‐associated markers and enriched GO biological processes.

**Table S5:** Summary of human BM biopsies (young, aged, and myelofibrosis) used for imaging analyses.

**Table S6:** Aging‐associated cluster markers (arteries, sinusoids, capillaries, venous, and RAB13).

**Table S7:** DEGs in sinusoid EC cluster during aging.

**Table S8:** Established stromal cell markers (MSC, pericytes, and OLN‐primed cells).

**Table S9:** THY1^+^ Fibro‐MSC‐associated markers and enriched GO) biological processes.

**Table S10:** DEGs between young and aged MSC cells. DEGs between young and aged pericytes.

**Table S11:** GO enrichment analysis of up‐ and downregulated pathways in aged MSC.

**Table S12:** Significant ligand–receptor interactions in young and aged BM. Specific interactions between aged RAB13^+^‐EC and THY1^+^ MSC.

**Table S13:** Generalized linear mixed‐effects modeling of cell subpopulation proportions as a function of chronological age in EC and MSC.

## Data Availability

The scRNA‐seq data generated in this study has been deposited at GSE305558. Spatial raw and processed data from the elderly sample has been deposited at GSE269875. scRNA‐seq data used for spatial deconvolution was obtained from GSE253355.
