# Supplementary figures and images for: Single‐Cell Profiling Reveals RAB13 + Endothelial Cells and Profibrotic Mesenchymal Cells in Aged Human Bone Marrow
Source: Aging Cell. 2026 Apr 9;25(4):e70475. doi: 10.1111/acel.70475 (PMC13063395; doi:10.1111/acel.70475)

**A**

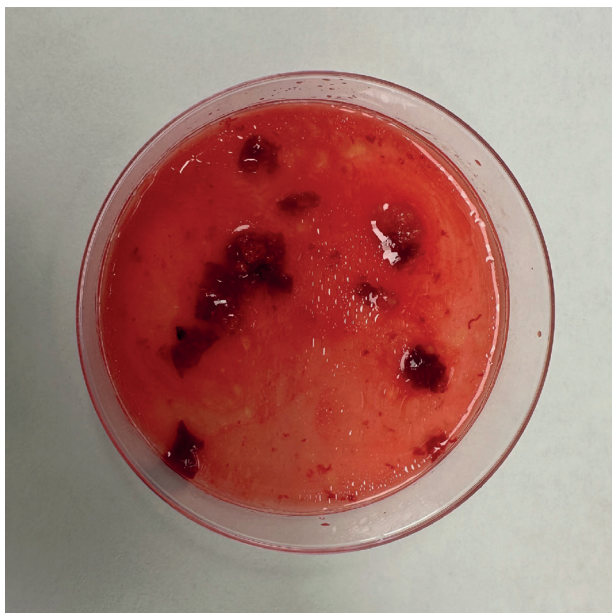

**B**

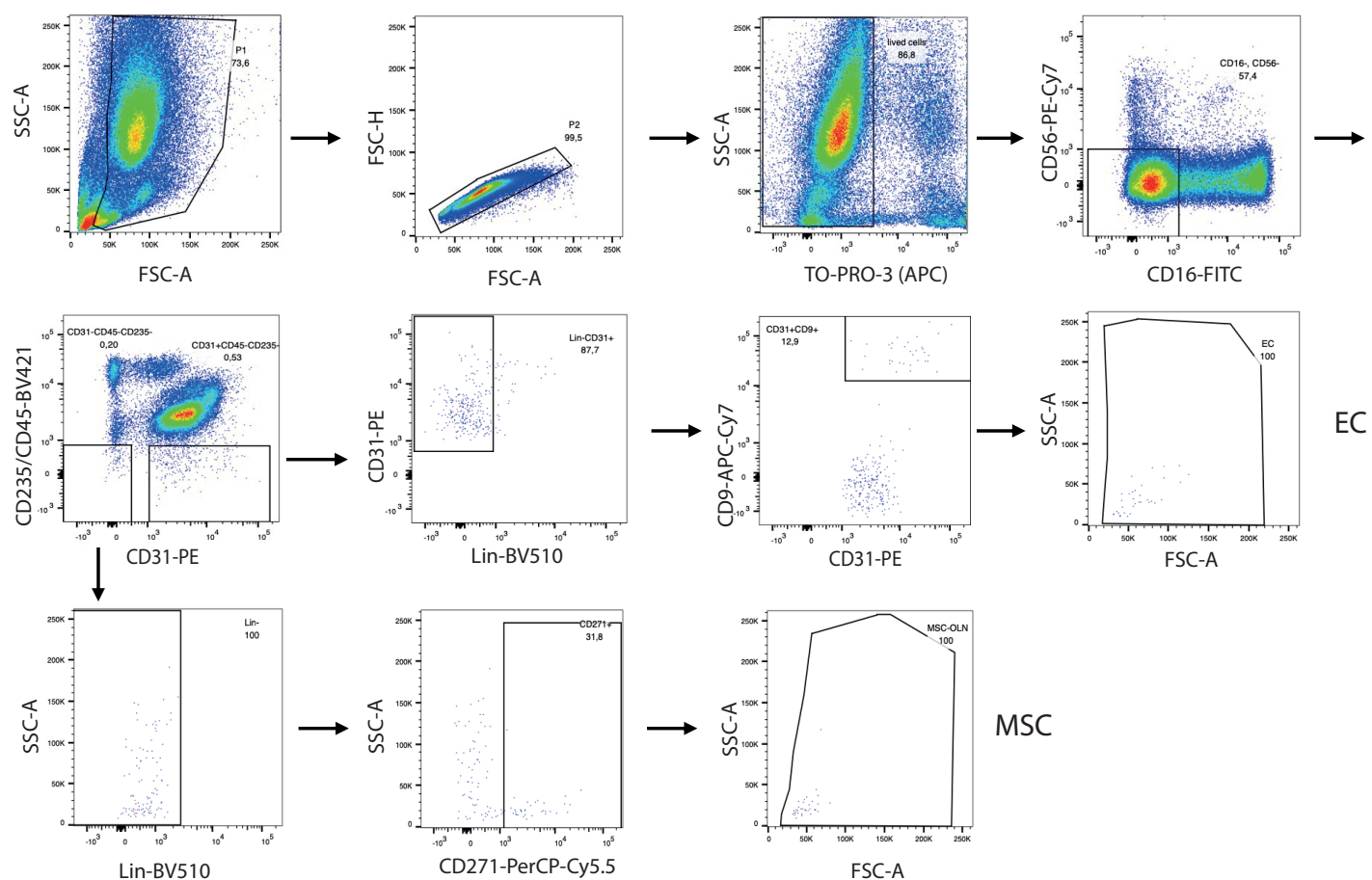

Supplement: Supplementary file 1 — Figure S1: Isolation of human BM EC and MSC. (A) Representative image of processed human BM sample obtained from orthopedic hip replacement surgery. It contains a BM liquid fraction together with bone pieces. (B) Sorting gating strategy for isolation of human BM EC (TO‐PRO‐3−, CD16−, CD56−, CD45−, CD235−, Lin−, CD31+, CD9+) and MSC (TO‐PRO‐3−, CD16−, CD56−, CD45−, CD235−, CD31−, Lin−, CD271+). [file ACEL-25-e70475-s004.pdf]

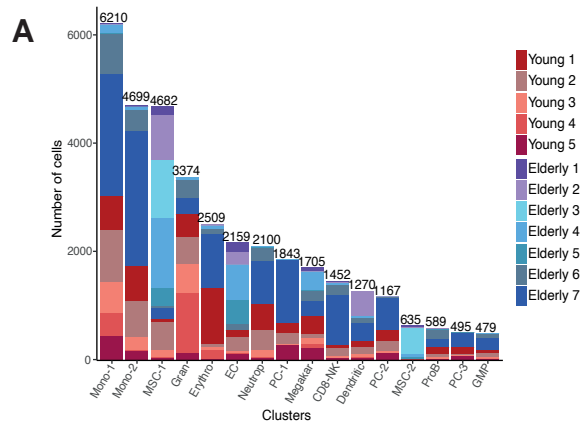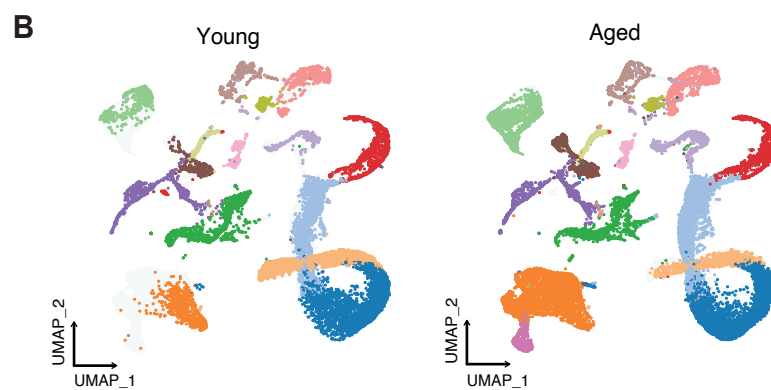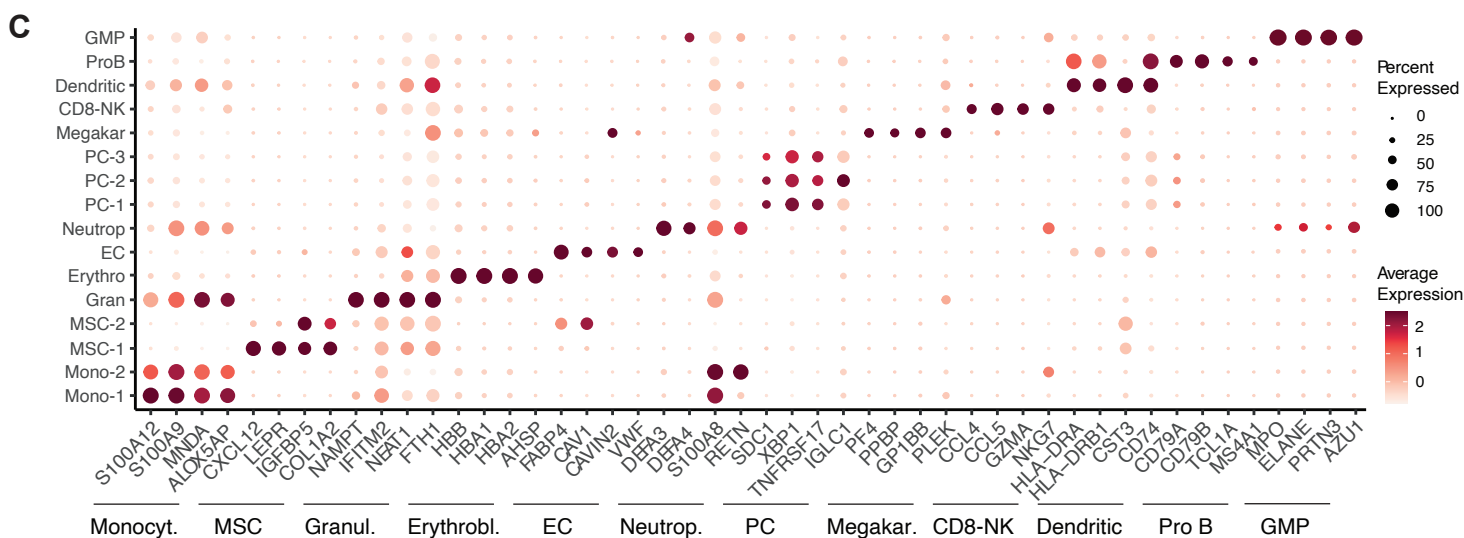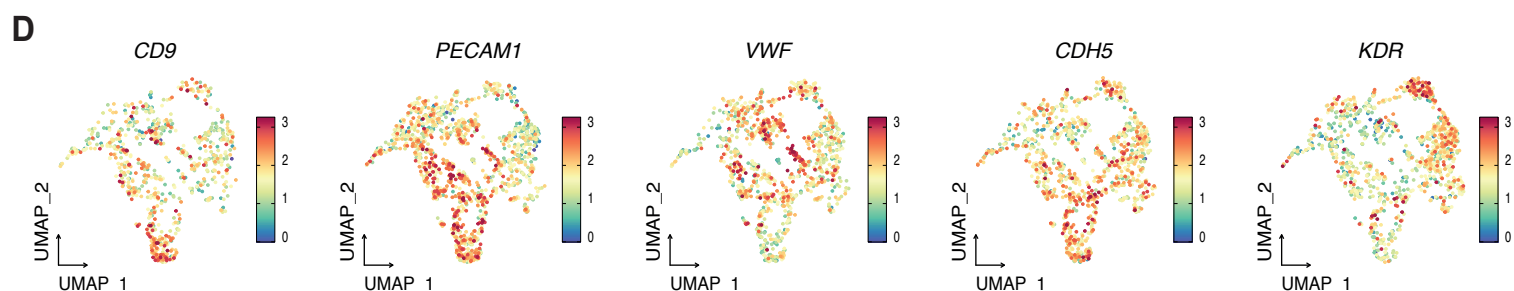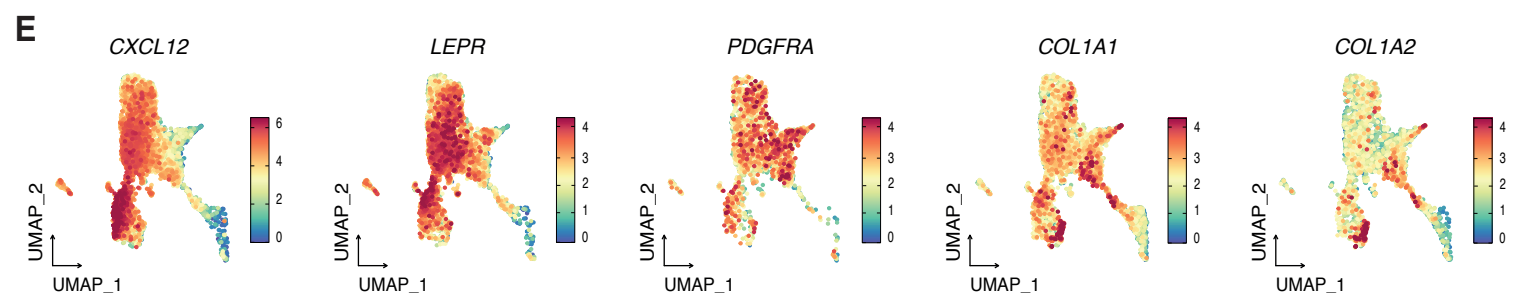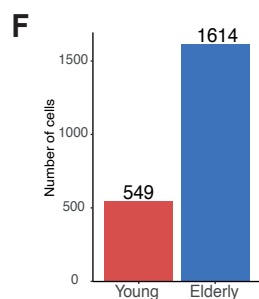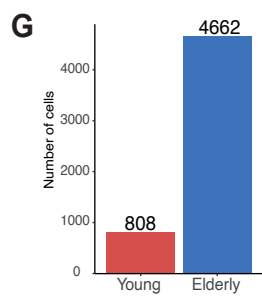

Supplement: Supplementary file 2 — Figure S2: scRNA‐seq analysis of young and aged BM endothelial and stromal cells. (A) Bar plots showing the number of cells per cluster and dataset. (B) UMAP representation of human BM microenvironment cells split into young and elderly datasets, colored by cluster. (C) Dot plot of canonical markers used to define EC, MSC, and hematopoietic populations. The dot size represents the percentage of cells within the cluster that express each gene, and the color indicates the average expression level (D, E) UMAP visualization of well‐known markers for EC (D) and MSC (E). (F, G) Bar plot depicting the total number of EC (F) and MSC (G) per age group (young and elderly). [file ACEL-25-e70475-s030.pdf]

A

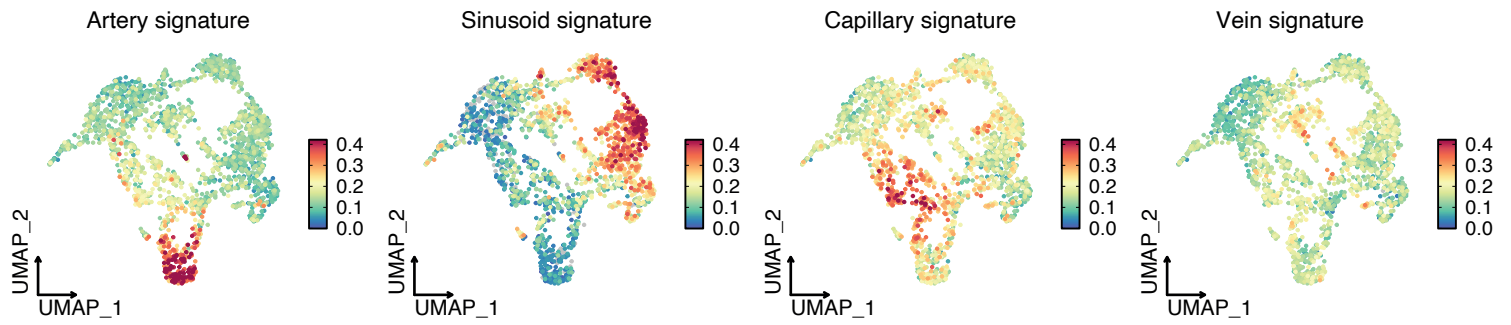

B

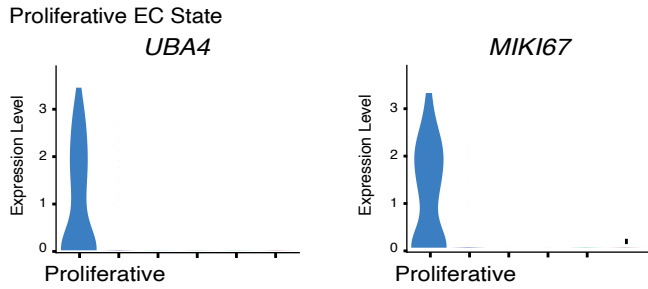

C

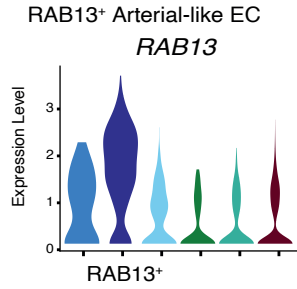

D

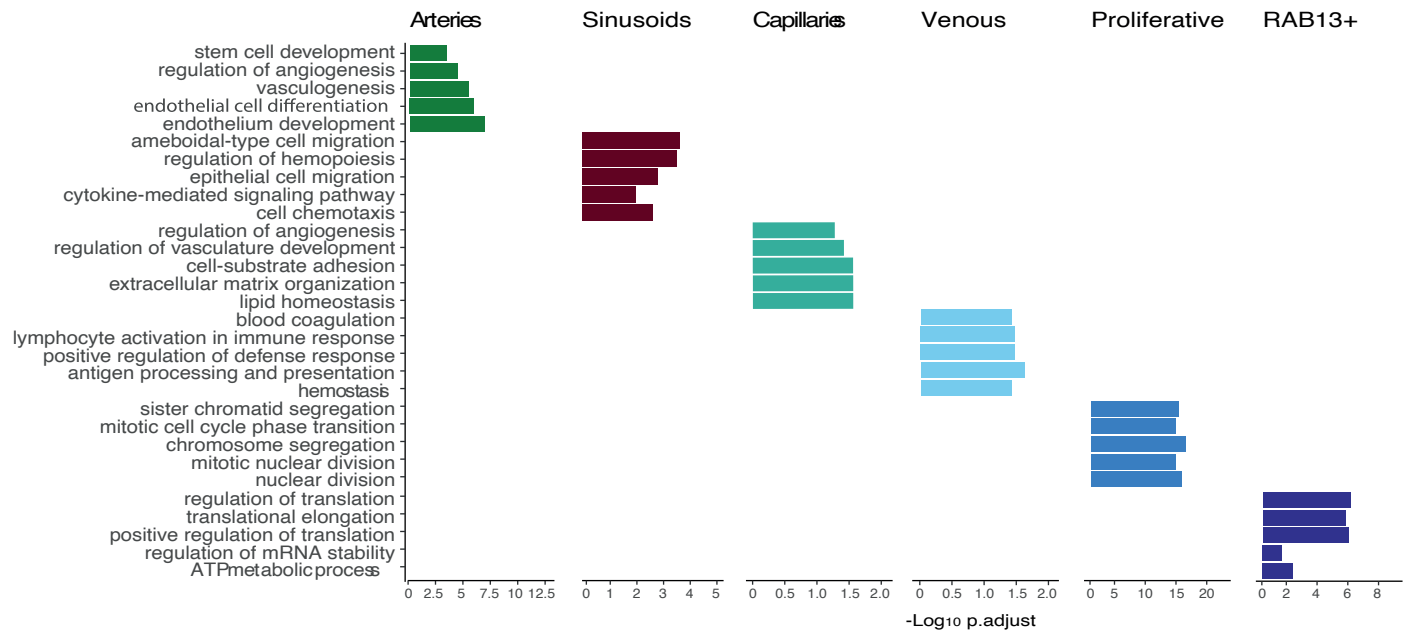

Supplement: Supplementary file 3 — Figure S3: Signatures and markers defining the vascular beds in the human BM endothelial compartment. (A) UMAP visualization of artery, sinusoid, capillary, and vein signature scores. (B) Violin plot displaying the expression of cell cycle‐related genes. (C) Violin plot showing the expression of RAB13 +‐arterial‐like EC in all EC. (D) Significant gene sets derived from the GO ORA conducted with the markers defining each vascular state. [file ACEL-25-e70475-s011.pdf]

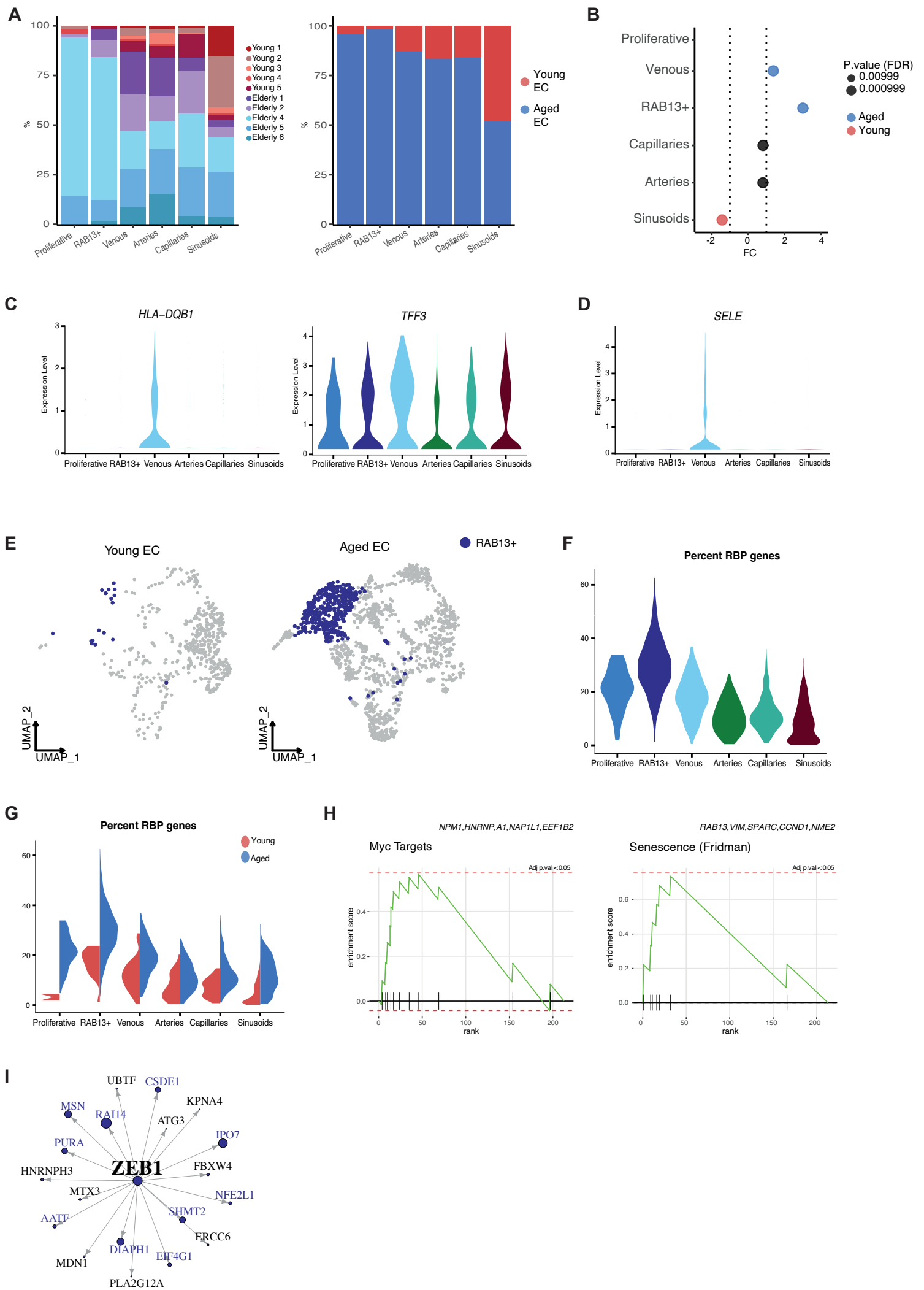

Supplement: Supplementary file 4 — Figure S4: Characterization of the aged endothelial compartment. (A) Stacked bar plots representing the proportion of cells per individual (left panel) and age group (right panel) in each cluster. (B) Relative differences in cell proportions for each vascular state, comparing young and aged EC. Red and blue represent clusters statistically significant (FDR < 0.05 and absolute log2 fold change > 1) in young and aged EC, respectively. Larger log2 fold changes indicate a higher proportion of cells. (C) Violin plot showing the expression of genes upregulated in the venous cluster. (D) Violin plot displaying the expression of SELE among the vascular states. (E) UMAP visualization of the distribution of RAB13+ cells in young (left) and aged (right) EC. (F) Violin plot displaying the percentage of RBP per EC cluster. (G) Split violin plots showing the percentage of RBP in EC clusters split by age group. (H) GSEA plot showing the enrichment of “Myc targets” and “Senescence” terms in RAB13+ EC. (I) Network of ZEB1 regulon enriched in RAB13+ EC. (right) EC. [file ACEL-25-e70475-s022.pdf]

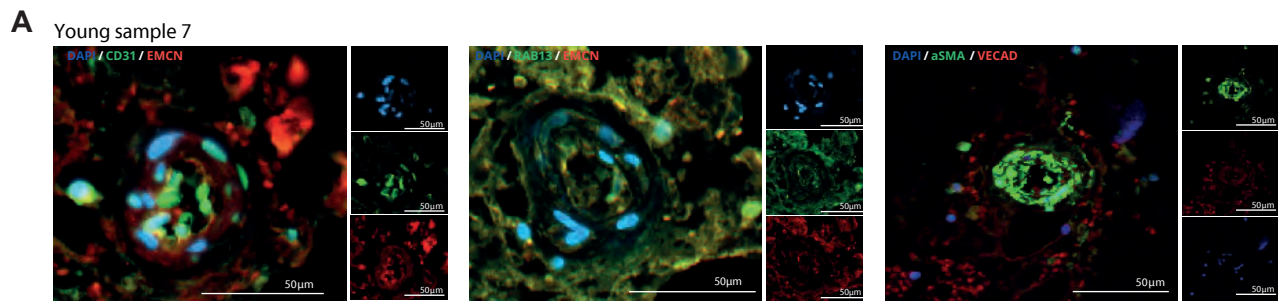

Elderly sample 8

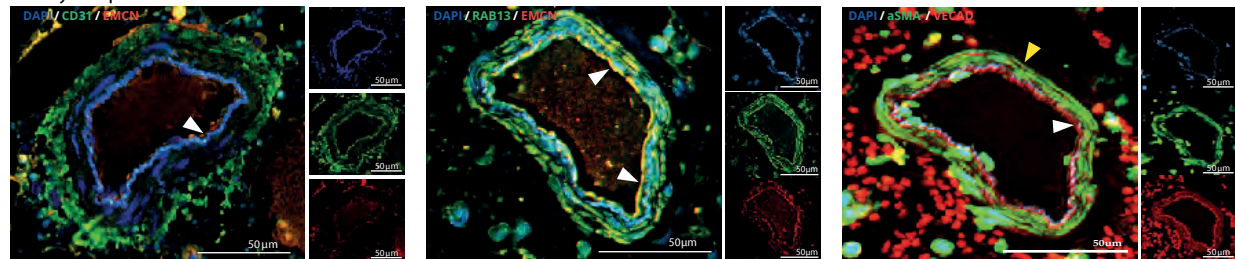

**B** Young sample 11

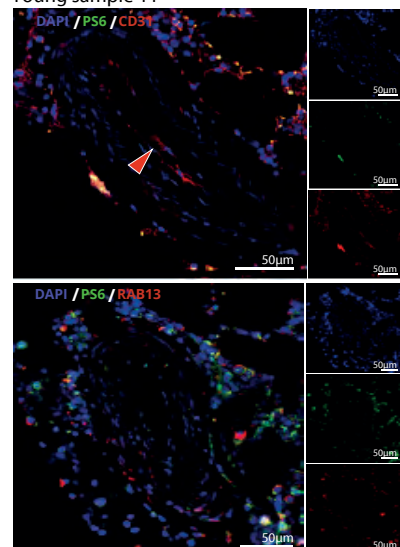

Elderly sample 13

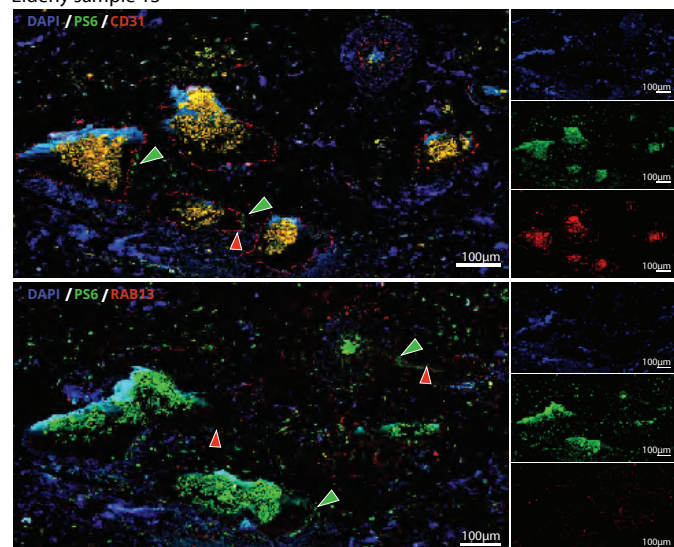

**C** Young sample 11

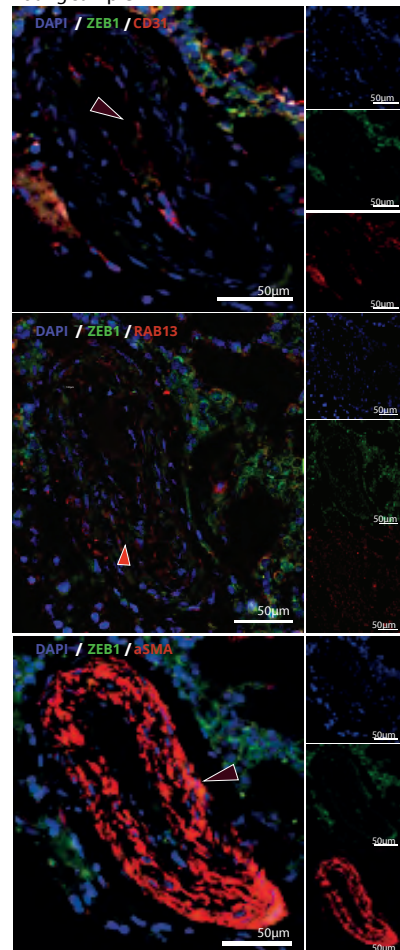

Elderly sample 13

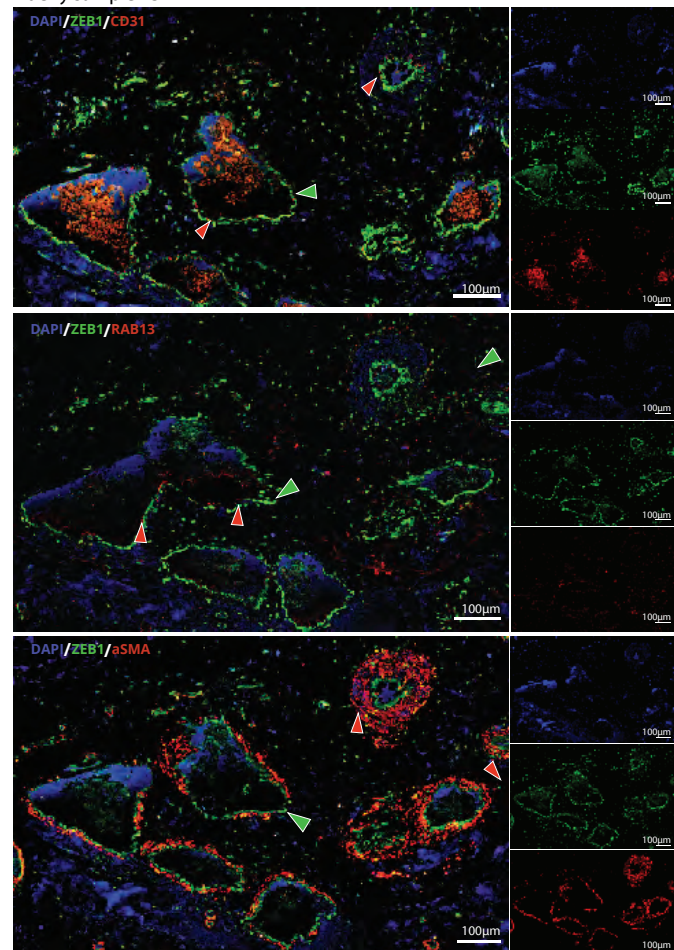

Supplement: Supplementary file 5 — Figure S5: Validation of age‐associated enrichment of RAB13+ EC and related pathways. (A) IF staining in the FFPE BM biopsies (Table S5) of the young sample 7 (upper panel) and the elderly sample 8 (lower panel). Scale bars: 50 μm. Left panel: CD31 (green), EMCN (red), and DAPI (blue). White arrows indicate EC (CD31+ EMCN+). Middle panel: RAB13 (green), EMCN (red), and DAPI (blue). White arrows indicate RAB13 EC (RAB13+ EMCN+). Right panel: Alpha Smooth Muscle Actin (aSMA) (green), VE‐Cadherin (VECAD) (red), and DAPI (blue). White arrows indicate EC (VCAD+), and yellow arrow indicates vascular smooth muscle cells (aSMA+). (B) IF staining in the FFPE BM biopsies (Table S5) of the young sample 11 (left) and the elderly sample 13 (right). Scale bars: 50 and 100μm. Upper panel: Ribosomal marker pS6 (green), CD31 (red), and DAPI (blue). Green and red arrows indicate colocalization of pS6 EC (pS6+ CD31+). Lower panel: Ribosomal marker pS6 (green), RAB13 (red), and DAPI (blue). Green and red arrows indicate colocalization of pS6 EC‐RAB13+ (pS6+ RAB13+). (C) IF staining in the FFPE BM biopsies (Table S5) of the young sample 11 (left) and elderly sample 13 (right). Scale bars: 50 and 100μm. Upper panel: TF ZEB1 (green), CD31 (red), and DAPI (blue). Green and red arrows indicate colocalization of ZEB1 EC (ZEB1+ CD31+). Middle panel: TF ZEB1 (green), RAB13 (red), and DAPI (blue). Green and red arrows indicate colocalization of ZEB1 EC‐RAB13+ (ZEB1+ RAB13+). Lower panel: TF ZEB1 (green), aSMA (red), and DAPI (blue). Green and red arrows indicate colocalization of ZEB1 arterial EC (ZEB1+ aSMA+). [file ACEL-25-e70475-s027.pdf]

**A**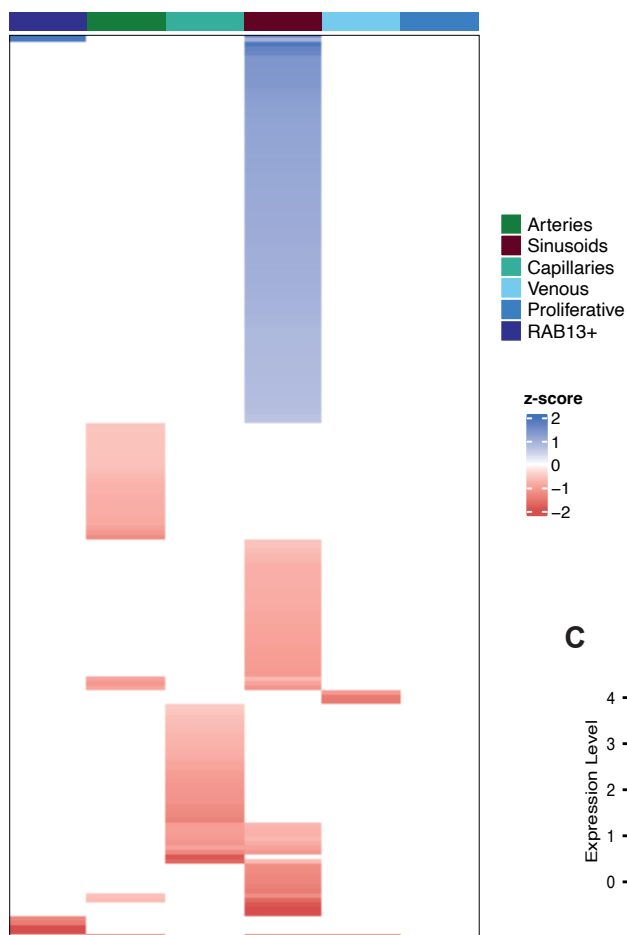**B**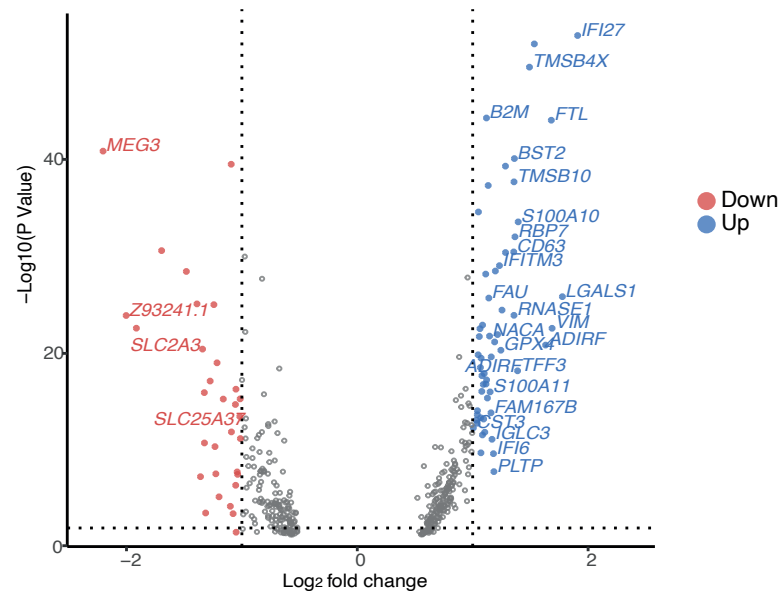**C**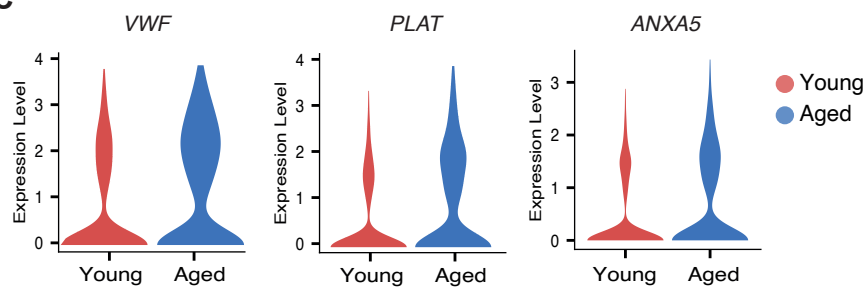**D**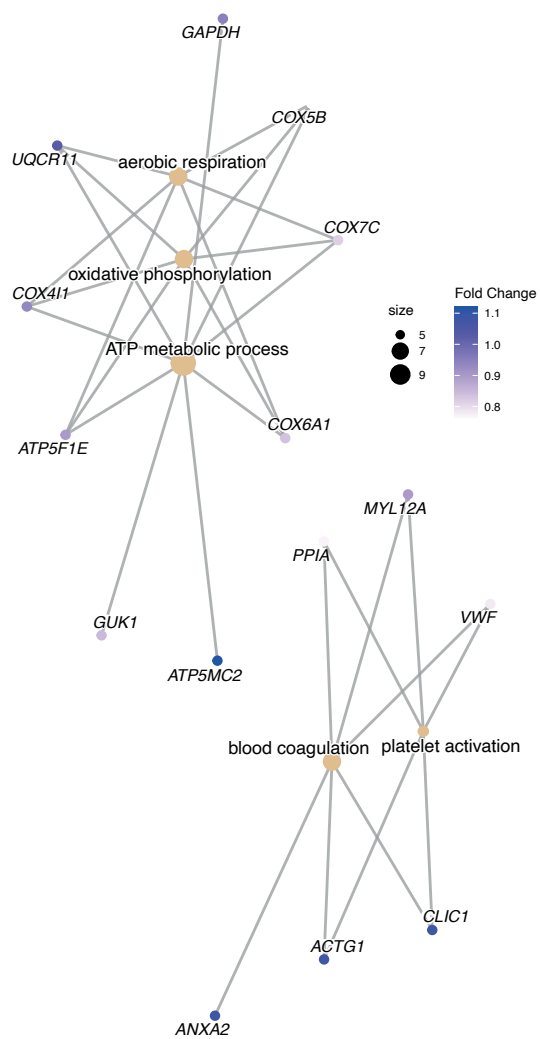**E**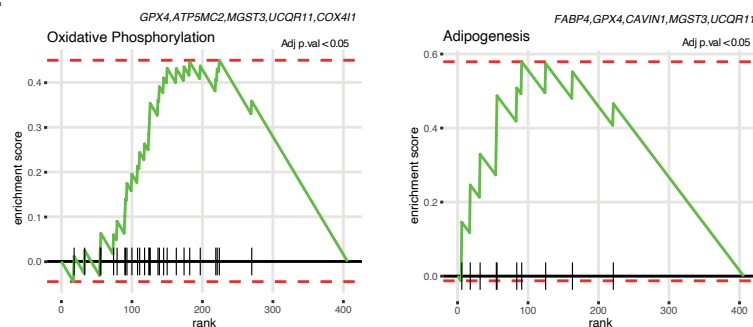**F**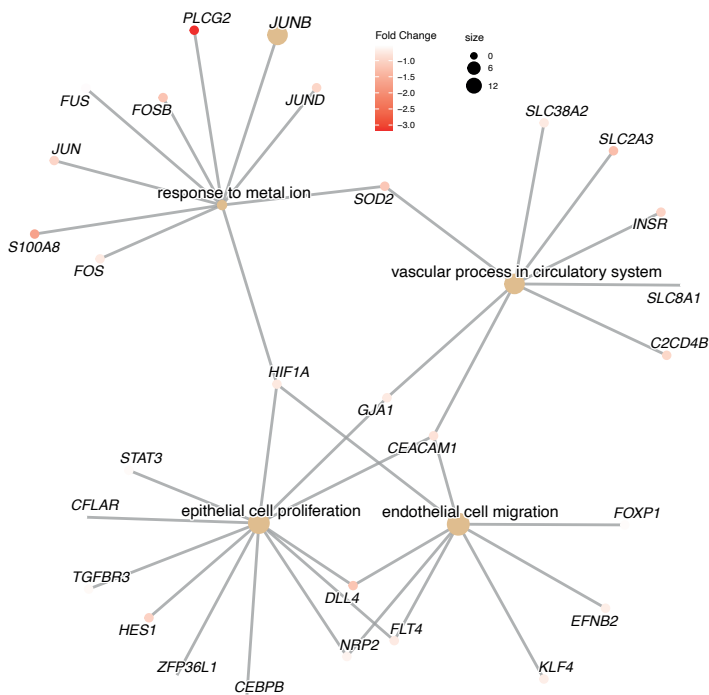

Supplement: Supplementary file 6 — Figure S6: Transcriptional remodeling of sinusoids in the aged BM EC. (A) Summary heatmap of the number and effect size of all age‐DEGs (FDR < 0.05; abs(log2FC) > 0.5) identified within each vascular state. Color represents the z‐score of the log2FC. (B) Volcano plot of the DEGs between young and aged sinusoids. The y‐axis represents the −log10 (p‐value), and the x‐axis represents the log2FC. The color of the dot denotes the age group for which DEGs were detected, with gray dots representing non‐significant genes. (C) Violin plots showing the expression of aging‐related prothrombotic and matrix‐associated genes upregulated in aged sinusoids. (D) Cnetplot showing the links between genes and biological processes upregulated in aged sinusoids. Node size reflects the number of significantly enriched genes in the node and colors the log2 Fold change expression of each gene. (E) GSEA plot of “Oxidative phosphorylation” and “Adipogenesis” terms significantly enriched in aged sinusoids. (F) Cnetplot showing the relationship among individual GO terms and genes downregulated in aged sinusoids. Node size indicates the number of significantly enriched genes in the node and colors the log2 Fold change expression of each gene. [file ACEL-25-e70475-s028.pdf]

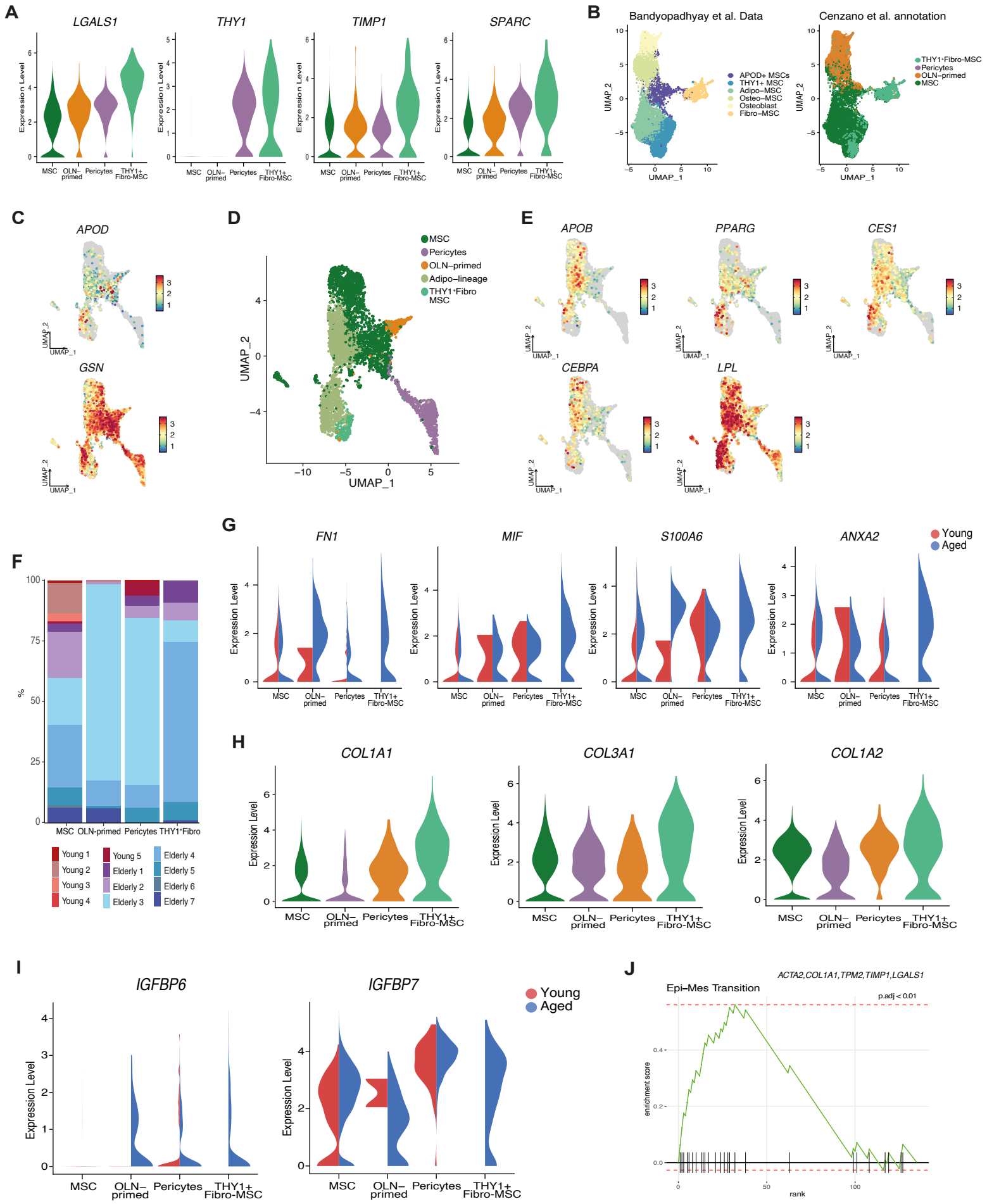

Supplement: Supplementary file 7 — Figure S7: Further transcriptional profiling of THY1+ Fibro‐MSC. (A) Violin plots showing the marker expression of THY1+ Fibro‐MSC cluster. (B) Left: UMAP visualization of the Bandyopadhyay et al. (2024) dataset. Colors denote the identified stromal subtypes. Right: UMAP projection showing the distribution of stromal cell type annotations from our study in the Bandyopadhyay et al. (2024) dataset. (C) UMAP visualization of APOD and GSN genes defining APOD+GSNhigh MSC described in Bandyopadhyay et al. (2024) dataset. (D) UMAP plot illustrating the distribution of stromal cell subtypes, including adipo‐lineage clusters. (E) UMAP visualization of the expression of adipogenic‐related genes upregulated in adipo‐lineage cells. (F) Stacked bar plots representing the proportion of cells per individual in each stromal subpopulation. (G) Split violin plots showing the expression of aging‐related regulators upregulated in the THY1+ Fibro‐MSC cluster split by age. (H) Violin plot showing the expression of collagen‐associated genes related to TGF‐β signaling pathway upregulated in the THY1+ Fibro‐MSC cluster. (I) Aged‐split violin plots showing the expression of IGFBP6 and IGFBP7 in MSC clusters. (J) GSEA plot of “Epithelial‐Mesenchymal (Epi‐Mes) Transition” term significantly enriched in THY1+ Fibro‐MSC cluster. [file ACEL-25-e70475-s014.pdf]

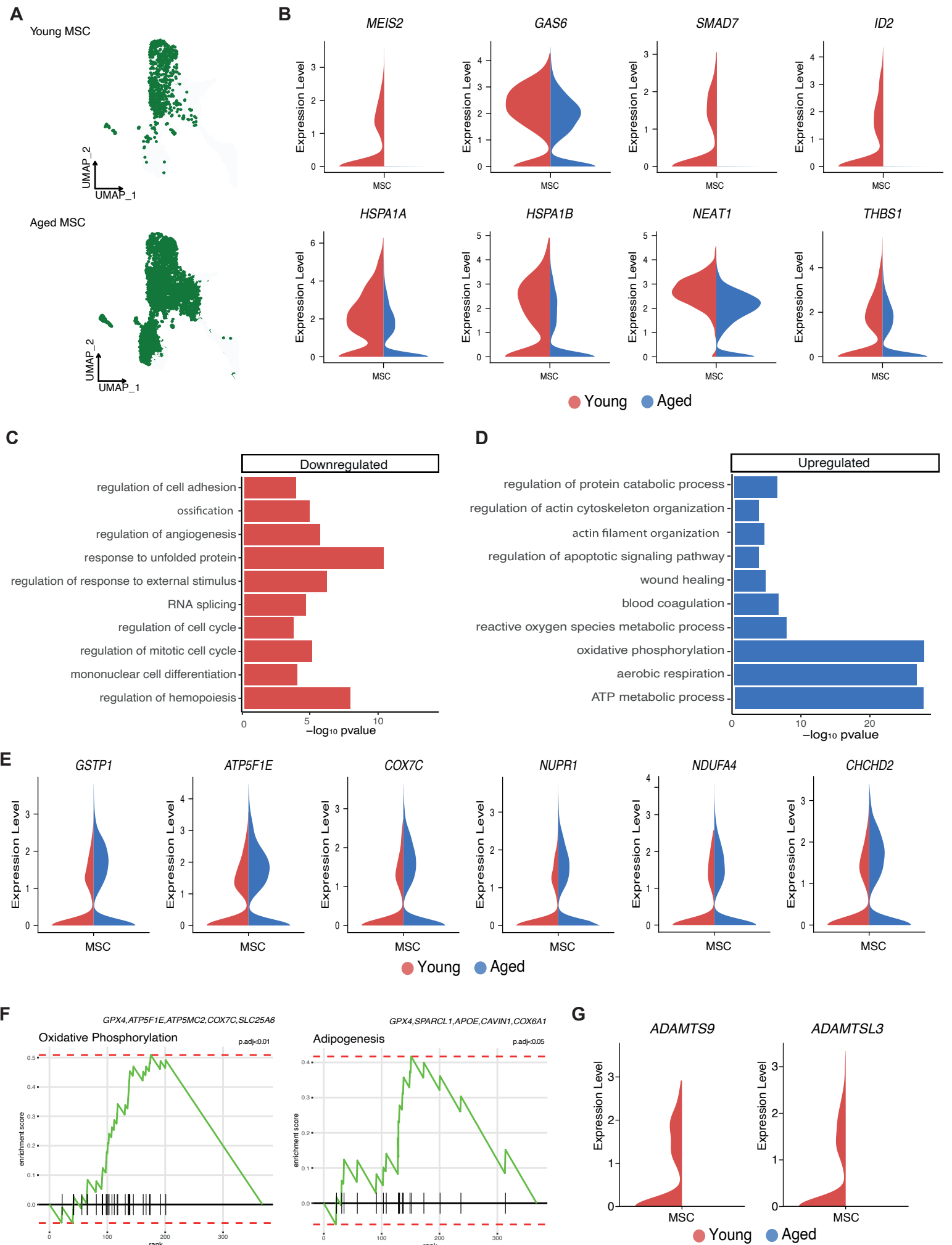

Supplement: Supplementary file 9 — Figure S9: Age‐dependent transcriptional changes in MSC. (A) UMAP visualization of MSC in young (top) and aged (bottom). (B) Split violin plots showing the expression of down‐regulated genes in aged MSC split by age group. (C, D) Bar charts of enriched GO terms from ORA (p < 0.05) comparing DEGs within young and aged MSC. The horizontal axis represents the −log10 of p‐values. (C) represents downregulated terms and (D) upregulated terms in aged MSC. (E) Split violin plots showing the expression of oxidative metabolism‐related genes upregulated in aged MSC. (F) GSEA plot of “Oxidative phosphorylation” and “Adipogenesis” terms significantly enriched in aged MSC. (G) Split violin plots showing the downregulation of ADAMTS9 and ADAMTSL3 in aged MSC. [file ACEL-25-e70475-s003.pdf]

**A**

## Young unique interactions

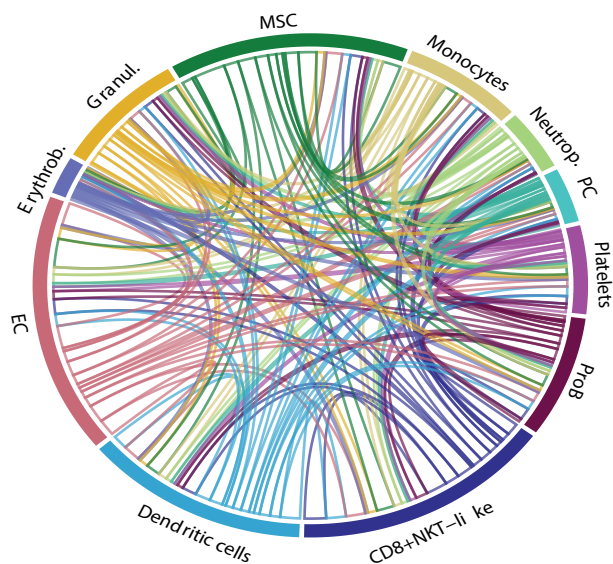**B**

### Aged unique interactions

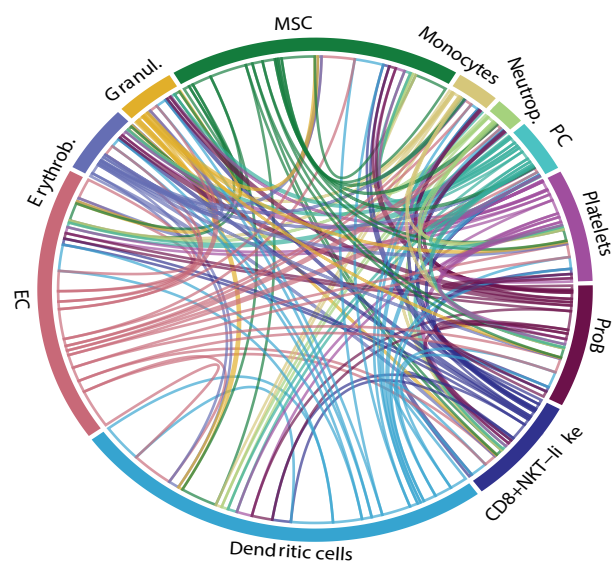

C

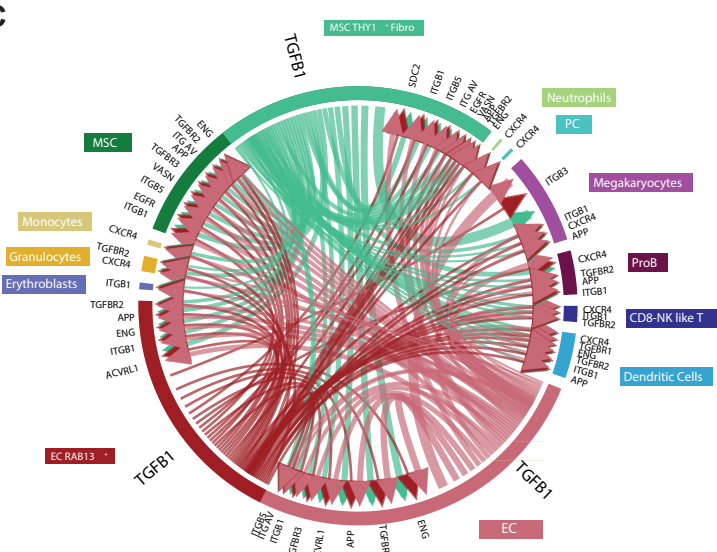

D

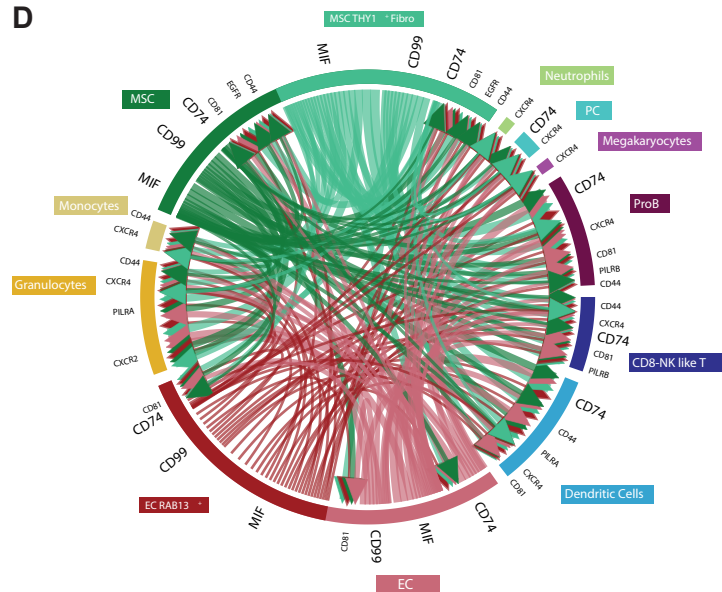

Supplement: Supplementary file 10 — Figure S10: Additional information about the remodeling of the BM interactome during aging. (A, B) Chord diagrams displaying unique interactions specific to young (A) and aged (B) BM microenvironment cells. (C) Chord diagrams showing interactions involving TGFB1 signaling in the aged BM. Colors and widths represent the signal senders and the strength of interactions, respectively. (D) Chord diagrams showing interactions through MIF, CD74, and CD99 ligands in the aged BM. Colors and widths represent the signal senders and the strength of interactions, respectively. [file ACEL-25-e70475-s013.pdf]

*YBX1*

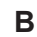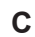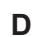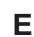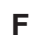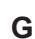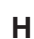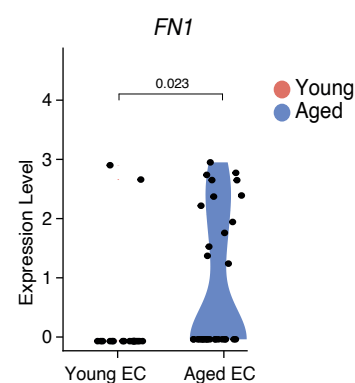

Supplement: Supplementary file 12 — Figure S12: Additional information about the spatial expression patterns of aging‐related gene expression changes in EC. (A) Spatial expression patterns of the transcription factor YBX1 in young (left side) and elderly (right side) BM samples. (B) Violin plots illustrating the spatial expression levels of CLU and CD74 genes in young (red) and aged (blue) EC. (C) Box plots showing the scores for oxidative metabolism genes set in young (red) and aged (blue) EC. (D) Violin plots illustrating the spatial expression levels of VIM and GSN genes in young (red) and aged (blue) EC (E) Violin plots showing BMPR2 spatial gene expression levels in young (red) and aged (blue) EC. (F) Spatial expression patterns of the JAG1‐NOTCH3 L‐R pair in EC golden spots of young (left panels) and elderly (right panels) BM samples. (G) Plots showing the correlation between EC signature and DC signature of EC‐labeled spots in young (left‐red) and elderly (right‐blue) samples. (H). Violin plots of FN1 spatial expression levels in young (red) and aged (blue) EC. [file ACEL-25-e70475-s019.pdf]

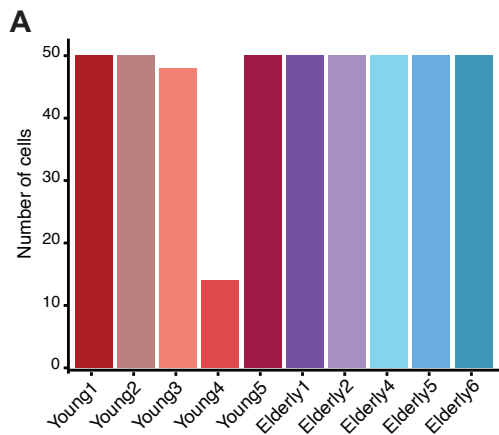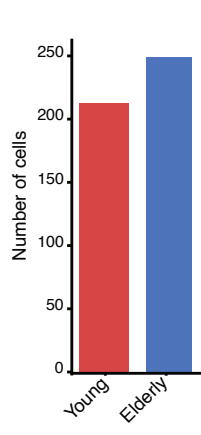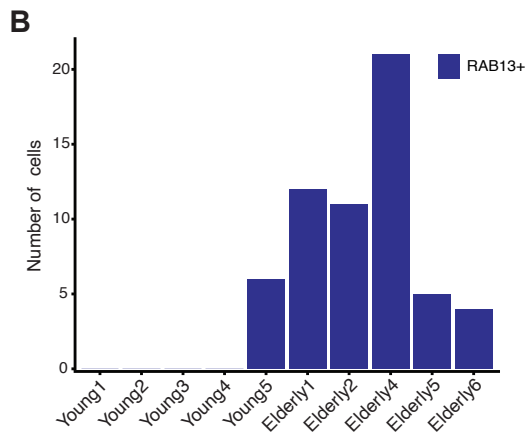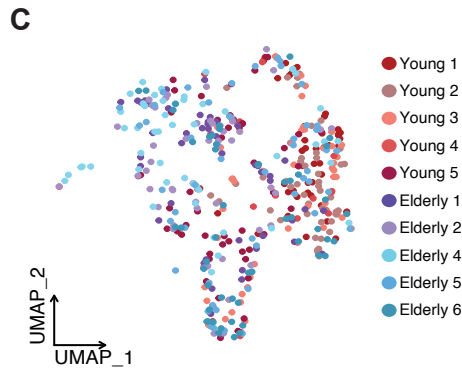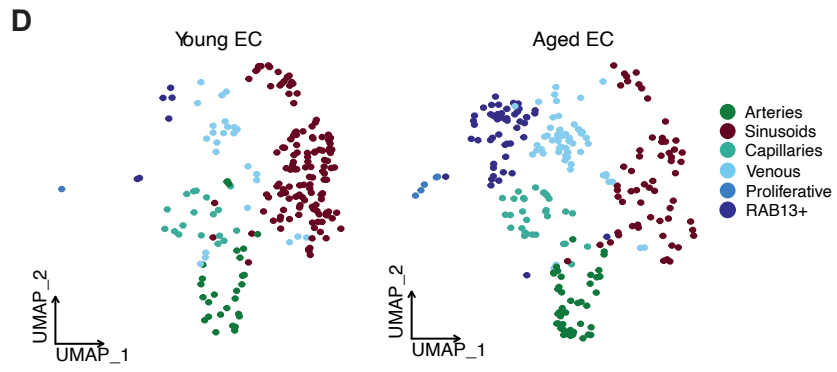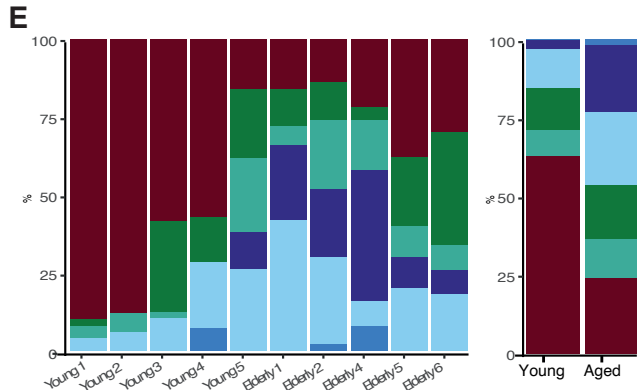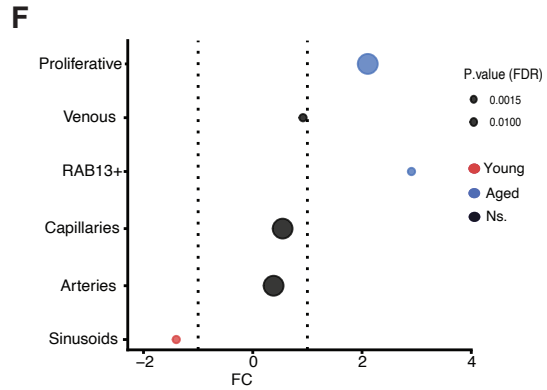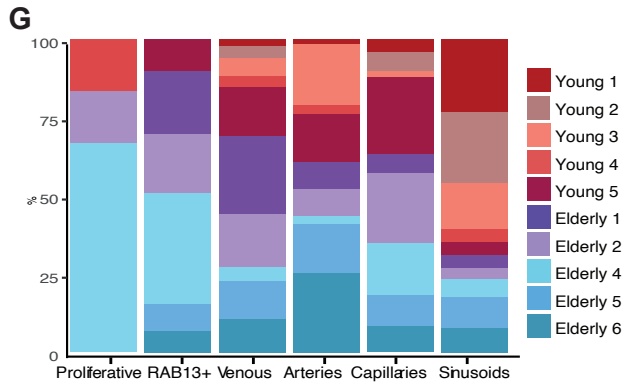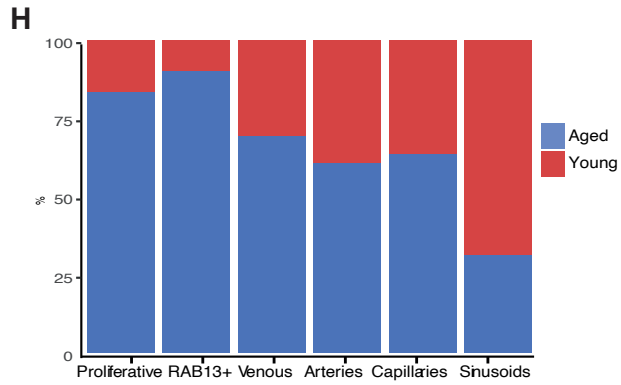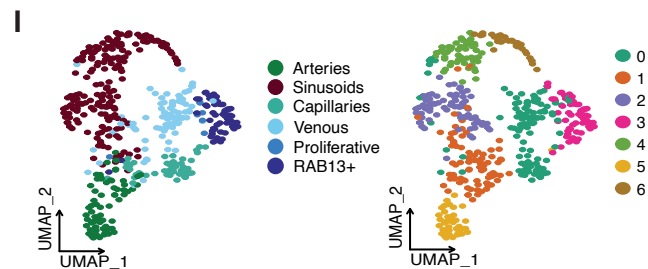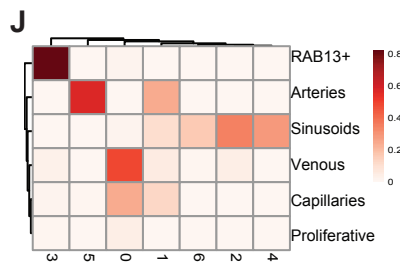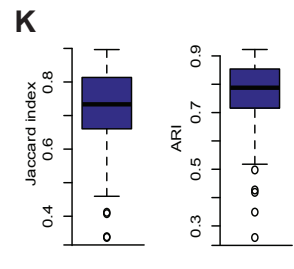

Supplement: Supplementary file 13 — Figure S13: Donor‐balanced bootstrap analysis of EC subtypes showing one of the iterations as an example. (A) Number of EC sampled during balanced downsampling (n = 50 cells per donor) grouped per donor (left) and age group (right). (B) Number of RAB13+ EC per individual after downsampling. (C) UMAP embedding of downsampled EC colored by donor identity. (D). UMAP representation of downsampled EC split into young and aged group, colored by subtype. (E) Stacked bar plots representing the proportion of each EC subtype per individual (left panel) and age group (right panel). (F) Relative differences in cell proportions for each vascular state, comparing young and aged EC. Red and blue represent clusters statistically significant (FDR < 0.05 and absolute log2 fold change > 1) in young and aged EC, respectively. (G,H) Stacked bar plots representing the proportion of cells in each EC subtype per individual (G) and age group (H). (I) UMAP plot illustrating the recomputed dimensional reduction and clustering of downsampled EC colored by subtype (left) and cluster (right). (J) Heatmap showing Jaccard similarity index between the recomputed clusters (X axis) and EC subtypes (Y axis). (K) Clustering robustness metrics across iterations, including Jaccard similarity (left) and Adjusted Rand Index (ARI) (K), demonstrating stability of the RAB13+ EC cluster. [file ACEL-25-e70475-s001.pdf]

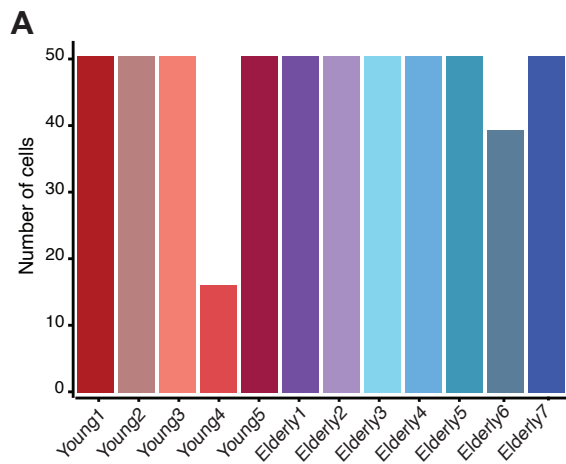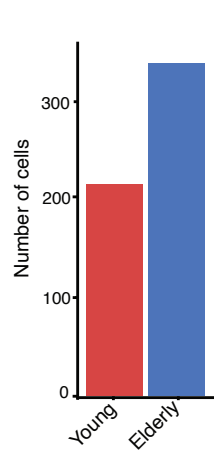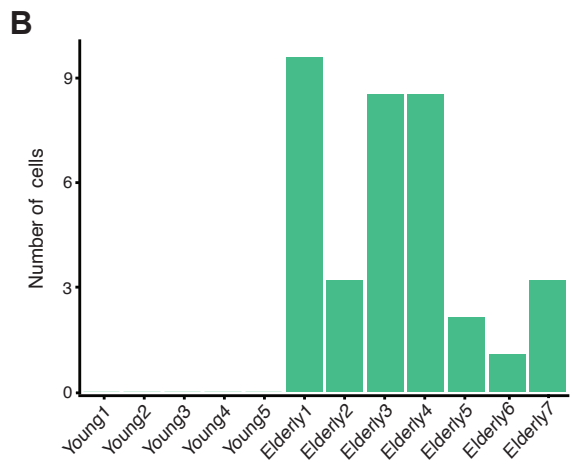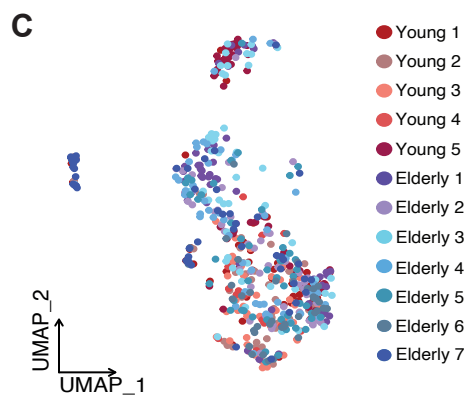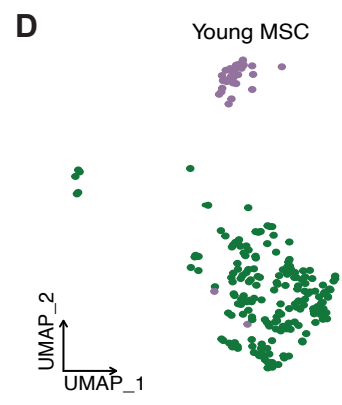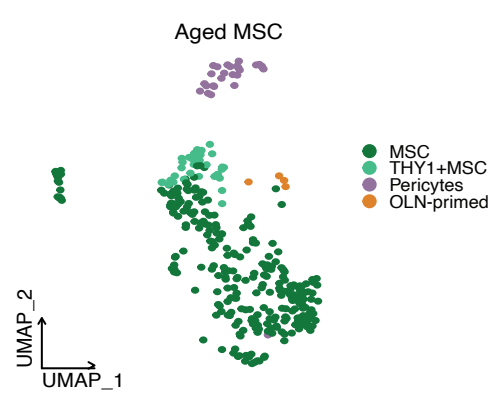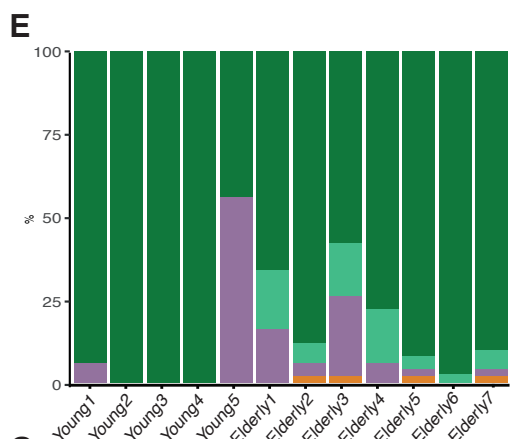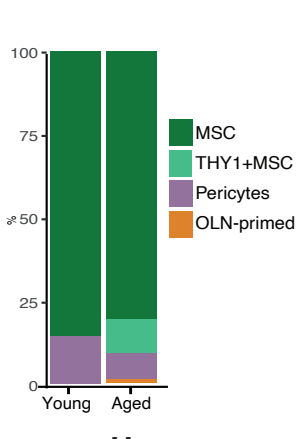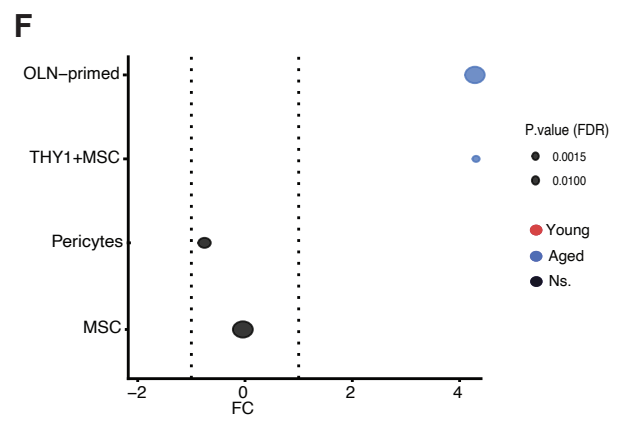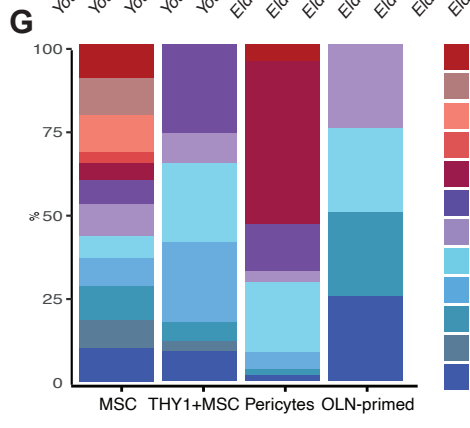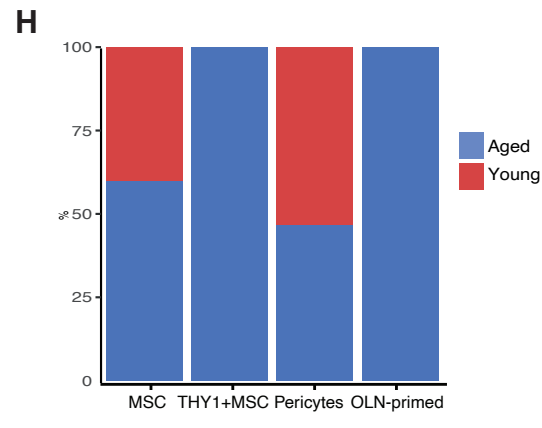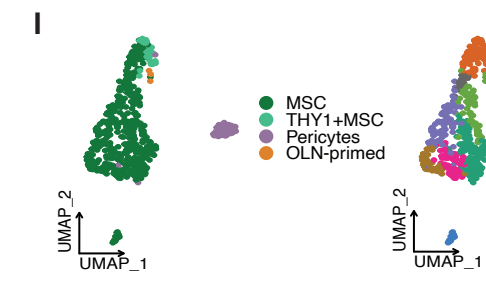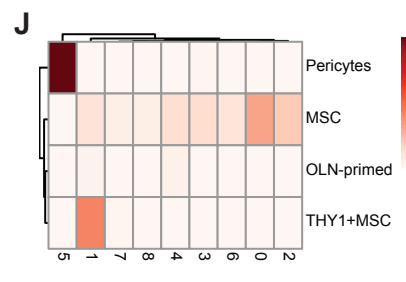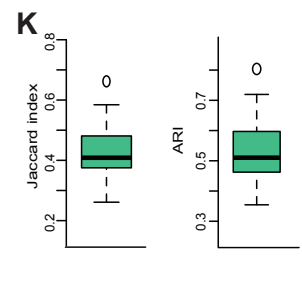

Supplement: Supplementary file 14 — Figure S14: Donor‐balanced bootstrap analysis of MSC subtypes showing one of the iterations as an example. (A) Number of MSC sampled during balanced downsampling (n = 50 cells per donor) grouped per donor (left) and age group (right). (B) Number of THY1+ Fibro‐MSC per individual after downsampling. (C) UMAP plot illustrating the distribution downsampled MSC colored by individual. (D) UMAP representation of downsampled MSC split into young and aged group, colored by subtype. (E) Stacked bar plots representing the proportion of each MSC subtype per individual (left panel) and age group (right panel). (F) Relative differences in cell proportions for each MSC population, comparing young and aged MSC. Red and blue represent clusters statistically significant (FDR < 0.05 and absolute log2 fold change > 1) in young and aged MSC, respectively. (G, H) Stacked bar plots representing the proportion of cells in each MSC subtype per individual (G) and age group (H). (I) UMAP plot illustrating the recomputed dimensional reduction and clustering of downsampled MSC colored by subtype (left) and cluster (right). (J) Heatmap showing Jaccard similarity index between the recomputed clusters (X axis) and MSC subtypes (Y axis). (K) Clustering robustness metrics across iterations, including Jaccard similarity (left) and Adjusted Rand Index (ARI) (K), demonstrating stability of the THY1+ fibro‐MSC cluster. [file ACEL-25-e70475-s025.pdf]

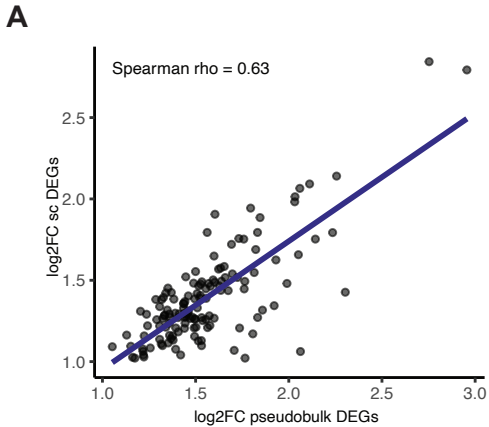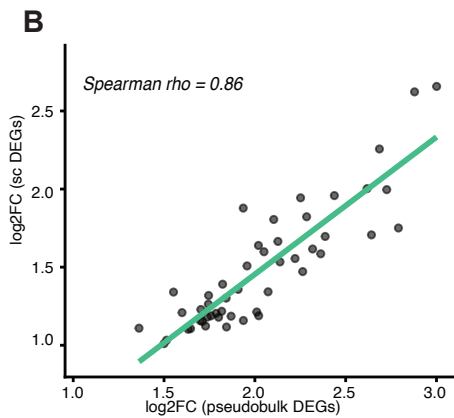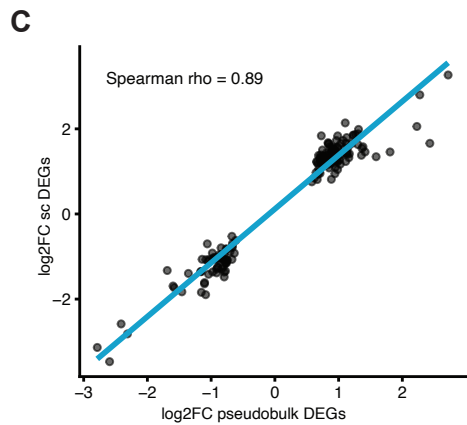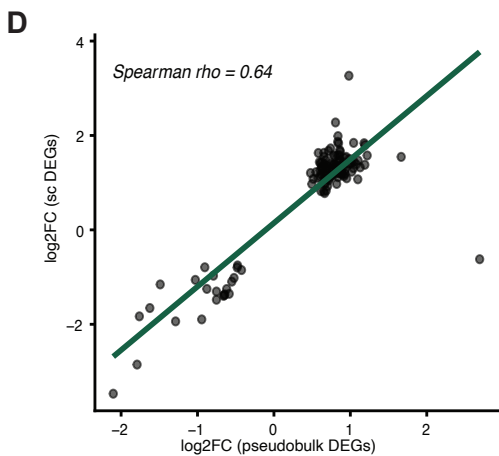

Supplement: Supplementary file 15 — Figure S15: Concordance between donor‐level and single‐cell differential expression analysis. (A) Correlation of log2 fold‐change estimates for common RAB13+ EC marker genes between single‐cell and pseudobulk analyses. (B) Correlation of log2 fold‐change estimates for common THY1+ fibro‐MSC marker genes between single‐cell and pseudobulk analyses. (C) Correlation of log2 fold‐change estimates for common age‐associated DEGs between single‐cell and pseudobulk analyses (Spearman ρ = 0.89). (D) Correlation of log2 fold‐change estimates for common age‐associated DEGs between single‐cell and pseudobulk analyses (Spearman ρ = 0.89). [file ACEL-25-e70475-s016.pdf]

**A**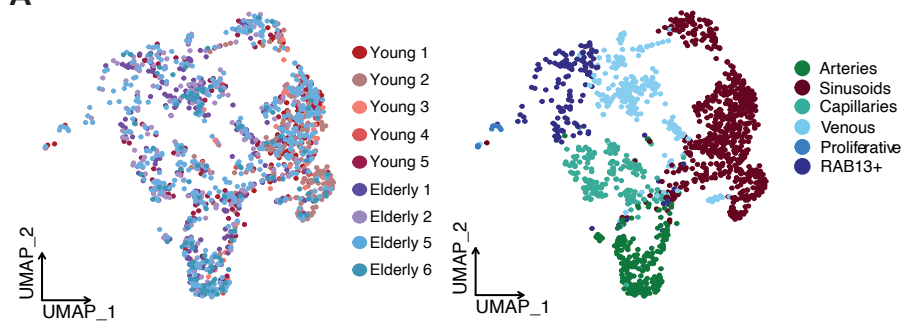**B**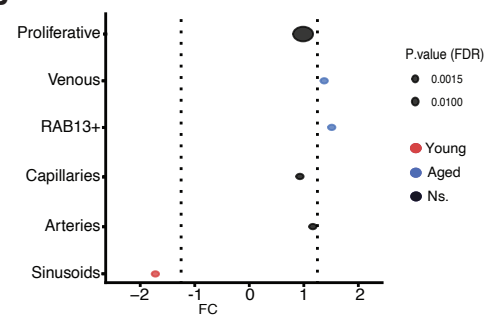**C**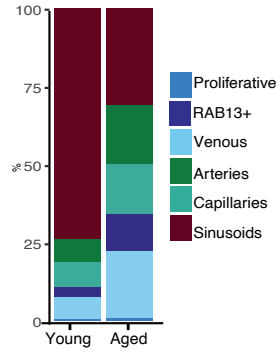**D**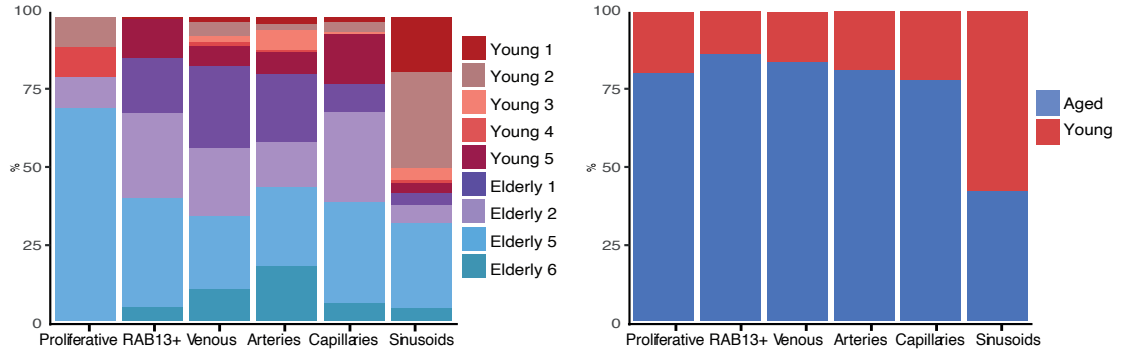**E**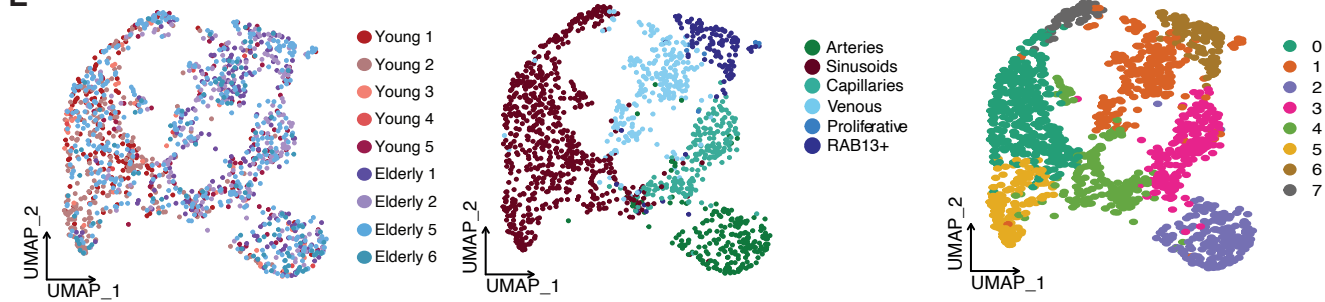**F**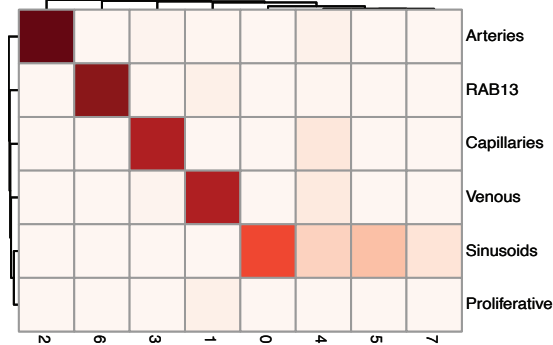**G**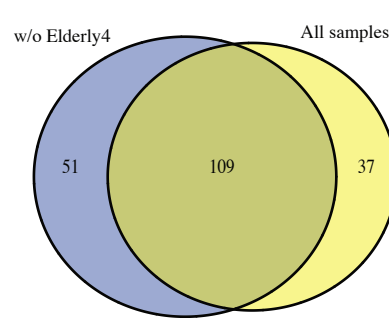

Supplement: Supplementary file 16 — Figure S16: Sensitivity analysis of endothelial cell populations after exclusion of dominant donor Elderly 4. (A) UMAP projection of EC after exclusion of donor Elderly 4, colored by donor identity (left) and endothelial subtypes (right). (B) Differential abundance analysis showing fold changes in endothelial subtypes between aged and young donors following donor exclusion, with FDR‐adjusted p‐values indicated. (C) Stacked bar plots representing the proportion of each EC subtype per age group. (D) Stacked bar plots representing the proportion of cells in each EC subtype per individual (left) and age group (right). (E) Recomputed UMAP embeddings after donor exclusion colored by donor identity (left), endothelial subtype (middle), and cluster assignment (right). (F) Heatmap of cluster correspondence between the endothelial subtypes from the original dataset and the re‐assigned clusters. (G) Venn diagram showing overlap of RAB13+ EC marker genes identified in the original dataset versus after exclusion of Elderly 4. [file ACEL-25-e70475-s002.pdf]

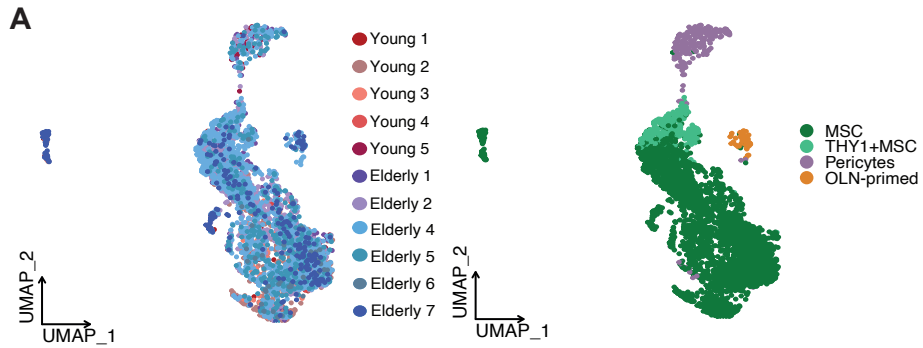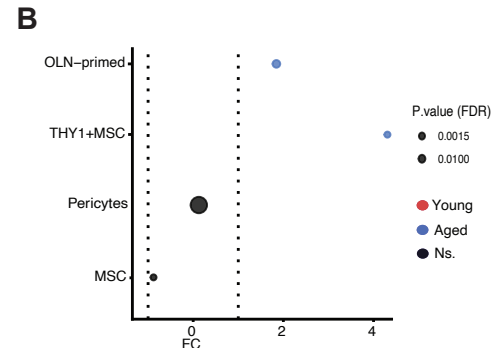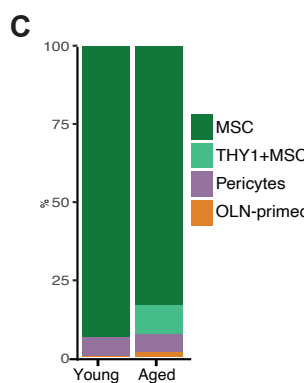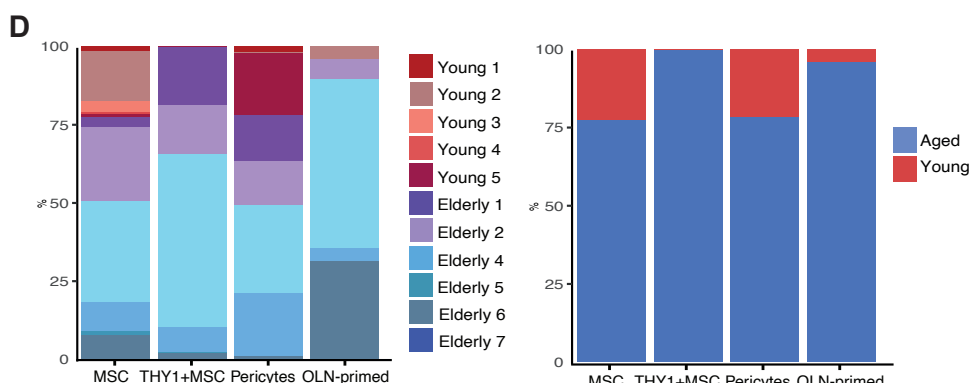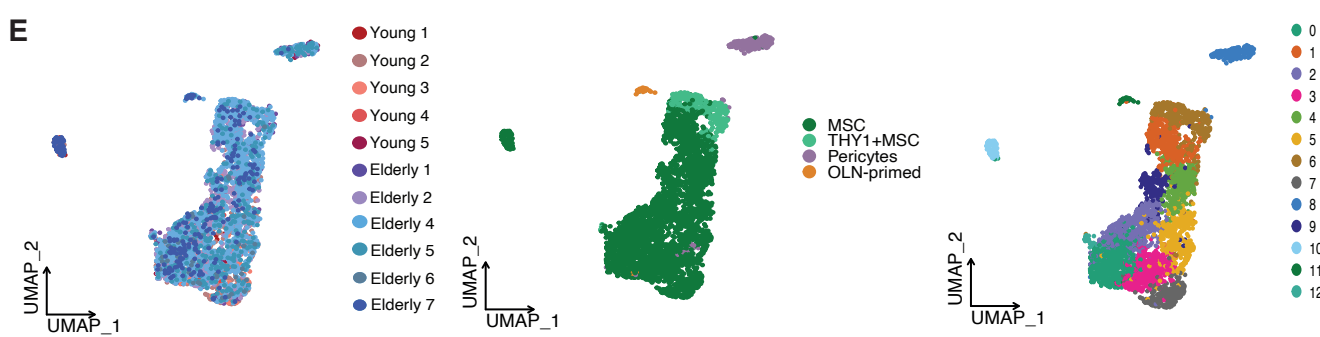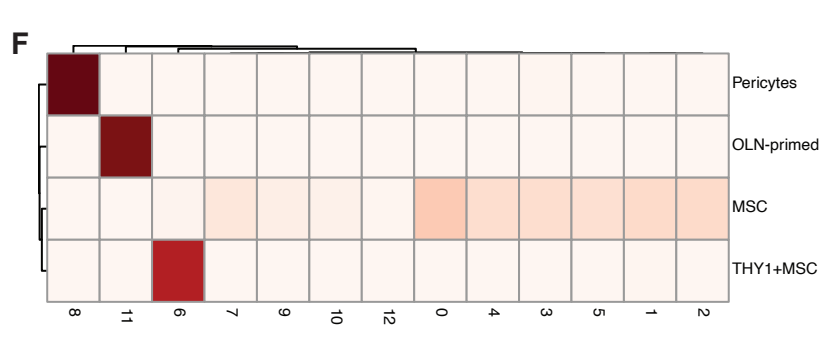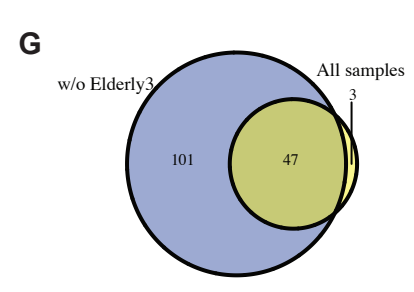

Supplement: Supplementary file 17 — FIGURE S17: Sensitivity analysis of endothelial cell populations after exclusion of dominant donor Elderly 3. (A) UMAP projection of MSC after exclusion of donor Elderly 3, colored by donor identity (left) and mesenchymal subtypes (right). (B) Differential abundance analysis showing fold changes in mesenchymal subtypes between aged and young donors following donor exclusion, with FDR‐adjusted p‐values indicated. (C) Stacked bar plots representing the proportion of each mesenchymal subtype per age group. (D) Stacked bar plots representing the proportion of cells in each mesenchymal subtype per individual (left) and age group (right). (E) Recomputed UMAP embeddings after donor exclusion colored by donor identity (left), mesenchymal subtype (middle), and cluster assignment (right). (F) Heatmap of cluster correspondence between the mesenchymal subtypes from the original dataset and the re‐assigned clusters. (G) Venn diagram showing overlap of THY1+ fibro‐MSC marker genes identified in the original dataset versus after exclusion of Elderly 3. [file ACEL-25-e70475-s009.pdf]
