# Supplementary material for: Single‐Cell Profiling Reveals RAB13 + Endothelial Cells and Profibrotic Mesenchymal Cells in Aged Human Bone Marrow
Source: Aging Cell. 2026 Apr 9;25(4):e70475. doi: 10.1111/acel.70475 (PMC13063395; doi:10.1111/acel.70475)
Supplement: Supplementary file 8 — Figure S8: Validation of age‐associated enrichment of THY1+ Fibro‐MSC and related pathways. (A) IF staining in the FFPE BM biopsies (Table S5) of the young sample 11 (left) and the elderly sample 14 (right). Scale bars: 50 μm. Fibronectin (green), CD271 (red), and DAPI (blue). Green and red arrows indicate colocalization of fibronectin MSC (fibronectin+ CD271+). (B) IF staining in the FFPE BM biopsies (Table S5) of the young sample 11 (left) and the elderly sample 11 (right). Scale bars: 50 μm. Type I collagen (green), CD271 (red), and DAPI (blue). Green and red arrows indicate colocalization of type I collagen MSC (type I collagen+ CD271+). (C) IF staining in the FFPE BM biopsies (Table S5) of the young sample 10 (left) and the elderly samples 8 (right). Scale bars: 50 μm. Phosphorylated SMAD2 (pSMAD2) (green), CD271 (red), and DAPI (blue). Green and red arrows indicate colocalization of pSMAD2 MSC (pSMAD2+ CD271+). (D) Quantification of THY1+ cells (CD90+) (red), MSC (PRRX1+) (green), and THY1+ MSC (coexpression CD90+ and PRRX1+) in young, elderly, and myelofibrosis (MF) samples. Bars represent the mean ± SEM. ns: not significant. (E) Quantification of fibrotic tissue area in young, elderly, and MF samples. The fibrotic tissue area was measured in Masson's trichrome‐stained images by two approximations. The percent of fibrotic tissue area/whole tissue area and the circularity of the cells. Bars represent the mean ± SEM. ns: not significant. (F) Left panel: IF staining of THY1+ Fibro stromal cells (CD90+) (red), (PRRX1+) (green), and nucleus (DAPI) (blue) in FFPE biopsy samples from MF patients. Scale bars: 100 μm. Right panel: Masson‐Trichrome staining for fibrotic tissue of FFPE biopsies from the same tissue area. White arrows indicate THY1+ Fibro MSC (PRRX1+ CD90+). [file ACEL-25-e70475-s008.pdf]

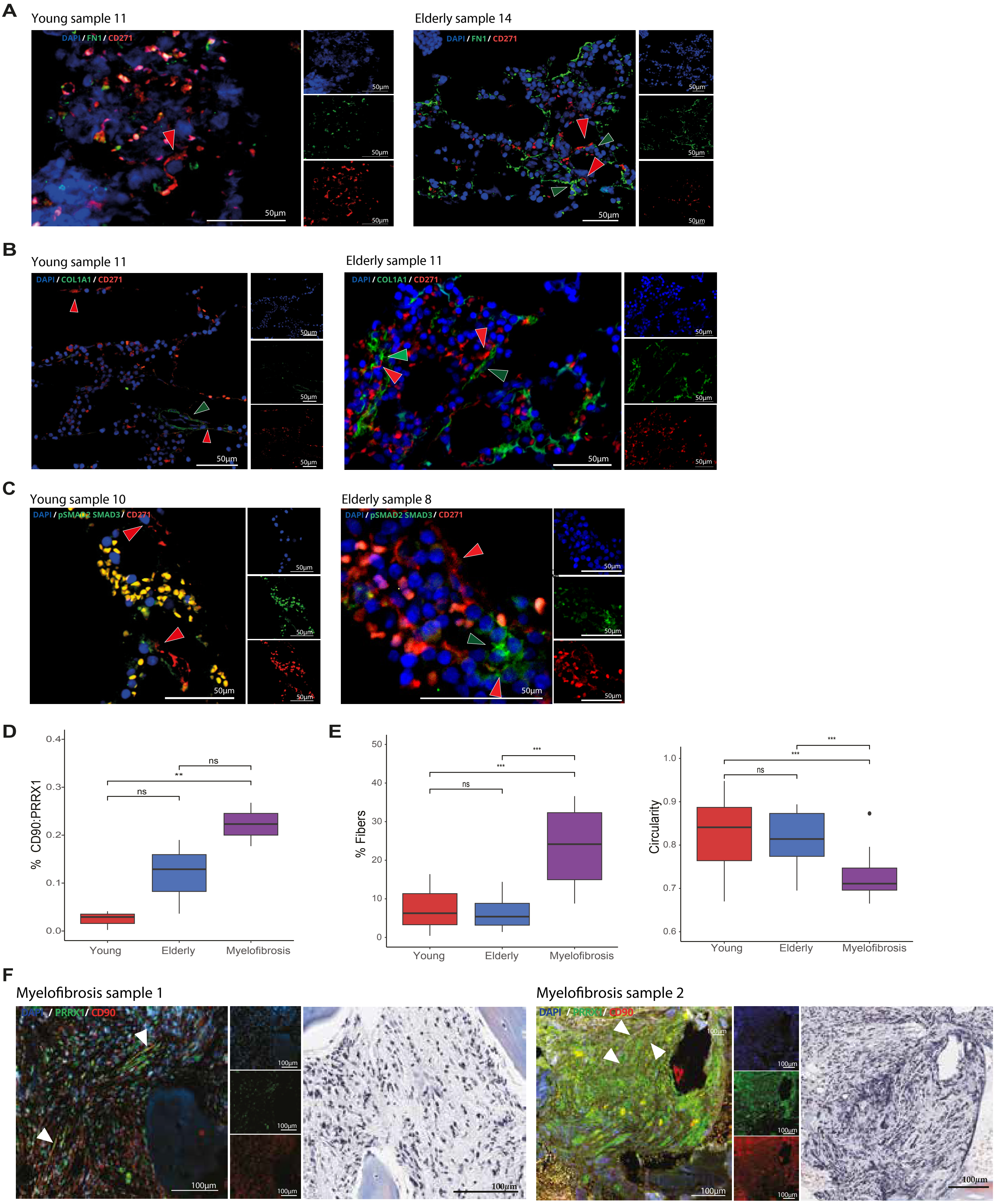

A
Young sample 11
DAPI / FN1 / CD271
50µm
Elderly sample 14
DAPI / FN1 / CD271
50µm
B
Young sample 11
DAPI / COL1A1 / CD271
50µm
Elderly sample 11
DAPI / COL1A1 / CD271
50µm
C
Young sample 10
DAPI / pSMAD2 SMAD3 / CD271
50µm
Elderly sample 8
DAPI / pSMAD2 SMAD3 / CD271
50µm
D
% CD90:PRRX1
0.0
0.1
0.2
0.3
0.4
ns
**
ns
Young
Elderly
Myelofibrosis
E
% Fibers
0
10
20
30
40
50
ns
***
***
Young
Elderly
Myelofibrosis
Circularity
0.6
0.7
0.8
0.9
1.0
ns
***
***
Young
Elderly
Myelofibrosis
F
Myelofibrosis sample 1
DAPI / PRRX1 / CD90
100µm
Myelofibrosis sample 2
DAPI / PRRX1 / CD90
100µm
