# Supplementary material for: Single‐Cell Profiling Reveals RAB13 + Endothelial Cells and Profibrotic Mesenchymal Cells in Aged Human Bone Marrow
Source: Aging Cell. 2026 Apr 9;25(4):e70475. doi: 10.1111/acel.70475 (PMC13063395; doi:10.1111/acel.70475)
Supplement: Supplementary file 11 — Figure S11: Additional information about spatial transcriptomics analysis of the human BM. (A) Pie charts illustrating the proportion of each cell type contributing to the transcriptomic signature of each spot in young (left) and elderly (right) BM samples from deconvolution analysis. (B) Pie charts illustrate the proportion of each cell type that contributes significantly to the transcriptomic signature of each spot in young (left) and elderly (right) BM samples. (C) Cell type proportions per spot derived from deconvolution analysis using the Bandyopadhyay et al. (2024) dataset as a reference. (D) Spatial distribution pattern of the signature score for EC (top panels) and MSC (bottom panels) in young (left side) and elderly (right side) BM samples. (E) Correlation between cell‐type proportions obtained through deconvolution (percentage) and the signature scores of EC (top) and MSC (bottom) in young (left side) and elderly (right side) BM samples. (F) Spatial distribution of “golden”, “high”, “low”, and “rest” spots based on the top‐ranking overlap between deconvolution and spot signature analyses (detailed in the methods section) in young (left side) and elderly (right side) BM samples using EC (top panels) and MSC (bottom panels) as examples. (G) H&E staining of young (upper panel) and elderly (bottom panel) BM samples. [file ACEL-25-e70475-s005.pdf]

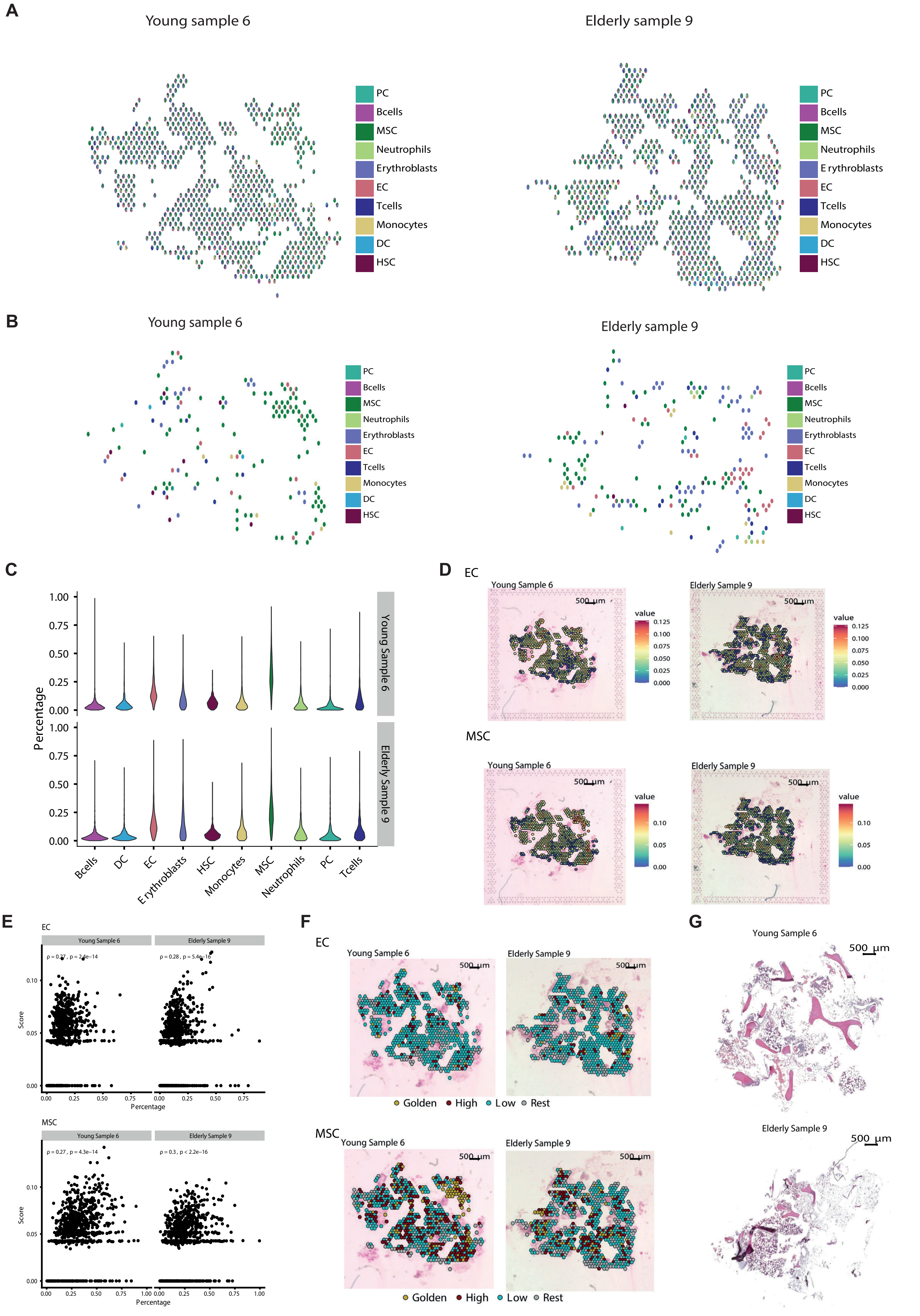
A
Young sample 6
Elderly sample 9
PC
Bcells
MSC
Neutrophils
Erythroblasts
EC
Tcells
Monocytes
DC
HSC
B
Young sample 6
Elderly sample 9
C
Percentage
1.00
0.75
0.50
0.25
0.00
Young Sample 6
Elderly Sample 9
Bcells
DC
EC
Erythroblasts
HSC
Monocytes
MSC
Neutrophils
PC
Tcells
D
EC
Young Sample 6
Elderly Sample 9
500 μm
value
0.125
0.100
0.075
0.050
0.025
0.000
MSC
0.10
0.05
0.00
E
EC
Young Sample 6
Elderly Sample 9
Score
Percentage
MSC
ρ = 0.27 , p = 4.3e−14
ρ = 0.3 , p < 2.2e−16
F
EC
Young Sample 6
Elderly Sample 9
500 μm
Golden
High
Low
Rest
MSC
G
Young Sample 6
500 μm
Elderly Sample 9
500 μm
